# Genetic contributions to variation in general cognitive function: a meta-analysis of genome-wide association studies in the CHARGE consortium (*N*=53 949)

**DOI:** 10.1038/mp.2014.188

**Published:** 2015-02-03

**Authors:** G Davies, N Armstrong, J C Bis, J Bressler, V Chouraki, S Giddaluru, E Hofer, C A Ibrahim-Verbaas, M Kirin, J Lahti, S J van der Lee, S Le Hellard, T Liu, R E Marioni, C Oldmeadow, I Postmus, A V Smith, J A Smith, A Thalamuthu, R Thomson, V Vitart, J Wang, L Yu, L Zgaga, W Zhao, R Boxall, S E Harris, W D Hill, D C Liewald, M Luciano, H Adams, D Ames, N Amin, P Amouyel, A A Assareh, R Au, J T Becker, A Beiser, C Berr, L Bertram, E Boerwinkle, B M Buckley, H Campbell, J Corley, P L De Jager, C Dufouil, J G Eriksson, T Espeseth, J D Faul, I Ford, Generation Scotland, R F Gottesman, M E Griswold, V Gudnason, T B Harris, G Heiss, A Hofman, E G Holliday, J Huffman, S L R Kardia, N Kochan, D S Knopman, J B Kwok, J-C Lambert, T Lee, G Li, S-C Li, M Loitfelder, O L Lopez, A J Lundervold, A Lundqvist, K A Mather, S S Mirza, L Nyberg, B A Oostra, A Palotie, G Papenberg, A Pattie, K Petrovic, O Polasek, B M Psaty, P Redmond, S Reppermund, J I Rotter, H Schmidt, M Schuur, P W Schofield, R J Scott, V M Steen, D J Stott, J C van Swieten, K D Taylor, J Trollor, S Trompet, A G Uitterlinden, G Weinstein, E Widen, B G Windham, J W Jukema, A F Wright, M J Wright, Q Yang, H Amieva, J R Attia, D A Bennett, H Brodaty, A J M de Craen, C Hayward, M A Ikram, U Lindenberger, L-G Nilsson, D J Porteous, K Räikkönen, I Reinvang, I Rudan, P S Sachdev, R Schmidt, P R Schofield, V Srikanth, J M Starr, S T Turner, D R Weir, J F Wilson, C van Duijn, L Launer, A L Fitzpatrick, S Seshadri, T H Mosley, I J Deary

**Affiliations:** 1 Centre for Cognitive Ageing and Cognitive Epidemiology, University of Edinburgh, Edinburgh, UK; 2 Department of Psychology, University of Edinburgh, Edinburgh, UK; 3 School of Mathematics and Statistics, University of Sydney, Sydney, NSW, Australia; 4 Cardiovascular Health Research Unit, Department of Medicine, University of Washington, Seattle, WA, USA; 5 Human Genetics Center, School of Public Health, University of Texas Health Science Center at Houston, Houston, TX, USA; 6 Inserm-UMR744, Institut Pasteur de Lille, Unité d'Epidémiologie et de Santé Publique, Lille, France; 7 Department of Neurology, Boston University School of Medicine, Boston, MA, USA; 8 K.G. Jebsen Centre for Psychosis Research and the Norwegian Centre for Mental Disorders Research (NORMENT), Department of Clinical Science, University of Bergen, Bergen, Norway; 9 Dr Einar Martens Research Group for Biological Psychiatry, Center for Medical Genetics and Molecular Medicine, Haukeland University Hospital, Bergen, Norway; 10 Department of Neurology, Medical University of Graz, Graz, Austria; 11 Institute for Medical Informatics, Statistics and Documentation, Medical University of Graz, Graz, Austria; 12 Department of Neurology, Erasmus University Medical Center, Rotterdam, The Netherlands; 13 Genetic Epidemiology Unit, Department of Epidemiology, Erasmus University Medical Center, Rotterdam, The Netherlands; 14 Centre for Population Health Sciences, University of Edinburgh, Edinburgh, UK; 15 Institute of Behavioural Sciences, University of Helsinki, Helsinki, Finland; 16 Folkhälsan Research Centre, Helsinki, Finland; 17 Max Planck Institute for Human Development, Berlin, Germany; 18 Max Planck Institute for Molecular Genetics, Berlin, Germany; 19 Medical Genetics Section, University of Edinburgh Centre for Genomic and Experimental Medicine, Institute of Genetics and Molecular Medicine, Western General Hospital, Edinburgh, UK; 20 Queensland Brain Institute, The University of Queensland, Brisbane, QLD, Australia; 21 Hunter Medical Research Institute and Faculty of Health, University of Newcastle, Newcastle, NSW, Australia; 22 Department of Gerontology and Geriatrics, Leiden University Medical Center, Leiden, The Netherlands; 23 Netherlands Consortium for Healthy Ageing, Leiden, The Netherlands; 24 Icelandic Heart Association, Kopavogur, Iceland; 25 University of Iceland, Reykjavik, Iceland; 26 Department of Epidemiology, University of Michigan, Ann Arbor, MI, USA; 27 Centre for Healthy Brain Ageing, School of Psychiatry, University of New South Wales, Sydney, NSW, Australia; 28 Menzies Research Institute, Hobart, Tasmania; 29 MRC Human Genetics Unit, Institute of Genetics and Molecular Medicine, University of Edinburgh, Edinburgh, UK; 30 Framingham Heart Study, Framingham, MA, USA; 31 Department of Biostatistics, Boston University School of Public Health, Boston, MA, USA; 32 Rush Alzheimer's Disease Center, Rush University Medical Center, Chicago, IL, USA; 33 Department of Public Health and Primary Care, Trinity College Dublin, Dublin, Ireland; 34 Andrija Stampar School of Public Health, Medical School, University of Zagreb, Zagreb, Croatia; 35 Department of Epidemiology, Erasmus University Medical Center, Rotterdam, The Netherlands; 36 Netherlands Consortium for Healthy Ageing, Leiden, The Netherlands; 37 National Ageing Research Institute, Royal Melbourne Hospital, Melbourne, VIC, Australia; 38 Academic Unit for Psychiatry of Old Age, St George's Hospital, University of Melbourne, Kew, Australia; 39 Department of Neurology, University of Pittsburgh, Pittsburgh, PA, USA; 40 Department of Psychiatry, University of Pittsburgh, Pittsburgh, PA, USA; 41 Department of Psychology, University of Pittsburgh, Pittsburgh, PA, USA; 42 Inserm, U106, Montpellier, France; 43 Université Montpellier I, Montpellier, France; 44 Faculty of Medicine, School of Public Health, Imperial College, London, UK; 45 Brown Foundation Institute of Molecular Medicine for the Prevention of Human Diseases, University of Texas Health Science Center at Houston, Houston, TX, USA; 46 Human Genome Sequencing Center, Baylor College of Medicine, Houston, TX, USA; 47 Department of Pharmacology and Therapeutics, University College Cork, Cork, Ireland; 48 Program in Translational NeuroPsychiatric Genomics, Department of Neurology, Brigham and Women's Hospital, Boston, MA, USA; 49 Harvard Medical School, Boston, MA, USA; 50 Program in Medical and Population Genetics, Broad Institute, Cambridge, MA, USA; 51 Inserm U708, Neuroepidemiology, Paris, France; 52 Inserm U897, Université Bordeaux Segalen, Bordeaux, France; 53 National Institute for Health and Welfare, Helsinki, Finland; 54 Department of General Practice and Primary health Care, University of Helsinki, Helsinki, Finland; 55 Unit of General Practice, Helsinki University Central Hospital, Helsinki, Finland; 56 K.G. Jebsen Centre for Psychosis Research, Norwegian Centre For Mental Disorders Research (NORMENT), Division of Mental Health and Addiction, Oslo University Hospital and Institute of Clinical Medicine, University of Oslo, Oslo, Norway; 57 Department of Psychology, University of Oslo, Oslo, Norway; 58 Survey Research Center, Institute for Social Research, University of Michigan, Ann Arbor, MI, USA; 59 Robertson Center for Biostatistics, Glasgow, UK; 60 Generation Scotland, University of Edinburgh Centre for Genomic and Experimental Medicine, Institute of Genetics and Molecular Medicine, Western General Hospital, Edinburgh, UK; 61 Department of Neurology, Johns Hopkins University School of Medicine, Baltimore, MD, USA; 62 Center of Biostatistics and Bioinformatics, University of Mississippi Medical Center, Jackson, MS, USA; 63 Intramural Research Program National Institutes on Aging, National Institutes of Health, Bethesda, MD, USA; 64 Department of Epidemiology, University of North Carolina Gillings School of Global Public Health, Chapel Hill, NC, USA; 65 Neuropsychiatric Institute, Prince of Wales Hospital, Sydney, NSW, Australia; 66 Department of Neurology, Mayo Clinic, Rochester, MN, USA; 67 Neuroscience Research Australia, Randwick, NSW, Australia; 68 School of Medical Sciences, University of New South Wales, Sydney, NSW, Australia; 69 Technische Universität Dresden, Dresden, Germany; 70 Department of Biological and Medical Psychology, University of Bergen, Bergen, Norway; 71 Kavli Research Centre for Aging and Dementia, Haraldsplass Deaconess Hospital, Bergen, Norway; 72 K.G. Jebsen Centre for Research on Neuropsychiatric Disorders, University of Bergen, Bergen, Norway; 73 Umeå Center for Functional Brain Imaging (UFBI), Umeå University, Umeå, Sweden; 74 Department of Radiation Sciences, Umeå University, Umeå, Sweden; 75 Department of Integrative Medical Biology, Umeå University, Umeå, Sweden; 76 Wellcome Trust Sanger Institute, Wellcome Trust Genome Campus, Cambridge, UK; 77 Institute for Molecular Medicine Finland (FIMM), University of Helsinki, Helsinki, Finland; 78 Department of Medical Genetics, University of Helsinki and University Central Hospital, Helsinki, Finland; 79 Karolinska Institutet, Aging Research Center, Stockholm University, Stockholm, Sweden; 80 Faculty of Medicine, Department of Public Health, University of Split, Split, Croatia; 81 Deparment of Epidemiology, University of Washington, Seattle, WA, USA; 82 Deparment of Health Services, University of Washington, Seattle, WA, USA; 83 Group Health Research Unit, Group Health Cooperative, Seattle, WA, USA; 84 Institute for Translational Genomics and Population Sciences Los Angeles BioMedical Research Institute, Harbor-UCLA Medical Center, Los Angeles, CA, USA; 85 Division of Genetic Outcomes, Department of Pediatrics, Harbor-UCLA Medical Center, Los Angeles, CA, USA; 86 Centre for Molecular Medicine, Institute of Molecular Biology and Biochemistry, Medical University of Graz, Graz, Austria; 87 School of Medicine and Public Health, University of Newcastle, Newcastle, NSW, Australia; 88 Institute of Cardiovascular and Medical Sciences, University of Glasgow, Glasgow, UK; 89 Department of Pediatrics, Harbor-UCLA Medical Center, Los Angeles, CA, USA; 90 Department of Developmental Disability Neuropsychiatry, School of Psychiatry, University of New South Wales, Sydney, NSW, Australia; 91 Department of Cardiology, Leiden University Medical Center, Leiden, The Netherlands; 92 Department of Internal Medicine, Erasmus University Medical Center, Rotterdam, The Netherlands; 93 Division of Geriatrics, Department of Medicine, University of Mississippi Medical Center, Jackson, MS, USA; 94 Durrer Center for Cardiogenetic Research, Amsterdam, The Netherlands; 95 Interuniversity Cardiology Institute of the Netherlands, Utrecht, The Netherlands; 96 Neuroimaging Genetics Group, QIMR Berghofer Medical Research Institute, Brisbane, QLD, Australia; 97 Dementia Collaborative Research Centre, University of New South Wales, Sydney, NSW, Australia; 98 Department of Radiology, Erasmus University Medical Center, Rotterdam, The Netherlands; 99 ARC, Karolinska Institutet, Stockholm and UFBI, Umeå University, Umeå, Sweden; 100 Neuroscience Research Australia, Sydney, NSW, Australia; 101 Faculty of Medicine, University of New South Wales, Sydney, NSW, Australia; 102 Stroke and Ageing Research, Medicine, Southern Clinical School, Monash University, Melbourne, VIC, Australia; 103 Alzheimer Scotland Dementia Research Centre, University of Edinburgh, Edinburgh, UK; 104 Division of Nephrology and Hypertension, Department of Internal Medicine, Mayo Clinic, Rochester, MN, USA; 105 Department of Global Health, University of Washington, Seattle, WA, USA

## Abstract

General cognitive function is substantially heritable across the human life course from adolescence to old age. We investigated the genetic contribution to variation in this important, health- and well-being-related trait in middle-aged and older adults. We conducted a meta-analysis of genome-wide association studies of 31 cohorts (*N*=53 949) in which the participants had undertaken multiple, diverse cognitive tests. A general cognitive function phenotype was tested for, and created in each cohort by principal component analysis. We report 13 genome-wide significant single-nucleotide polymorphism (SNP) associations in three genomic regions, 6q16.1, 14q12 and 19q13.32 (best SNP and closest gene, respectively: rs10457441, *P*=3.93 × 10^−9^, *MIR2113*; rs17522122, *P*=2.55 × 10^−8^, *AKAP6*; rs10119, *P*=5.67 × 10^−9^, *APOE/TOMM40*). We report one gene-based significant association with the *HMGN1* gene located on chromosome 21 (*P*=1 × 10^−6^). These genes have previously been associated with neuropsychiatric phenotypes. Meta-analysis results are consistent with a polygenic model of inheritance. To estimate SNP-based heritability, the genome-wide complex trait analysis procedure was applied to two large cohorts, the Atherosclerosis Risk in Communities Study (*N*=6617) and the Health and Retirement Study (*N*=5976). The proportion of phenotypic variation accounted for by all genotyped common SNPs was 29% (s.e.=5%) and 28% (s.e.=7%), respectively. Using polygenic prediction analysis, ~1.2% of the variance in general cognitive function was predicted in the Generation Scotland cohort (*N*=5487; *P*=1.5 × 10^−17^). In hypothesis-driven tests, there was significant association between general cognitive function and four genes previously associated with Alzheimer's disease: *TOMM40*, *APOE*, *ABCG1* and *MEF2C*.

## Introduction

The systems with which humans face the challenges of the external and internal environments tend to show deterioration in their mean level of efficiency as people age. For example, the immune system,^1^ cardiovascular^2^ and respiratory systems,^3^ renal function^4^ and stress responses^5^ are, on average, not as efficient in old age as they were in young adulthood. When met with a challenge to a system, healthy older people tend to have a reduced reserve capacity compared with younger adults. The nervous system also declines, with sensory function^6, 7^ and motor strength and co-ordination^8^ not at their highest mean level in older age.

The brain's cognitive functions show a heterogeneity of age-related changes. Some capabilities such as vocabulary, some number skills and general knowledge withstand the aging trend and tend (in the absence of neurological disease) to remain intact into older age, although they decline eventually.^9, 10, 11^ These cognitive functions, which rely on the access to stored information, are called crystallized abilities.^12^

By contrast, fluid cognitive abilities, which rely on on-the-spot information processing, show more age-related decline in mean levels.^9, 10, 11, 12, 13, 14^ Through fluid cognitive abilities, the brain equips us to, for example, recognize and recall previously unseen stimuli, and to make associations between previously unrelated stimuli (memory); induce general rules from sets of occurrences and to apply these rules to new situations (reasoning); perform simple, repetitive cognitive tasks accurately and at high speed (processing speed); compute mental two- and three-dimensional transformations of objects and locations (spatial ability); and organize thinking (executive functioning). By comparison with crystallized abilities, tests of fluid cognitive abilities tend to employ unfamiliar and often abstract materials, and draw less on stored knowledge, education and broader enculturation. Each domain of fluid cognitive ability is important and is a major object of research in its own right.^15, 16^ However, when taken together the cognitive domains show an interesting regularity. If we apply a number of cognitive tests that assess diverse cognitive functions to a large and varied sample, the correlations are universally positive; people who tend to do well on one domain of fluid cognitive ability tend to do well in all of the others, although the associations are far from perfect.^17^ This means that some of the observed interindividual variation in any single cognitive domain can be attributed to at least four sources: how good they are generally at all fluid cognitive tests, how good they are at that fluid cognitive domain, how good they are at specific tests within any one domain, and error of measurement.^18^

In the present study, we focus on the variance that crosses a number of different fluid cognitive functions, that is, on general fluid cognitive ability. We examine the genetic contributions to this ability in middle and older age. There are good reasons for doing so. General cognitive ability remains a strong source of cognitive variation in older age, probably accounting for about as much cognitive variation as in young adulthood.^19^ General cognitive function accounts for a substantial proportion of the age-related variance^9, 20^and genetic variance^21, 22^ of people's cognitive test differences in middle and older age. Across adulthood, and especially in older age, lower fluid cognitive ability and greater decline in it across the life course are associated with earlier mortality.^23, 24, 25^ It can also be argued that if mean levels of fluid cognitive tests show decline with age, then people's differences in cognitive functions become more important as they grow older, because the levels of cognitive functioning that remain in older age are nearer to the critical levels that are needed for everyday functions.^26^

To date, behavior genetic research—using twin, adoption and family designs—shows that general cognitive ability is substantially heritable across the life course, from late childhood to old age.^22^ The heritability of general cognitive functioning in old age might decrease slightly from its levels in young and middle adulthood. Candidate gene studies have found that variation in *APOE* genotype is the only reliable individual genetic associate of cognitive function in older age, but that might apply especially to cognitive change rather than cognitive level in old age.^27, 28, 29^ Using the genome-wide complex trait analysis procedure (GCTA), genome-wide association studies (GWAS) found that ~51% (the s.e. was large, at 11%) of the variation in general fluid cognitive function in late middle age and older age could be accounted for by genetic variation that is tagged by single-nucleotide polymorphisms (SNPs) on the Illumina610-Quadv1 chip.^30^ That study was conducted in a total discovery sample of 3511 individuals, with replication in 670 independent individuals. It found no genome-wide significant single SNP associations. From other GWAS studies of complex traits, we now know that this sample size is likely to be too small, by an order of magnitude, to detect genome-wide significant SNPs.^31^

In summary, general fluid cognitive functioning is a key aspect of health in older age. It is important to identify the determinants of its individual differences, both environmental and genetic. To date, studies have been too small to detect the expected small genetic effects. Here we conduct a meta-analysis of GWAS studies of general fluid cognitive functioning in middle and older age from the Cohorts for Heart and Aging Research in Genomic Epidemiology (CHARGE) consortium, with a total of 53 949 individuals.

## Materials and methods

### Participants

This report includes individuals from 31 population-based cohorts participating in the Cohorts for Heart and Aging Research in Genomic Epidemiology consortium (Supplementary Table S1 and Supplementary Information 1 Section 1). All participants were of European ancestry and aged 45 years or older. Exclusion criteria included prevalent dementia and clinical stroke (including self-reported stroke). The total sample size was 53 949 individuals (*N*_men_=23 030, *N*_women_=30 919).

### General fluid cognitive function phenotype

For each of the cohorts, a general fluid cognitive function component phenotype was constructed from a number of cognitive tasks, each testing a different cognitive domain. In order to construct this measure, each cohort was required to have tasks that tested at least three different cognitive domains. Principal component analysis was applied to the cognitive task scores to derive a measure of general cognitive function, which was the score on the first unrotated principal component. Correlations between each test score and the general cognitive function score were calculated for each cohort, to confirm that all cohorts' general cognitive function scores had been scored in the required direction, that is, with a higher test score indicating higher cognitive function. Further details of the cognitive tasks undertaken and of the phenotype construction in each cohort are provided in Supplementary Information 1 Section 2. In summary, there was a clear single component accounting for between 33.7% and 62.3% (mean=49.6%) of the total cognitive test variance in all cohorts.

Cohorts used different batteries of cognitive tests, which means that there will be phenotypic heterogeneity. However, it has been shown that the individual differences found on the general cognitive component derived from different cognitive test batteries are very similar.^32, 33^ Here we give an example of the similarity of the scores obtained when using two different sets of tests to derive the general cognitive ability component. This can be illustrated in the Lothian Birth Cohort 1936, because it has such a large battery of cognitive tests.^34^ Two general fluid-type cognitive function component phenotypes could be derived, each using a different battery of cognitive tests. Of course, only one of these was used in the GWAS study. The first comprised six non-verbal tests from the Wechsler Adult Intelligence Scale-III UK; these were Block Design, Digit Symbol, Symbol Search, Letter-Number Sequencing, Backward Digit Span and Matrix Reasoning. The second contained the Moray House Test, Logical Memory, Spatial Span, Four Choice Reaction Time and Verbal Fluency. These two general cognitive function phenotypes calculated from two non-overlapping batteries of cognitive tests in the Lothian Birth Cohort 1936 had a correlation of *r*=0.79 (*P*<0.001). Thus, we use this as a methodological illustration to show that there is substantial overlap between the general fluid cognitive ability components from different sets of tests.

### Genotyping and quality control

Each cohort applied quality control (QC) measures based on SNP and sample-based missingness, minor allele frequency, Hardy–Weinberg equilibrium, relatedness and evidence of non-Caucasian descent. Cohort-specific thresholds for these QC measures along with details of genotyping, imputation methods and reference panels are detailed in Supplementary Table S2.

### Statistical analyses

#### Genotype–phenotype association analyses

Genotype–phenotype association analyses were performed, using an additive model, on imputed SNP dosage scores within each cohort (Supplementary Figures S1 and S2). Adjustments for age, sex and population stratification, if required, were included in the model. Cohort-specific covariates—for example, site or familial relationships—were also fitted as required. The cohort-specific association results were subjected to QC procedures before meta-analysis. SNPs were excluded based on imputation quality (IMPUTE info<0.4, MACH *r*^2^<0.3, BIMBAM *r*^2^<0.3) and minor allele frequency (<0.5%). Only SNPs that passed these QC criteria in >50% of individuals were included in the meta-analysis (2 478 500 SNPs). A meta-analysis of the 31 cohorts was performed using the METAL package with an inverse variance weighted model implemented and single genomic control applied (http://www.sph.umich.edu/csg/abecasis/Metal). SNP-based results were also compared to published results for educational attainment^35^ (Social Science Genetic Association Consortium) and general cognitive function in childhood^36^ (Childhood Intelligence Consortium).

Gene-based tests of association were performed using the VEGAS software.^37^ In addition, we examined the gene-based findings for association with published candidate genes previously associated with Alzheimer's disease (AD) or neuropathological features of AD and related dementias,^38, 39, 40, 41, 42, 43, 44^ educational attainment^35^ and general cognitive function in childhood.^36^

#### Prediction analyses

In order to perform prediction analyses in Generation Scotland (GS), a meta-analysis was performed, which excluded this cohort. A multi-SNP prediction model was created using the profile scoring method implemented in PLINK.^45^ This uses the effect sizes estimated in the meta-analysis. The GS best-guess imputed data were used for this analysis, an imputation quality threshold of *r*^2^>0.9 was applied, and the remaining SNPs were pruned to remove those in linkage disequilibrium (based on *r*^2^>0.25 within a 200-SNP sliding window). The estimated effect sizes from the meta-analysis for each of these SNPs were then used to calculate a prediction score for each GS individual. A series of prediction scores was created based on the inclusion of SNPs with a range of association *P*-values: all SNPs and SNPs with *P*<0.5, *P*<0.1, *P*<0.05 or *P*<0.01. Linear or logistic regressions of the prediction score and cognitive phenotypes, and some health outcomes previously associated with cognitive function^46^ were performed. We calculated the proportion of phenotypic variance that was predicted by adding the prediction score to a ‘null' model that adjusted for age, sex and population stratification (four principal components). The cognitive phenotypes from GS that were included in the prediction analysis were general cognitive function, general fluid cognitive function, Wechsler Digit Symbol Substitution Task,^47^ Wechsler Logical Memory Test,^48^ Verbal Fluency^49^ and the Mill Hill Vocabulary Scale (junior+senior synonyms).^50^ The health outcomes included in the prediction analysis were self-reported cardiovascular disease, type 2 diabetes and hypertension. Polygenic prediction analyses were also performed in GS for cognitive phenotypes using published GWAS results for AD^39^ (International Genomics of Alzheimer's Project) and educational attainment.^35^

#### Estimation of SNP-based heritability using GCTA analysis

The GCTA program^51^ was used to estimate the proportion of variance explained by all common SNPs for general cognitive function in the Atherosclerosis Risk in Communities Study (ARIC) and Health and Retirement Study (HRS) cohorts. These cohorts were selected to be used for this analysis, because they are two of the largest cohorts in the study. The total numbers of individuals included in these analyses were 6617 for the ARIC cohort and 5976 for the HRS cohort. These totals differ from the single SNP analyses, because close relatives were excluded from this analysis. One individual was excluded from any pair of individuals that had an estimated coefficient of relatedness of >0.025 to ensure that effects due to shared environment were not included. The same covariates were included in the GCTA analyses as for the SNP-based association analyses.

#### Pathway and functional genomic analyses

The following pathway and functional genomic methods were performed: INRICH^52^ and Ingenuity Pathway Analysis (Ingenuity Systems, www.ingenuity.com), and reference was made to The Genotype-Tissue Expression Portal (http://www.gtexportal.org), Regulome DB database^53^ and the Human Brain Transcriptome Project (hbatlas.org).^54^ Full details are given in Supplementary Methods.

## Results

**Figure 1:**
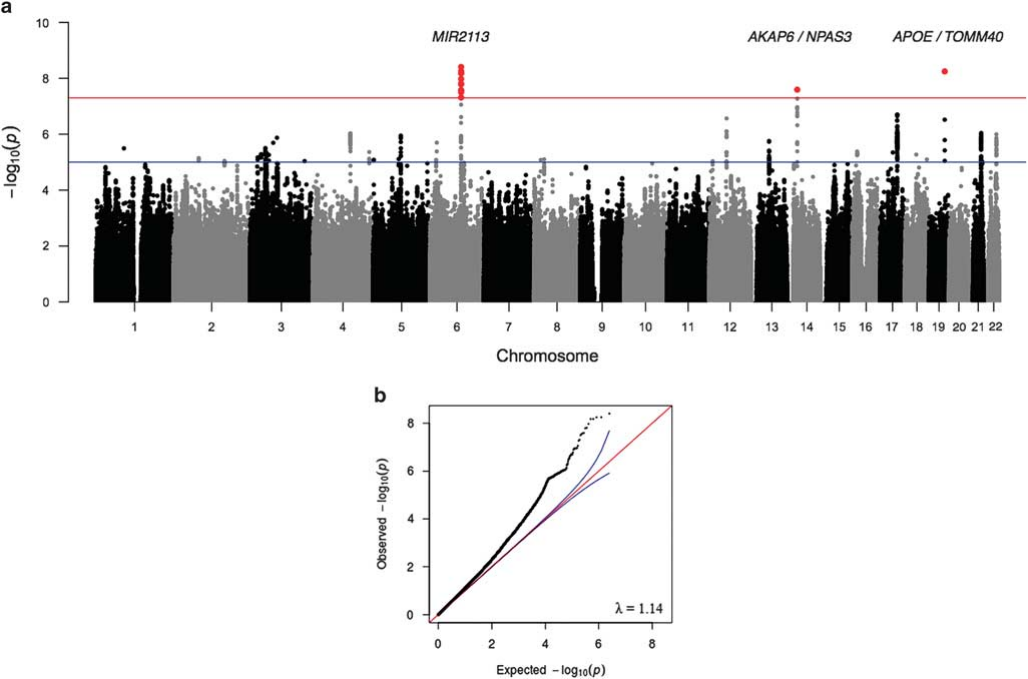
Manhattan (**a**) and Q–Q (**b**) plots of *P*-values of the association between single-nucleotide polymorphisms (SNPs) and general cognitive function in the meta-analysis. The threshold for genome-wide significance (*P*<5 × 10^−8^) is indicated by the red line and the threshold for suggestive significance (*P*<1 × 10^−5^) is indicated by the blue line.

**Figure 2:**
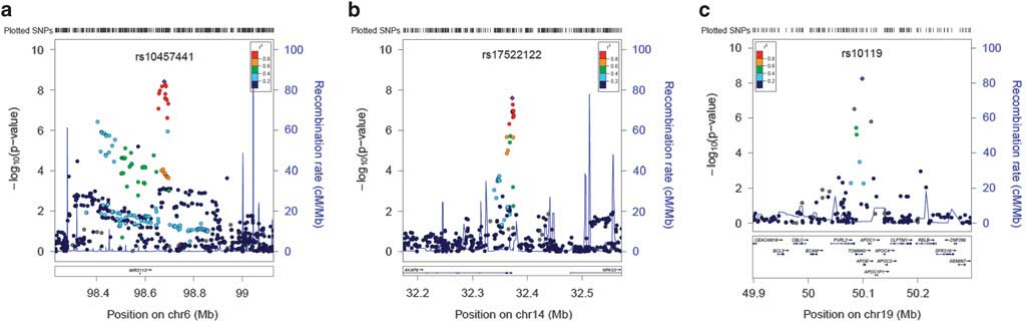
Regional association plots of genomic regions, which demonstrated genome-wide significance (*P*<5 × 10^−8^) in the meta-analysis, for chromosomes 6 (**a**), 14 (**b**) and 19 (**c**). The circles represent each genotyped single-nucleotide polymorphism (SNP), with the color indicating pairwise linkage disequilibrium (LD) in relation to the top hit (calculated from 1000 Genomes Nov 2010 EUR); −log10 *P*-values are also indicated (*y* axis). The purple diamond represents the top SNP in each region. The solid blue line indicates the recombination rate.

**Figure 3:**
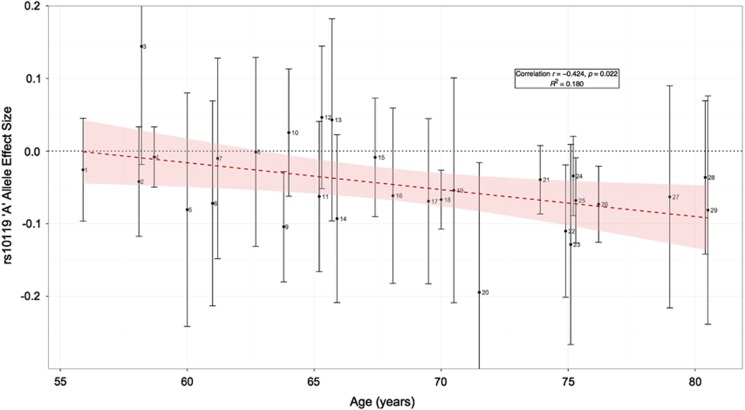
Plot of effect size against mean age of cohort for rs10119 (top SNP *APOE/TOMM40* region). Each numbered point represents a cohort (1, RSIII; 2, ERF; 3, SPLIT; 4, GS; 5, KORCULA; 6, NCNG; 7, GENOA; 8, ORCADES; 9, RSI; 10, FHS; 11, ASPS; 12, BASEII; 13, BETULA; 14, HCS; 15, RSII; 16, HBCS; 17, LBC1936; 18, HRS; 19, OATS; 20, TASCOG; 21, 3C; 22, PROSPER-Netherlands; 23, ROS; 24, PROSPER-Scotland; 25, PROSPER-Ireland; 26, AGES; 27, LBC1921; 28, CHS; 29, MAP). Two cohorts (ARIC and Sydney MAS) did not have data available for rs10119. Dashed regression line and shaded 95% confidence interval are shown. For full details of abbreviations, see Supplementary Information 2: Cohort Abbreviations.

The SNP-based meta-analysis identified 13 SNPs associated with general cognitive function at a genome-wide significance level (*P*<5 × 10^−8^) (Figure 1, Figure 2 and Supplementary Figure S3). These SNPs were located in three genomic regions, 6q16.1, 14q12 and 19q13.32. The top SNP in each region and genes contained in the region were, 6q16.1, rs10457441 (*P*=3.93 × 10^−9^; *MIR2113*), 14q12, rs17522122 (*P*=2.55 × 10^−8^; *AKAP6/NPAS3*) and 19q13.32, rs10119 (*P*=5.67 × 10^−9^; *TOMM40/APOE*) (Figure 2). The effect size of rs10119 was significantly correlated with mean age of the cohort (*r*=−0.424, *P*=0.022; Figure 3 and Supplementary Figure S4). There was no significant correlation with cohort age for the other two SNPs (rs10457441 and rs17522122) (Supplementary Figures S5 and S6). All 361 SNPs from the meta-analysis with a *P*-value less than a suggestive significance threshold of *P*<1 × 10^−5^ are detailed in Supplementary Table S3.

Gene-based tests of association yielded one genome-wide significant result (*P*<2.8 × 10^−6^), for the gene *HMGN1* (*P*=1 × 10^−6^) located on chromosome 21. All 184 gene-based association results with a *P*-value below the suggestive threshold (*P*<1 × 10^−3^) are detailed in Supplementary Table S4.

Supplementary Table S5 shows the gene-based association results for 29 genes previously reported to be associated with AD or neuropathologic features of AD and related dementias.^38, 39, 40, 41, 42, 43, 44^ Four of these genes, *TOMM40*, *APOE*, *MEF2C* and *ABCG1*, were associated with general fluid cognitive function at *P*<0.01. Owing to linkage disequilibrium, the association *P*-values for *APOE* and *TOMM40* are not independent. *APOE* and *TOMM40* are located in the region of chromosome 19 identified by the SNP-based meta-analysis.

Supplementary Tables S6A and S7A show the top SNP-based results from published GWAS of educational attainment^35^ and general cognitive function in childhood.^36^ It should be noted that these are not independent studies due to sample overlap in some cohorts between the current study and both of these previously published studies (overlaps are: educational attainment *N*~30 000; general cognitive function in childhood *N*~1500). Of the 361 suggestively significant SNPs from the current meta-analysis (Supplementary Table S3), 188 and 192 SNPs demonstrated nominal significance (*P*<0.05) with the educational attainment phenotypes of years of education and college completion, respectively (Supplementary Table S7B). Sixteen SNPs achieved *P*<1 × 10^−6^ in the educational attainment analyses^35^; six of these achieved nominal significance in the current meta-analysis (Supplementary Table S7A). Of the top 100 SNPs in the general cognitive function in childhood GWAS,^36^ 11 reached a nominal level of significance in the current study (Supplementary Table S6A). Comparisons of gene-based results are shown in Supplementary Tables S6B, S7C and S7D. For the educational attainment phenotypes, 17 and 14 of the top 25 genes associated with years of education and college completion,^35^ respectively, were nominally significant in the current gene-based results. For childhood general cognitive function,^36^ eight of the top 20 gene-based findings achieve nominal significance in the current study.

Results from the meta-analysis are consistent with a polygenic model of inheritance. This is demonstrated by the clear early deviation from the null hypothesis observed in the QQ plot (*λ*=1.14) (Figure 1). To investigate further whether the general cognitive phenotype is under polygenic control as is indicated by the association meta-analyses, we applied the analysis within the GCTA set of methods^51^ that estimates the proportion of phenotypic variance explained by all SNPs in two of the largest single cohorts: ARIC and HRS. For the ARIC and HRS cohorts, respectively, the GCTA method returned SNP heritability estimates of 0.29 (s.e.=0.05; *N*=6617; *P*=2.34 × 10^−9^) and 0.28 (s.e.=0.07; *N*=5976; *P*=2.00 × 10^−5^).

The results from the polygenic prediction analyses are shown in Supplementary Table S8. The maximum proportion of phenotypic variance explained in GS using the prediction set derived from the meta-analysis excluding GS was 1.27% (*P*=1.5 × 10^−17^) for general cognitive function when using the *P*<0.50 SNP set (*N*=47 322). The proportion of variance explained in other cognitive domains ranged from near-zero values to about 1% (Supplementary Table S8). The polygenic score did not significantly predict cardiovascular disease, hypertension or type 2 diabetes in GS (all *P*>0.01). Supplementary Table S9 shows the results from the polygenic prediction using the published results for educational attainment^35^ (years of education and completion of a college degree) and AD.^39^ For educational attainment, the maximum proportion of phenotypic variance explained in GS for the general cognitive phenotype was 0.54% (*P*=2.78 × 10^−8^) when using the *P*<0.50 SNP set (*N*=40 239). For AD, significant predictions were observed for only the more inclusive SNP sets (*P*<0.50 and *P*<1) and the maximum proportion of phenotypic variance explained was 0.19%. Owing to the interdependency of the cognitive function measures, health outcomes and polygenic scores, no correction for multiple testing was applied to the *P*-values presented.

Supplementary Table S10 lists Gene Ontology gene sets that showed nominal enrichment before correction for multiple testing in the INRICH analysis. None of the Gene Ontology gene sets remained significant after correction for multiple testing. The highest ranked 70-node network in the Ingenuity Pathway Analysis included 58 molecules with *CDK2* as a central hub (Supplementary Figure S7). The second-ranked 70-node network included 38 molecules and had three multi-connected hubs: *RHOA*, *NUPR1* and *SRF.* The highest ranked 140-node network includes 103 molecules and combines the top two 70-node networks (Supplementary Figure S8). The top function categories associated with this network are cell cycle, cell death and survival, and gene expression. The top canonical pathways were inositol pyrophosphates biosynthesis, tRNA charging, Ga12/13 signaling, IL-15 production and role of NFAT in regulation of the immune response.

Using the Genotype-Tissue Expression Portal database (http://www.gtexportal.org), no *cis*–expression quantitative trait loci associations were identified for the 13 genome-wide significant SNPs. Supplementary Figure S9 shows differential expression of the top two genes from the VEGAS analysis, *HMGN1* and *BRWD1*, in six brain regions over the human lifecourse (http://hbatlas.org/pages/hbtd). Neither gene demonstrated differential expression across the brain regions shown in middle and older age. For this study, data mining of regulatory elements was restricted to normal brain relevant cell lines/tissues. Supplementary Table S11 demonstrates evidence of regulatory elements associated with 7 of the 13 genome-wide significant SNPs (http://www.regulomedb.org/).

## Discussion

In this genome-wide association study of general cognitive function in middle and older age, with a total *N* of 53 949 participants, we report 13 genome-wide significant SNP-based associations in the three genomic regions 6q16.1, 14q12 and 19q13.32. There was one gene-based significant association with the *HMGN1* gene located on chromosome 21. We observed association of general cognitive function with four genes previously associated with AD or neuropathological features of AD and related dementias (*TOMM40*, *APOE*, *MEF2C* and *ABCG1*). The results from the meta-analysis are consistent with a polygenic model of inheritance, indicating that many variants of small effect contribute to the additive genetic influences on general cognitive function. Using GCTA, we present consistent estimates of the lower bound of the narrow sense heritability of general fluid cognitive function, 0.29 and 0.28, from two of the largest cohorts (ARIC and HRS). We are able to predict, using only common SNP data to create a polygenic score, ~1.2% of the variance in general cognitive function in an independent sample (GS). Pathway and network analyses did not produce significant findings. None of the 13 SNPs achieving genome-wide significance were coding variants. However, functional annotation provided evidence of regulatory elements for seven SNPs, suggesting that they might have a functional non-protein coding effect.

The 19q13.32 region, which includes the *APOE* ɛ2/3/4 haplotype and was associated with general cognitive function in this study, has previously been associated with cognitive phenotypes in old age,^55, 56, 57, 58^ AD^42, 59, 60, 61^ and non-pathological cognitive aging.^28, 29^ Here we find that the *APOE/TOMM40* region is also associated with general cognitive function in middle and older age. The only published GWAS of general cognitive function in older age, to date, did not report any significant *APOE/TOMM40* findings.^30^ From the data presented here, it is not possible to identify a single SNP or gene within this region that is driving the association, as it is a gene-dense region that is known to exhibit a strong pattern of linkage disequilibrium. Davies *et al.*^28^ performed a fine-mapping analysis of this region, which indicated that the observed association with non-pathological cognitive aging was being driven by *APOE*-based variation.^28^ A functional analysis of the *APOE* locus, including *TOMM40*, found that multiple *APOE* locus *cis*-regulatory elements influence both *APOE* and *TOMM40* promoter activity.^62^ Functional annotation of the top SNP (rs10119) in the present study demonstrated evidence of regulatory elements, indicating regions of active transcription and epigenetic modification. All of these factors suggest that there is a complex transcriptional regulatory structure modulating regional gene expression at the *APOE/TOMM40* locus. Drawing on the large number of cohorts in the present paper, we found a significant correlation between mean cohort age and the effect size of rs10119 (located in the *APOE/TOMM40* region) on general cognitive function in older age. The effect was near to zero at younger mean ages and larger at older ages. This might help toward resolving some of the uncertainty about age moderation of *APOE* ɛ4 status associations with cognitive function that are based on single studies.^63, 64, 65^

The 14q12 region identified by the meta-analysis contains both the *AKAP6* and *NPAS3* genes. *AKAP6* (A kinase (PRKA) anchor protein 6) is highly expressed in various brain regions, and cardiac and skeletal muscle. It is specifically localized to the sarcoplasmic reticulum and nuclear membrane, and is involved in anchoring protein kinase A to the nuclear membrane or sarcoplasmic reticulum.^66^ SNPs within this gene have tentatively been associated with several diseases/traits by GWAS: rs4296166 has been associated with risk of AD at a suggestive level of significance,^67^ rs2383378 was suggestively associated with anorexia nervosa,^68^ rs2300835 was associated with fasting insulin levels in the discovery sample of a GWAS of fasting glycemic traits, but failed to replicate,^69^ and rs1951681 and rs3784178 were suggestively associated with economic and political preferences (environmentalism).^70^

*NPAS3* (neuronal PAS domain protein 3) encodes a member of the basic helix-loop-helix and PAS domain-containing family of transcription factors that is expressed in the brain. The encoded protein is localized to the nucleus and may regulate genes involved in neurogenesis.^71^ It has been associated with brain development and potentially human brain evolution.^72^ A balanced reciprocal translocation t(9,14)(q34.2;q13) that disrupts *NPAS3* was identified in a mother and daughter with schizophrenia and schizophrenia co-morbid with mild learning disability, respectively.^73, 74^ A GWAS has since reported three coding SNPs (rs12434716, c.1654G>C, *P*=0.009; rs10141940, c.2208C>T, *P*=0.01; and rs10142034, c.2262C>G, *P*=0.01) to be associated with schizophrenia.^75^

The genome-wide significant SNPs in the 6q16.1 region are located ~100 kb downstream of an uncharacterized microRNA gene *MIR2113*. This region has been previously associated with bipolar disorder (rs12202969, *P*=1.08 × 10^−8^)^76^ and educational attainment.^35^ Evidence of regulatory elements associated with six of the top SNPs in this region, within normal brain-related tissues and cell lines, was identified using the Regulome DB database. The regulatory elements identified include histone modifications, DNase hypersensitive sites and position weight matrix sites. This evidence suggests that the associated SNPs are in sites of active transcription and could have a regulatory role on transcription.

The significant finding from the gene-based analysis, *HMGN1* (high mobility group nucleosome-binding domain 1), encodes a nucleosome-binding protein that is associated with transcriptionally active chromatin.^77^ *HMGN1* negatively regulates methyl CpG-binding protein 2 (*MeCP2*), a DNA-binding protein that is mutated in the neurodevelopmental disorder Rett syndrome^78^ and is known to affect neurological functions including X-linked mental retardation, various autism spectrum disorders in humans and the behavior of mice. It is overexpressed in Down syndrome and it has been suggested that epigenetic changes resulting from altered *HMGN1* levels could have a role in the etiology of several neurodevelopmental disorders including autism and Down syndrome.^79^

Four of the 29 genes previously reported to be associated with AD^38, 39, 40, 41, 42, 44^ or neuropathological features of AD and related dementias^43^ were associated with general cognitive function (at *P*<0.01). These were *TOMM40*, *APOE*, *MEF2C* and *ABCG1.* These results suggest that there is overlap between the genetic contribution to ‘normal' and ‘pathological' cognitive variation in older age. A polygenic prediction analysis using a large published GWAS of AD^39^ significantly predicted only 0.19% of the phenotypic variation in general cognitive function in GS. However, Harris *et al.*^80^ reported no significant association of polygenic score for AD with general cognitive ability when using smaller sample sizes for both the creation of the polygenic score and the prediction analysis. All known cases of clinical dementia were removed from the contributing cohorts. Of course, some or all of the effects we found could be driven by the inadvertent inclusion of individuals in a prodromal stage of dementia, and that we have picked up the genetic effects on this. This is an important issue that is impossible to eliminate entirely. Ideally, one would wish to know, prospectively, which people in the current cohorts eventually developed dementia and then re-run the analyses after omitting them. However, some people will die or be lost to contact before such an assessment could be made, thus preventing complete ascertainment. On the other hand, it is possible to envisage a study that tracks individuals and re-analyses those who have kept in contact and those who do not, say over a 10-year period, develop dementia. This could clarify whether the effects we found here were on the normal range of age-related cognitive change. The present study includes individuals within and beyond the ninth decade. It is also important to note that the cognitive phenotype we measured is a composite of people's stable trait levels and any age-related change that has occurred. Therefore, genetic effects might be contributing to either.

The results of this study (SNP and gene-based) were compared with those of previously published large GWAS of educational attainment^35^ and general cognitive function in childhood.^36^ Before discussing these results further, it should be noted that there is sample overlap between both of these studies and the current study (overlaps are: educational attainment, *N*~30 000; childhood general cognitive function, *N*~1500). Around 50% of the suggestive SNPs from the current study are nominally significant for educational attainment and the 6q16.1 region is reported to be genome-wide significant for both general cognitive function and educational attainment. The gene-based findings also demonstrated some consistency, with more than half of the top 25 genes for years of education and college completion achieving nominal significance in the current study. Of the top 100 SNPs for childhood cognitive function, 11 achieved nominal significance in the current study along with eight of the top 20 gene-based findings. These findings suggest that there is overlap between the genetic contribution to general cognitive function in late-middle and older age, and both educational attainment and childhood general cognitive function. This has also been explored in a study, which used education as a proxy phenotype for general cognitive function.^81^ The bivariate heritability of educational attainment and general cognitive function has been previously estimated in GS using both pedigree-based and SNP-based methods (biv *h*^2^=0.78, *N*~20 500 and biv *h*^2^=0.59, *N*~6600, respectively).^82^ The genetic correlation and bivariate heritability of childhood and older age general cognitive functions have also been previously estimated in a relatively small sample (*N*~1900), (*r*_g_=0.62; biv SNP *h*^2^=0.21).^83^

**Figure 4:**
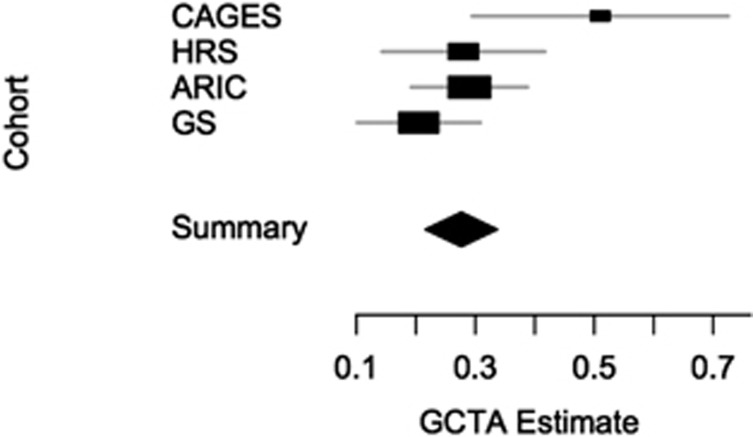
Forest plot of four GCTA-based estimates for the single-nucleotide polymorphism (SNP)-based heritability (*x* axis) of general fluid cognitive function. The summary mean and s.e. were estimated using inverse-variance weighting. Abbreviations: ARIC, The Atherosclerosis Risk in Communities Study; CAGES, Cognitive Ageing Genetics in England and Scotland Consortium;^30^ GS, Generation Scotland.^78^ HRS, Health and Retirement Study.

This study provides further evidence that general cognitive function is heritable and under polygenic control. These findings are consistent with, and add considerably to those from the Cognitive Ageing in Genetics in England and Scotland consortium.^30^ The early deviation from the expected distribution in the QQ plot could be indicative of two outcomes; first, polygenic effects and, second, population stratification. All of the individual cohorts applied the required number of principal component analysis/multidimensional scaling components to their initial association analyses to adjust for stratification that may be present within the individual cohorts. None of the individual cohorts demonstrate both an early deviation and an inflated *λ*-value, which would indicate that stratification may be present (Supplementary Figure S2). Single genomic control was implemented in the meta-analysis. The proportion of phenotypic variance explained by all SNPs is an estimate of the lower bound of the narrow sense heritability. The estimates calculated from the ARIC and HRS cohorts suggest that 29% (s.e.=0.05) and 28% (s.e.=0.07), respectively, of the variation in general cognitive function can be attributed to common SNPs that are in linkage disequilibrium with causal variants in these cohorts. Whereas these estimates are lower than a previously-published estimate from the Cognitive Ageing in Genetics in England and Scotland consortium in a smaller sample (51% s.e.=0.11; *N*=3511),^30^ they are slightly higher than an estimate reported from a similar sample size in GS (21% s.e.=0.05; *N*=6648).^82^ To date, these are the only GCTA-based estimates of the general cognitive function phenotype in unrelated early-middle to older age individuals. The sample-size weighted mean of these estimates is 0.30 (Figure 4). It should be noted that these four estimates were not based on the same set of common SNPs, as only directly genotyped SNPs were included, and that the general cognitive function phenotypes in each cohort were constructed using a different set of cognitive tests. Therefore, the mean of 0.30 is likely to be an underestimate.

Whereas the combined sample size in the present report is a strength, the results observed here suggest that an even larger sample size is required in order to seek replication of the findings of the present study and to identify more genome-wide significant findings. For this reason, we chose to present a single discovery meta-analysis of the largest combined sample available. If general cognitive function is similar to other complex traits, the individual effects of common SNPs will be very small. From studies of other polygenic complex traits, it has been observed that the number of discovered variants is strongly correlated with experimental sample size.^31^ This predicted increase in detectable associations for complex traits, when sample sizes increase, has been observed in GWASs of both height—a study of 183 727 individuals reported 180 significant associations, of which >100 were novel loci compared with previous studies of fewer individuals (*N*<40 000)—and schizophrenia, in which a study of 21 246 cases and 38 072 controls reported 22 significant associations, of which 13 were novel loci compared with previous studies of fewer individuals (*N*<18 000 cases).^84, 85^ This is also demonstrated in the present study when compared with the previously published Cognitive Ageing in Genetics in England and Scotland consortium study (*N*=3511), which reported no genome-wide significant SNP associations with general cognitive function in older age.^30^

Phenotypic heterogeneity is a limitation of this study. Each cohort used a different set of cognitive tests to create the general cognitive function phenotype. We demonstrated using the Lothian Birth Cohort 1936 cohort that two general cognitive function phenotypes calculated in the same set of individuals but using a different battery of tests for the principal component analysis are highly (~0.8) but imperfectly correlated. This is consistent with the finding that general cognitive function phenotypes derived from different test batteries are highly correlated and measurement of this phenotype is not dependent on the use of specific cognitive tasks.^32, 33^ Given, especially, that a relatively small number of tests contributed to the general cognitive component in some of the cohorts, the heterogeneity of the phenotype will be a limitation on the discovery of genetic effects.

In order to dissect the regions of association identified in the present study further, deep sequencing of these regions in a larger sample would be required followed by in-depth functional genomics studies. This type of approach may elucidate the mechanisms underlying the observed associations and identify the causal variants within these associated genomic regions.

In conclusion, we report the largest meta-analysis of GWAS studies, to date, of fluid general cognitive function in middle and older age. We also report results showing that general cognitive function is heritable and highly polygenic, extending findings of previous studies involving general cognitive function in older individuals. We show genome-wide significant SNP-based associations within three genomic regions 6q16.1 (*MIR2113*), 14q12 (*AKAP6/NPAS3* region) and 19q13.32 (*TOMM40/APOE* region), and a genome-wide significant gene-based association with the *HMGN1* gene located on chromosome 21. The 19q13.32 region has long been associated with AD and more recently was associated with non-pathological cognitive aging;^28^ the 6q16.1, 14q12 and *HMGN1* regions contain genes associated with development of the brain,^78, 79^ neurological function,^71, 72, 79^ psychiatric disease^73, 74, 75, 76^ and educational attainment.^35^

## Notes

Bruce M Psaty serves on a DSMB for a clinical trial of a device funded by the manufacturer (Zoll LifeCor). All other authors declare no conflict of interest.
