# Supplementary material for: Genetic contributions to variation in general cognitive function: a meta-analysis of genome-wide association studies in the CHARGE consortium (N=53 949)
Source: Mol Psychiatry. 2015 Feb 3;20(2):183–92. doi: 10.1038/mp.2014.188 (PMC4356746; doi:10.1038/mp.2014.188)
Supplement: Supplementary Information 1 [file mp2014188x1.pdf]

Supplementary Information 1 – Cohorts, Phenotypes and Acknowledgements
This document contains supplementary material for: Davies et al. Genetic contributions to variation in general cognitive function: a meta-analysis of genome-wide association studies in the CHARGE Consortium (N = 53 949)

Section 1

1. *Cohort Descriptions* 2

2. *References* 15

Section 2

3. *Construction of General Cognitive Function Phenotype* 22

4. *References* 37

Section 3

5. *Acknowledgements* 42

Section 1: Cohort Descriptions

Aging Gene-Environment Susceptibility - Reykjavik Study (AGES)

The AGES-Reykjavik Study is a single center prospective cohort study based on the Reykjavik Study. The Reykjavik Study was initiated in 1967 by the Icelandic Heart Association to study cardiovascular disease and risk factors. The cohort included men and women born between 1907 and 1935 who lived in Reykjavik at the 1967 baseline examination. Re-examination of surviving members of the cohort was initiated in 2002 as part of the AGES-Reykjavik Study. The AGES-Reykjavik Study is designed to investigate aging using a multifaceted comprehensive approach that includes detailed measures of brain function and structure. All cohort members were European Caucasians. Briefly, as part of a comprehensive examination, all participants answered a questionnaire, underwent a clinical examination and had blood drawn[1]. All consenting participants were offered to take a neuropsychological test battery[2]. Among participants with genome-wide data, 2862 participants were available for the present analysis.

The Atherosclerosis Risk in Communities Study (ARIC)

The ARIC study is a prospective population-based study of atherosclerosis and clinical atherosclerotic diseases in 15 792 men and women, including 11 478 white participants, drawn from four United States communities (Suburban Minneapolis, Minnesota; Washington County, Maryland; Forsyth County, North Carolina; and Jackson, Mississippi). In the first three communities, the sample reflects the demographic composition of the community. In Jackson, only black residents were enrolled. Participants were between age 45 and 64 years at their baseline examination in 1987-1989 when blood was drawn for DNA extraction and participants consented to genetic testing[5]. A total of 15 020 participants, of which 10 898 were white, were genotyped at the Broad Institute, Boston, Massachusetts, and 9345 of the latter passed QC criteria for genotyping and were available for analysis after application of all exclusion criteria. Vascular risk factors and outcomes, including transient ischemic attack, and stroke, were determined in a standard fashion[6]. The second clinical examination of the ARIC Study cohort in 1990–1992 included the following three neuropsychological tests: the Delayed Word Recall Test, the Digit Symbol Substitution

Test, and the Word Fluency Test[7]. Among white participants with genome-wide data, 9173 participants were available for the present analysis.

The Austrian Stroke Prevention Study (ASPS)

The ASPS study is a single center prospective follow-up study on the effects of vascular risk factors on brain structure and function in the normal elderly population of the city of Graz, Austria. The procedure of recruitment and diagnostic work-up of study participants has been described previously[3,4]. A total of 2007 participants were randomly selected from the official community register stratified by gender and 5 year age groups. Individuals were excluded from the study if they had a history of neuropsychiatric disease, including previous stroke, transient ischemic attacks, and dementia, or an abnormal neurologic examination determined on the basis of a structured clinical interview and a physical and neurologic examination. During 2 study periods between September 1991 and March 1994 and between January 1999 and December 2003 an extended diagnostic work-up including neuropsychological testing was done in 1076 individuals aged 45 to 85 years randomly selected from the entire cohort: 509 from the first period and 567 from the second. In 1992, blood was drawn from all study participants for DNA extraction. All were European Caucasians. Genotyping was done at the Human Genotyping Facility, Genetic Laboratory Department of Internal Medicine, Erasmus MC, Rotterdam, The Netherlands, and successful in 996 participants. Of these, 765 participants were available for the present analysis.

The Berlin Aging Study II (BASE-II)

The total sample of the BASE-II study consists of 600 younger adults and 1600 older adults; for a detailed sample description, see Bertram *et al.*[8]. The cognitive data reported here were collected in an earlier study on neuromodulation in lifespan cognition[9,10]. Of the 1600 older adults included in BASE-II, 1414 had participated in that earlier study. Genotyping was performed using the Affymetrix Genome-Wide Human SNP Array 6.0 at the Max Planck Institute for Molecular Genetics, Berlin. After performing a standard quality control procedure, the effective sample was reduced to 1320 individuals (53.6%

female). At the time of cognitive testing, participants were 59 to 71 years of age (mean = 65.3; SD = 2.9). Recruitment of participants was based on advertisements in local newspapers and the public commuter transport system. All participants were Caucasian and all lived independently in the greater metropolitan area of Berlin, Germany. All participants reported normal or corrected vision, were right-handed, as indexed by the Edinburgh Handedness Index[11], had completed at least 8 years of education, and scored over 27 on the Mini-Mental Status Examination. No participant was on medications that may have affected cognition and none reported a history of head injuries, medical (e.g., heart attack), neurological (e.g., epilepsy), or psychiatric (e.g., depression) diseases.

The Betula Study (BETULA)

The examined Betula sample was part of a larger prospective cohort study on memory, health and aging[12,13]. All participants were recruited by random selection from the personal registry of the Umeå community. The Betula sub-sample used here consisted of 324 participants (221 females and 103 males) aged between 45 and 95 years (mean = 65.7; SD = 9.0). All participants were native speakers of Swedish. None of the participants had any history of severe neurological illness or events; all had normal or corrected to normal vision, and were in good general health. They were non-demented based on an extensive neuropsychological examination and clinical evaluation of data obtained at the test occasions and reviews of medical records starting from adulthood. The genotyping of the Betula sample was performed using the Illumina Human Omni express-Quad and 1S BeadChip, at the Life and Brain Centre, University of Bonn.

The Cardiovascular Health Study (CHS)

The CHS is a population-based observational cohort study of risk factors for vascular disease in adults 65 years or older conducted across 4 field centers in the United States: Sacramento County, California; Washington County, Maryland; Forsyth County, North Carolina; and Pittsburgh, Allegheny County, Pennsylvania[14]. The original predominantly white cohort of 5201 persons was recruited in 1989-1990

from a random sample of seniors on Medicare eligibility lists. An additional 687 African-Americans were enrolled in 1992-1993, for a total sample of 5888. Vascular risk factors and outcomes, including transient ischemic attack, stroke, cognition and dementia, were determined using standardized protocols[15,16,17]. DNA was extracted from blood samples drawn on all participants who consented to genetic testing at their baseline examination in 1989-90 or 1992-1993. In 2007-2008, genotyping was performed at the General Clinical Research Center's Phenotyping/Genotyping Laboratory at Cedars-Sinai on 3980 CHS participants who were free of cardiovascular disease at baseline and who had DNA available for genotyping. Because most other cohorts were predominantly white, the African American participants were excluded from this analysis to limit the potential for false positive associations due to population stratification. Among white participants, genotyping was attempted in 3397 participants and was successful in 3295 persons. Beginning in 1989/90 participants completed cognitive tests at 10 annual clinic visits. In addition, as part of the CHS Cognition Study[16,18], in 1997-99, participants were invited to undergo detailed neuropsychological assessment. Among participants with genome-wide data, 1517 participants were available for the present analysis.

Croatian Cohorts: Split and Korčula

The CROATIA study is part of a larger genetic epidemiology research program in Croatian island isolates, "10,001 Dalmatians". The genetic epidemiology research program in Croatian island isolates began in 1999[19], then expanded to study human genetic variation and effects of isolation and inbreeding[20,21], and finally entered the phase of focusing on diseases and gene mapping studies[22-24]. The CROATIA- Korčula study included 969 participants. The CROATIA-Split study included 535 persons collected in 2009 from the general (outbred) population Split. Split has a population of > 100 000 and is the second largest city in Croatia. Participants from the CROATIA-Korčula and CROATIA-Split studies were invited to undergo a neuropsychological examination. CROATIA-Korčula genotyping was performed at the Institute of Human Genetics, Helmholtz Zentrum München, Germany and CROATIA-Split genotyping was performed at AROS Applied Biotechnology, Aarhus, Denmark. Genotyping was

successful in 898 and 499 participants respectively for CROATIA-Korčula and CROATIA-Split. Among participants with genome-wide data, 327 and 304 individuals were available for the present analysis for CROATIA-Korčula and CROATIA-Split respectively.

Erasmus Rucphen Family (ERF)

The ERF study is a family-based cohort study in a genetically isolated population in the Netherlands[25,26], including 3000 participants. Participants are all descendents of a limited number of founders living in the 19th century. Extensive genealogical data is available for this population. The study protocol included venous puncture for DNA isolation and chemistry, cognitive evaluation, cardiovascular examination, eye assessments and body composition measurements. Genotyping was done at the Human Genotyping Facility, Genetic Laboratory Department of Internal Medicine, Erasmus MC, Rotterdam, and at the Genotyping Center of Leiden University, The Netherlands. In total, 2385 samples from the ERF Study were available with good quality genotyping data. Participants were invited to undergo a neuropsychological evaluation. Among participants with genome-wide data, a total of 1473 participants were available for the present analysis.

Framingham Heart Study (FHS)

The FHS is a three-generation, single-site, community-based, prospective cohort study that was initiated in 1948 to investigate risk factors for cardiovascular disease including stroke. It now comprises 3 generations of participants: the original cohort followed since 1948 (Original)[27]; their offspring and spouses of the offspring, followed since 1971 (Offspring)[28]; and children from the largest offspring families enrolled in 2000 (Gen 3)[29]. The Original cohort enrolled 5209 men and women who comprised two-thirds of the adult population then residing in Framingham, MA, USA. Survivors continue to receive biennial examinations. The Offspring cohort comprises 5124 persons (including 3514 biological offspring) who have been examined approximately once every 4 years. Participants in the first two generations were invited to undergo an initial neuropsychological test battery in 1999-2005[30].

Neuropsychological testing in Gen 3 only began in 2009 and is not included in these analyses. The population of Framingham was virtually entirely whites in 1948 when the Original cohort was recruited. Vascular risk factors and outcomes, including transient ischemic attack, stroke and dementia, were identified prospectively since 1948 through an ongoing system of FHS clinic and local hospital surveillance[31,32]. Participants had DNA extracted and provided consent for genotyping in the 1990s. Genotyping was performed at Affymetrix (Santa Clara, CA) through an NHLBI funded SNP-Health Association Resource (SHARe) project and successful in 4519 persons from the Original and Offspring cohorts. Of these 4519 persons 4116 were alive in 1999 when the neuropsychological study began. Of these, 2642 participants have undergone neuropsychological testing. After excluding participants with a neurological condition that might confound the cognitive assessment (e.g., brain tumor or severe head injury), 2426 participants were available for the present analysis.

Genetic Epidemiology Network of Arteriopathy (GENOA)

The Genetic Epidemiology Network of Arteriopathy (GENOA) study consists of hypertensive sibships that were recruited for linkage and association studies in order to identify genes that influence blood pressure and its target organ damage[33]. In the initial phase of the GENOA study (Phase I: 1996-2001) all members of sibships containing ≥ 2 individuals with essential hypertension clinically diagnosed before age 60 were invited to participate, including both hypertensive and normotensive siblings. In the second phase of the GENOA study (Phase II: 2000-2004), 1239 European American participants were successfully re-recruited to measure potential target organ damage due to hypertension. From 2001-2006, Phase II GENOA participants that had a sibling willing and eligible to participate underwent a neurocognitive testing battery to assess several domains of cognitive function including learning, memory, attention, concentration, and language (N=967). A total of 775 European American GENOA participants were included in this analysis.

Generation Scotland (GS)

Generation Scotland: the Scottish Family Health Study[34,35] is a family-structured, population-based cohort study recruited between 2006 and 2011. Regional sampling occurred in Glasgow, Tayside, Ayrshire, Arran, and North-East Scotland, yielding a total sample size of 24 084 with an age range between 18 and 100 and up to four generations per family. A full description of the cohort is provided elsewhere[34,35] and online at http://www.generationscotland.org/. A sub-sample of 10 000 participants were selected for genotyping, based on: Caucasian ethnicity, born in the UK (prioritising those born in Scotland), and full phenotype data. In the current analysis only unrelated subjects were included, leaving an analysis sample of 5487. Genotyping was performed at the Wellcome Trust Clinical Research Facility Genetics Core, Edinburgh[35]. The mean age of the sample was 58.7 years (SD = 8.0) and 3177 (58%) were female.

Helsinki Birth Cohort Study (HBCS)

The source cohort for the HBCS comprised 4130 women and 4630 men born as singletons at Helsinki University Central Hospital during 1934-44, who had birth and child welfare records and were living in Finland in 1971[37]. To achieve an intended sample size of 2000, a random subsample of 2902 subjects was invited to participate in the study; 2003 of them (1075 women and 928 men) were finally included[38]. Participants who could come to the examination center were invited to take a neuropsychological test battery. DNA was extracted from 1728 randomly selected participants of the HBCS. Genotyping was conducted at the Wellcome Trust Sanger Institute, Cambridge, UK.

Hunter Community Study (HCS)

The HCS is a community-based longitudinal investigation that was commenced in Australia in 2004–2005. The study aims to investigate retired and near-retired persons by sampling older Australians aged 55–85, randomly selected from electoral rolls in a regional area on the heavily populated east coast (New South Wales)[39]. All participants were invited to take the Audio Recorded Cognitive Screen (ARCS), an

instrument that uses an audio device to administer selected neuropsychological tests to unsupervised individuals[40]. Genotyping was conducted at the Hunter Medical Research Institute, Newcastle Australia.

Health and Retirement Study (HRS)

The Health and Retirement Study is a longitudinal survey of a representative sample of Americans over the age of 50 [41,42]. The current sample includes over 26 000 persons in 17 000 households. Respondents are interviewed every two years about income and wealth, health and use of health services, work and retirement, and family connections. A full description of the HRS is provided online at http://hrsonline.isr.umich.edu/index.php. DNA was extracted from saliva collected during a face-to-face interview in the respondents' homes. These data represent European American respondents who provided DNA samples and participated in the relevant cognitive tests. A total of 6123 HRS participants were included in this analysis.

Lothian Birth Cohorts 1921 (LBC1921) and 1936 (LBC1936)

The Lothian Birth Cohorts include surviving participants from the Scottish Mental Surveys of 1932 or 1947 (SMS1932 and SMS1947), having been born, respectively in 1921 (LBC1921) and 1936 (LBC1936)[43-45]. The LBC1921 cohort consists of 550 relatively healthy individuals, 316 females and 234 males, assessed on cognitive and medical traits at about 79 years of age. When tested, the sample had a mean age of 79.1 years (SD = 0.6). The LBC1936 consists of 1091 relatively healthy individuals assessed on cognitive and medical traits at about 70 years of age. At baseline the sample of 548 men and 543 women had a mean age 69.6 years (SD = 0.8). They were all Caucasian and almost all lived independently in the Lothian region (Edinburgh city and surrounding area) of Scotland. Genotyping was performed at the Wellcome Trust Clinical Research Facility, Edinburgh. Quality control measures were applied; 517 and 1005 participants remained for LBC1921 and LBC1936 respectively. Among participants with genome-wide data, 459 (LBC1921) and 934 (LBC1936) individuals were available for the present analysis.

The Rush Memory and Aging Project (MAP)

The MAP, started in 1997, enrolled older men and women from assisted living facilities in the Chicago area with no evidence on dementia at baseline[46]. Since October 1997, 1742 participants completed their baseline evaluation, of whom 1530 were non-Hispanic white people. The follow-up rate of survivors exceeds 90%. Participants agreed to annual clinical evaluations, and signed both an informed consent and an Anatomic Gift Act form donating their brains at time of death. A more detailed description of the MAP has been published previously[46]. Participants were invited to take a neuropsychological test battery. DNA was extracted from whole blood, lymphocytes, or frozen postmortem brain tissue. Genotyping was performed at the Broad Institute's Center for Genotyping and the Translational Genomics Research Institute[47]. Among participants with genome-wide data, 595 individuals were available for the present analysis.

Norwegian Cognitive NeuroGenetics Cohort (NCNG)

The Norwegian Cognitive NeuroGenetics sample (NCNG) comprises 393 healthy Norwegian individuals who have been submitted to a wide range of cognitive tests, broadly described in the protocol paper from Espeseth et al.[48] The 128 females and 265 males included in the study range from 45 to 79 years old (mean = 61.0; SD = 8.4). The NCNG participants were recruited through advertisements in local newspapers from Oslo and Bergen areas. They completed a cognitive testing battery, including six different tests to cover different cognitive domains. The genotyping of the NCNG sample was performed using the Illumina Human 610-Quad BeadChip, at the Life and Brain Centre, University of Bonn. More details about the genotyping and quality control may be accessed in Espeseth et al.[48]

The Older Australian Twins Study (OATS)

Participants were recruited from the Australian Twin Registry and also through a recruitment drive. At baseline, participants were aged 65 years and over. Inclusion criteria included an ability to consent, a co-twin who also consented to participate, completion of some education in English and residence in one of

the three Eastern states (Victoria, New South Wales, Queensland). Exclusion criteria included inadequate English to complete the assessment, current diagnosis of malignancy or other life-threatening medical illness and/or a current acute psychosis diagnosis. At baseline, there were 623 participants with a mean age of 70.8 years (SD = 5.5) and 65.2% of the sample were women. For further details see Sachdev et al.[49,50]. Genotyping was performed using the Illumina OnmiExpress array. After quality control checks there were 517 individuals remaining. The final sample size available for the present analysis was 442.

Orkney Complex Disease Study (ORCADES)

ORCADES is an ongoing, family-based, cross-sectional study that seeks to identify genetic factors influencing cardiovascular and other disease risk in the population isolate of the Orkney Isles in northern Scotland[51]. The North Isles of Orkney, the focus of this study, consist of a subgroup of ten inhabited islands with census populations varying from ~30 to ~600 people on each island. The first phase of data collection was carried out in Orkney between 2005 and 2007. Blood samples were provided by 1019 Orcadian volunteers who had at least one grandparent from the North Isles of Orkney. Participants were invited to take a neuropsychological test battery. Genome-wide genotyping was performed at the Helmholtz Centre in Munich on a subset of 719 participants. An additional 169 individuals were genotyped by Integragen in Paris. 430 individuals with both cognitive phenotypes and genome-wide genotyping were available for the present analysis.

PROspective Study of Pravastatin in the Elderly at Risk (PROSPER)

All data come from the PROspective Study of Pravastatin in the Elderly at Risk (PROSPER). A detailed description of the study has been published elsewhere[52-54]. PROSPER was a prospective multicenter randomized placebo-controlled trial to assess whether treatment with pravastatin diminishes the risk of major vascular events in the elderly. Between December 1997 and May 1999, we screened and enrolled subjects in Scotland (Glasgow), Ireland (Cork), and the Netherlands (Leiden). Men and women aged 70-82 years were recruited if they had pre-existing vascular disease or increased risk of such disease because

of smoking, hypertension, or diabetes. A total number of 5804 subjects were randomly assigned to pravastatin or placebo. A large number of prospective tests were performed including cognitive function measurements. A whole genome wide screening has been performed in the sequential PHASE project with the use of the Illumina 660K beadchip. DNA was available for genotyping in 5763 subjects. Genotyping was performed with the Illumina 660K beadchip, after QC 5244 subjects remained for analysis. For the current study, analyses were performed separately for each country.

The Religious Orders Study (ROS)

The ROS, started in 1994, enrolled Catholic priests, nuns, and brothers, from about 40 groups in 12 states[55]. Since January 1994, 1236 participants completed their baseline evaluation, of whom 1091 were non-Hispanic white. The follow-up rate of survivors exceeds 90%. Participants were free of known dementia at enrolment, agreed to annual clinical evaluations, and signed both an informed consent and an Anatomic Gift Act form donating their brains at time of death[55]. A more detailed description of the ROS has been published previously[55]. Participants were invited to take a neuropsychological test battery. DNA was extracted from whole blood, lymphocytes, or frozen post-mortem brain tissue. Genotyping was performed at the Broad Institute's Center for Genotyping and the Translational Genomics Research Institute[47]. Among participants with genome-wide data, 682 individuals were available for the present analysis.

Rotterdam Study (RSI, RSII and RSIII)

The Rotterdam Study is a population-based cohort study among inhabitants of a district of Rotterdam (Ommoord), The Netherlands, and aims to examine the determinants of disease and health in the elderly with a focus on neurogeriatric, cardiovascular, bone, and eye disease[56]. In 1990-1993, 7983 persons participated and were re-examined every 3 to 4 years (Rotterdam Study-I). In 1999, 3011 individuals who had become 55 years of age or moved into the study district since the start of the study were added to the cohort (Rotterdam Study-II), and in 2006 a further extension of the cohort was initiated in which 3932

subjects aged 45–54 years and living in the same district were included (Rotterdam Study-III)[57]. All participants had DNA extracted at their first visit. Genotyping was attempted in participants with high-quality extracted DNA. Genotyping was done at the Human Genotyping Facility, Genetic Laboratory Department of Internal Medicine, Erasmus MC, Rotterdam, The Netherlands. Participants underwent several neuropsychological tests at the baseline and follow-up examinations[58]. Participants are continuously monitored for major events, including dementia and stroke, by automated linkage of the general practitioners' records and hospital discharge files with the study database[59,60]. Among participants with genome-wide data, 5091 participants from the Rotterdam Study were available for the present analysis.

Sydney Memory and Ageing Study (Sydney MAS)

The Sydney Memory and Ageing Study is a longitudinal community-based study. The participants were randomly recruited from the compulsory electoral roll in Sydney and were aged 70-90 years. Exclusion criteria included limited English or a medical/psychological condition that would prevent them from completing assessments, dementia diagnosis, an age and education-adjusted MMSE score <24, psychotic symptoms or a diagnosis of schizophrenia/bipolar disorder, multiple sclerosis, motor neuron disease, developmental disability and/or a progressive malignancy. At baseline, there were 1037 participants with a mean age of 78.8 years (SD = 4.8) and 55.2% were female. Further details are given in Sachdev et al.[61] Genotyping was performed using the Affymetrix Human 6.0 array. Among participants with genome-wide data, 727 were available for the present analysis.

Tasmanian Study of Cognition and Gait (TASCOG)

TASCOG is a study of cerebrovascular mechanisms underlying gait, balance and cognition in a population-based sample of Tasmanian people aged at least 60 years. 395 individuals aged 60–86 years living in Southern Tasmania, Australia, were randomly selected from the electoral roll to participate in the study. Individuals were excluded if they lived in a nursing home, had a contraindication for magnetic

resonance scanning (MRI) or were unable to walk without a gait aid[62]. Participants were invited to take a neuropsychological test battery[63]. DNA was extracted from peripheral blood samples. Genotyping was performed at the Diamantina Institute and Institute of Molecular Biosciences, University of Queensland, Australia, for 370 participants. Among participants with genome-wide data 348 were available for the present analysis.

Three City Study (3C)

The 3C Study is a population-based, prospective study of the relationship between vascular factors and dementia[64]. It has been conducted in 3 French cities: Bordeaux (southwest France), Montpellier (south France), and Dijon (central eastern France). A sample of non-institutionalized subjects older than 65 years was randomly selected from the electoral rolls of each city. Between January 1999 and March 2001, 9686 subjects meeting the inclusion criteria agreed to participate. After recruitment, 392 subjects withdrew from the study. Thus, 9294 subjects were finally included in the study (2104 in Bordeaux, 4931 in Dijon, and 2259 in Montpellier). In this study, we excluded subjects with missing data (genetic or covariates) and first-degree relatives. The final sample size available for analysis was 5321.

References

1. Harris TB, Launer LJ, Eiriksdottir G, Kjartansson O, Jonsson PV, Sigurdsson G *et al*. Age, Gene/Environment Susceptibility-Reykjavik Study: multidisciplinary applied phenomics. *Am J Epidemiol* 2007; **165**: 1076-1087.
2. Saczynski JS, Jónsdóttir MK, Garcia ME, Jonsson PV, Peila R, Eiriksdottir G *et al*. Cognitive impairment: an increasingly important complication of type 2 diabetes: the age, gene/environment susceptibility--Reykjavik study. *Am J Epidemiol* 2008; **168**(10): 1132-1139.
3. Schmidt R, Fazekas F, Kapeller P, Schmidt H, Hartung HP. MRI white matter hyperintensities: three-year follow-up of the Austrian Stroke Prevention Study. *Neurology* 1999; **53**: 132-139.
4. Schmidt R, Lechner H, Fazekas F, Niederkorn K, Reinhart B, Grieshofer P *et al*. Assessment of cerebrovascular risk profiles in healthy persons: definition of research goals and the Austrian Stroke Prevention Study (ASPS). *Neuroepidemiology* 1994; **13**: 308-313.
5. The ARIC investigators. The Atherosclerosis Risk in Communities (ARIC) Study: design and objectives. *Am J Epidemiol* 1989; **129**(4): 687-702.
6. Rosamond WD, Folsom AR, Chambless LE, Wang CH, McGovern PG, Howard G *et al*. Stroke incidence and survival among middle-aged adults: 9-year follow-up of the Atherosclerosis Risk in Communities (ARIC) cohort. *Stroke* 1999; **30**: 736-743.
7. Blair CK, Folsom AR, Knopman DS, Bray MS, Mosley TH, Boerwinkle E. APOE genotype and cognitive decline in a middle-aged cohort. *Neurology* 2005; **64**(2): 268-276.
8. Bertram L, Böckenhoff A, Demuth I, Düzel S, Eckardt R, Li SC *et al*. Cohort Profile: The Berlin Aging Study II (BASE-II). *Int J Epidemiol* 2013; e-pub ahead of print14 March 2013; doi: 10.1093/ije/dyt018.
9. Li S-C, Papenberg G, Nagel IE, Preuschhof C, Schröder J, Nietfeld W *et al*. Aging magnifies the effects of dopamine transporter and D2 receptor genes on backward serial memory. *Neurobiol Aging* 2013; **34**: 358.e1-358.e10.

10. Papenberg G, Bäckman L, Nagel IE, Nietfeld W, Schröder J, Bertram L *et al*. Dopaminergic gene polymorphisms affect long-term forgetting in old age: further support for the magnification hypothesis. *J Cognitive Neurosci* 2013; **25** (4): 571-579.
11. Oldfield RC. The assessment and analysis of handedness: The Edinburgh inventory. *Neuropsychologia* 1971; **9**: 97–113.
12. Nilsson L-G, Backman L, Erngrund K, Nyberg L, Adolfsson R, Bucht G *et al*. The Betula prospective cohort study: memory, health and aging. *Aging Neuropsych Cogn* 1997; **4**: 1-32.
13. Nilsson L-G, Adolfsson R, Bäckman L, Frias CM de, Molander B, Nyberg L. Betula: A Prospective Cohort Study on Memory , Health and Aging Betula: A Prospective Cohort Study on Memory , Health and Aging. *Aging, Neuropsychol Cogn* 2004; **11**: 134–148.
14. Fried LP, Borhani NO, Enright P, Furberg CD, Gardin JM, Kronmal RA *et al*. The Cardiovascular Health Study: design and rationale. *Ann Epidemiol* 1991; **1**: 263-276.
15. Longstreth WT Jr, Bernick C, Fitzpatrick A, Cushman M, Knepper L, Lima J *et al*. Frequency and predictors of stroke death in 5,888 participants in the Cardiovascular Health Study. *Neurology* 2001; **56**: 368-375.
16. Lopez OL, Kuller LH, Fitzpatrick A, Ives D, Becker JT, Beauchamp N. Evaluation of dementia in the cardiovascular health cognition study. *Neuroepidemiology* 2003; **22**: 1-12.
17. Fitzpatrick AL, Kuller LH, Ives D, Lopez OL, Jagust W, Breitner J, Beauchamp N, Lyketsos C, Dulberg C. Incidence and Prevalence of Dementia in the Cardiovascular Health Study. *J Am Geriatr Soc* 2004; **52**:195-204.
18. Lopez OL, Becker JT, Jagust WJ, Fitzpatrick A, Carlson MC, DeKosky ST *et al*. Neuropsychological characteristics of mild cognitive impairment subgroups. *J Neurol Neurosurg Psychiatry* 2006; **77**: 159-165.
19. Rudan I, Campbell H, Rudan P. Genetic epidemiological studies of eastern Adriatic Island isolates, Croatia: objective and strategies. *Coll Antropol* 1999; **23**: 531-546.

20. Rudan I, Biloglav Z, Vorko-Jovic A, Kujundzic-Tiljak M, Stevanovic R, Ropac D *et al*. Effects of inbreeding, endogamy, genetic admixture, and outbreeding on human health: a (1001 Dalmatians) study. *Croat Med J* 2006; **47**: 601-610.
21. Campbell H, Carothers AD, Rudan I, Hayward C, Biloglav Z, Barac L *et al*. Effects of genome-wide heterozygosity on a range of biomedically relevant human quantitative traits. *Hum Mol Genet* 2007; **16**: 233-241.
22. Vitart V, Bencic G, Hayward C, Herman JS, Huffman J, Campbell S *et al*. Heritabilities of ocular biometrical traits in two croatian isolates with extended pedigrees. *Invest Ophthalmol Vis Sci* 2010; **51**: 737-743.
23. Polasek O, Marusic A, Rotim K, Hayward C, Vitart V, Huffman J *et al*. Genome-wide association study of anthropometric traits in Korcula Island, Croatia. *Croat Med J* 2009; **50**: 7-16.
24. Vitart V, Rudan I, Hayward C, Gray NK, Floyd J, Palmer CN *et al*. *SLC2A9* is a newly identified urate transporter influencing serum urate concentration, urate excretion and gout. *Nat Genet* 2008; **40**: 437-442.
25. Service S, DeYoung J, Karayiorgou M, Roos JL, Pretorious H, Bedoya G *et al*. Magnitude and distribution of linkage disequilibrium in population isolates and implications for genome-wide association studies. *Nat Genet* 2006; **38**: 556-560.
26. Aulchenko YS, Heutink P, Mackay I, Bertoli-Avella AM, Pullen J, Vaessen N *et al*. Linkage disequilibrium in young genetically isolated Dutch population. *Eur J Hum Genet* 2004; **12**: 527-534.
27. Dawber TR, Kannel WB. The Framingham study. An epidemiological approach to coronary heart disease. *Circulation* 1966; **34**: 553-555.
28. Feinleib M, Kannel WB, Garrison RJ, McNamara PM Castelli WP. The Framingham Offspring Study. Design and preliminary data. *Prev Med* 1975; **4**: 518-525.

29. Splansky GL, Corey D, Yang Q, Atwood LD, Cupples LA, Benjamin EJ *et al*. The Third Generation Cohort of the National Heart, Lung, and Blood Institute's Framingham Heart Study: design, recruitment, and initial examination. *Am J Epidemiol* 2007; **165**: 1328-1335.

30. Au R, Seshadri S, Wolf PA, Elias M, Sullivan L, Beiser A *et al*. New norms for a new generation: cognitive performance in the framingham offspring cohort. *Exp Aging Res* 2004; **30**: 333-358.

31. Carandang R, Seshadri S, Beiser A, Kelly-Hayes M, Kase CS, Kannel WB *et al*. Trends in incidence, lifetime risk, severity, and 30-day mortality of stroke over the past 50 years. *JAMA* 2006; **296**: 2939-2946.

32. Seshadri S, Beiser A, Kelly-Hayes M, Kase CS, Au R, Kannel WB *et al*. The lifetime risk of stroke: estimates from the Framingham Study. *Stroke* 2006; **37**: 345-350.

33. Daniels PR, Kardia SL, Hanis CL, Brown CA, Hutchinson R, Boerwinkle E *et al*. Genetic Epidemiology Network of Arteriopathy study. Familial Aggregation of Hypertension Treatment and Control in the Genetic Epidemiology Network of Arteriopathy (GENOA) Study. *Am J Med* 2004*;* **116**(10)**:** 676-681.

34. Smith BH, Campbell H, Blackwood D, Connell J, Connor M, Deary IJ, et al. Generation Scotland: the Scottish Family Health Study; a new resource for researching genes and heritability. *BMC Med Genet* 2006; **7**: 74.

35. Smith BH, Campbell A, Linksted P, Fitzpatrick B, Jackson C, Kerr SM, et al. Cohort profile: Generation Scotland: Scottish Family Health Study (GS:SFHS). The study, its participants and their potential for genetic research on health and illness. *Int J Epidemiol* 2012; **42**(3): 689-700.

36. Kerr SM, Campbell A, Murphy L, Hayward C, Jackson C, Wain LV, et al. Pedigree and genotyping quality analyses of over 10,000 DNA samples from the Generation Scotland: Scottish Family Health Study. *BMC Med Genet* 2013; **14**(1): 38.

37. Lahti J, Raikkonen K, Bruce S, Heinonen K, Pesonen AK, Rautanen A *et al*. Glucocorticoid receptor gene haplotype predicts increased risk of hospital admission for depressive disorders in the Helsinki birth cohort study. *J Psychiatr Res* 2011; **45**(9): 1160-1164.

38. Barker DJ, Osmond C, Forsen TJ, Kajantie E, Eriksson JG. Trajectories of growth among children who have coronary events as adults. *N Engl J Med* 2005; **353**(17): 1802-1809.

39. McEvoy M, Smith W, D'Este C, Duke J, Peel R, Schofield P *et al*. Cohort profile: The Hunter Community Study. *Int J Epidemiol* 2010; **39**(6): 1452-1463.

40. Schofield PW, Lee SJ, Lewin TJ, Lyall G, Moyle J, Atia J *et al*. The Audio Recorded Cognitive Screen (ARCS): a flexible hybrid cognitive test instrument. *J Neurol Neurosurg Psychiatry* 2010; **81**(6): 602-607.

41. Juster FT, Suzman R. An Overview of the Health and Retirement Study. *Journal of Human Resources* 1995; **30**:Suppl: S7-S56.

42. Sonnega A, Faul JD, Ofstedal MB, Langa KM, Phillips JWR, Weir DR. Cohort Profile: the Health and Retirement Study (HRS). *Int J Epidemiol* 2014; 43 (2): 576-585.

43. Deary IJ, Whiteman MC, Starr JM, Whalley LJ, Fox HC. The impact of childhood intelligence on later life: following up the Scottish Mental Surveys of 1932 and 1947. *J Pers Soc Psychol.*2004; **86**: 130-147.

44. Deary IJ, Gow AJ, Taylor MD, Corley J, Brett C, Wilson V *et al*. The Lothian Birth Cohort 1936: a study to examine influences on cognitive ageing from age 11 to age 70 and beyond. *BMC Geriatrics* 2007; **7**: 28.

45. Deary IJ, Gow AJ, Pattie A, Starr JM. Cohort profile: The Lothian Birth Cohorts of 1921 and 1936. *Int J Epidemiol* 2012; **41**(6): 1576-1584.

46. Bennett DA, Schneider JA, Buchman AS, Barnes LL, Boyle PA, Wilson RS. Overview and findings from the Rush Memory and Aging Project. *Cur Alzheimer Res* 2012; **9**: 648-665.

47. Chibnik LB, Shulman JM, Leurgans SE, Schneider JA, Wilson RS, Tran D *et al*. CR1 is associated with amyloid plaque burden and age-related cognitive decline. *Ann Neurol* 2011; **69**(3): 560-569.

48. Espeseth T, Christoforou A, Lundervold AJ, Steen VM, Le Hellard S, Reinvang I. Imaging and cognitive genetics: The Norwegian Cognitive NeuroGenetics sample. *Twin Res Hum Genet* 2012; **15**: 442-452.

49. Sachdev PS, Lammel A, Trollor JN, Lee T, Wright MJ, Ames D *et al*. A comprehensive neuropsychiatric study of elderly twins: the Older Australian Twins Study. *Twin Res Hum Genet* 2009; **12**(6): 573-582.

50. Sachdev PS, Lee T, Lammel A, Crawford J, Trollor JN, Wright MJ *et al*. Cognitive functioning in older twins: the Older Australian Twins Study. *Australas J Ageing* 2011; **30** Suppl 2: 17-23.

51. McQuillan R, Leutenegger AL, Abdel-Rahman R, Franklin CS, Pericic M, Barac-Lauc L *et al*. Runs of homozygosity in European populations. *Am J Hum Genet* 2008; **83**: 359-372.

52. Shepherd J, Blauw GJ, Murphy MB, Cobbe SM, Bollen EL, Buckley BM *et al*. The design of a prospective study of Pravastatin in the Elderly at Risk (PROSPER). PROSPER Study Group. PROspective Study of Pravastatin in the Elderly at Risk. *Am J Cardiol* 1999; **84**(10): 1192-1197.

53. Shepherd J, Blauw GJ, Murphy MB, Bollen EL, Buckley BM, Cobbe SM *et al*. PROspective Study of Pravastatin in the Elderly at Risk. Pravastatin in elderly individuals at risk of vascular disease (PROSPER): a randomised controlled trial. *Lancet* 2002; **360**(9346): 1623-1630.

54. Trompet S, Craen de AJM, Postmus I, Ford I, Sattar N, Caslake M *et al*. Replication of LDL GWAS hits in PROSPER/PHASE as validation for future (pharmaco)genetic analyses. *BMC Med Genet* 2011; **12**:131.

55. Bennett DA, Schneider JA, Arvanitakis Z, Wilson RS. Overview and findings from the Religious Orders Study. *Cur Alzheimer Res* 2012; **9**: 630-647.

56. Hofman A, Murad SD, Duijn CM van, Franco OH, Goedegebure A, Ikram MA *et al*. The Rotterdam Study: 2014 objectives and design update. *Eur J Epidemiol* 2013; **28**: 889-926.

57. Hofman A. Recent trends in cardiovascular epidemiology. *Eur J Epidemiol* 2009; **24**(12): 721-723.

58. Prins ND, van Dijk EJ, den Heijer T, Vermeer SE, Jolles J, Koudstaal PJ *et al*. Cerebral small-vessel disease and decline in information processing speed, executive function and memory. *Brain* 2005; **128**: 2034-2041.

59. Bots ML, Looman SJ, Koudstaal PJ, Hofman A, Hoes AW, Grobbee DE. Prevalence of stroke in the general population. The Rotterdam Study. *Stroke* 1996; **27**: 1499-1501.

60. Hollander M, Koudstaal PJ, Bots ML, Grobbee DE, Hofman A, Breteler MM. Incidence, risk, and case fatality of first ever stroke in the elderly population. The Rotterdam Study. *J Neurol Neurosurg Psychiatry* 2003; **74**: 317-321.

61. Sachdev PS, Brodaty H, Reppermund S, Kochan NA, Trollor JN, Draper B *et al*. The Sydney Memory and Ageing Study (MAS): methodology and baseline medical and neuropsychiatric characteristics of an elderly epidemiological non-demented cohort of Australians aged 70-90 years. *Int Psychogeriatr* 2010; **22**(8): 1248-1264.

62. Callisaya ML, Blizzard L, Schmidt MD, McGinley JL, Lord SR, Srikanth VK. A population-based study of sensorimotor factors affecting gait in older people. *Age Ageing* 2009*;* **38**: 290-295.

63. Martin K, Thomson R, Blizzard L, Wood A, Garry M, Srikanth V. Visuospatial ability and memory are associated with falls risk in older people: a population-based study. *Dement Geriatr Cogn Disord* 2009; **27**: 451-457.

64. The 3C Study Group. Vascular factors and risk of dementia. Design of the Three-City Study and baseline characteristics of the study population**.** *Neuroepidemiology* 2003; **22**: 316-325.

Section 2: Construction of General Cognitive Function Phenotype

3C

Scores on the following cognitive ability tests were used to create the fluid-type general cognitive function component: Trail Making Test B (TMTB), Benton Visual Retention Test (BVRT), and Delayed Recall (Five Words Memory Test). The tests, the method of application and key references have been described in detail elsewhere[1]. The listwise N was 5321. The Pearson correlations (*r*s) among the 3 tests ranged from -0.34 to 0.15 (mean 0.21). Principal components analysis was applied to these 3 tests. The first unrotated principal component (FUPC) accounted for 47.7% of the total test variance. Loadings on the FUPC were as follows: Delayed Recall = 0.41, BVRT = 0.65 and TMTB = -0.64.

AGES

Scores on the following cognitive ability tests were used to create the fluid-type general cognitive function component: Digit Backward Test[2], The Digit Symbol Substitution Test (DSST)[2], California Verbal Learning Test (CVLT)[3], The Figure Comparison Test[4], The Modified Stroop Test (trial 3)[5], The Cambridge Neuropsychological Test Automated Battery (CANTAB) Spatial Working Memory[6]. The tests, the method of application and key references have also been described in detail elsewhere[7]. Absolute value of the Pearson correlations (*r*s) among the 6 tests ranged from 0.21 to 0.77 (mean 0.39). Principal components analysis was applied to these 6 tests. The first unrotated principal component (FUPC) accounted for 50.1% of the total test variance. Loadings on the FUPC were as follows: Digits backward test = 0.64, DSST total correct cells = 0.87, CVLT 1-4 number of unique target words = 0.73, Figure comparison total correct in 60 sec = 0.83, STROOP trial 3 time sec = -0.62, CANTAB Spatial Working Memory total errors = -0.52.

ARIC

Scores on the following cognitive ability tests were used to create the fluid-type general cognitive function component: Delayed Word Recall Test (total number of words recalled), Digit Symbol Substitution Test (total number of correct symbols), Word Fluency Test (sum of letters F, A, and S). The tests, the method of application and key references have been described in detail elsewhere[8-10]. The listwise N was 10 534. The Pearson correlations (*r*s) among the 3 tests ranged from 0.24 to 0.43 (mean 0.34). Principal components analysis was applied to these 3 tests. The first unrotated principal component (FUPC) accounted for 56.1% of the total test variance. Loadings on the FUPC were as follows: Delayed Word Recall Test = 0.52, Digit Symbol Substitution Test = 0.63, Word Fluency Test = 0.58.

ASPS

Scores on the following cognitive ability tests were used to create the fluid-type general cognitive function component: Alterskonzentrations-Test (AKT; concentration test – time in s), Lern- und Gedächtnistest (LGT) (figural memory, total number of correct answers of two figural subtests), Lern- und Gedächtnistest (LGT) (verbal memory, total number of correct answers of three verbal subtests), Complex reaction time task (computerized task; reaction time in ms), Digit Span – backward (length of highest correctly repeated digit list), Purdue Pegboard Test (visuo-practical skills; total number of correct elements in most difficult condition [assembly]), Trail Making Test B (TMTB; time in s). The tests, the method of application and key references have been described in detail elsewhere[11-16]. The listwise N was 765. The Pearson correlations (*r*s) among the 7 tests ranged from 0.13 to 0.53 (mean 0.33). Principal components analysis was applied to these 7 tests. The first unrotated principal component (FUPC) accounted for 42.9% of the total test variance. Loadings on the FUPC were as follows: Alterskonzentrations-Test = -0.54 figural memory (LGT)= 0.65, verbal memory (LGT) = 0.73, Complex reaction time task = -0.54, Digit Span = 0.59, Purdue Pegboard Test = 0.72, TMTB = -0.77.

BASEII

Scores on the following cognitive ability tests were used to a create fluid-type general cognitive function component: Spatial Working Memory (SWM; accuracy for location memory at set size 4); Wisconsin Card Sorting Test (WCST; % correct), which is assumed to index cognitive control or executive functioning; Mental Rotation (MR; sum of correct items in 7 minutes; max. 40); and Identical Pictures (IP; sum of correct answers in 80 seconds; max. 46 items), which is a measure of perceptual speed. The tests, test instructions, and key references have been described in detail elsewhere (SWM and WCST[17]; IP[18]; MR: test was designed for this study with the original versions as models[19]). The listwise N was 1383. The Pearson correlations (*r*s) among the 4 tests ranged from 0.13 to 0.29 (mean 0.22). Principal components analysis was applied to these 4 tests. The first unrotated principal component (FUPC) accounted for 41.2% of the total test variance. Loadings on the FUPC were as follows: SWM = 0.68, WCST = 0.60, MR = 0.62, IP = 0.67.

BETULA

Scores on the following cognitive ability tests were used to create the fluid-type general cognitive function component: Free Recall of Subject Performed Tasks (SPT; Immediate recall of 16 verb-noun combinations that were enacted during encoding), Fluency A (Generation during 1 minute of words beginning with the letter A), Block design (from the Wechsler Adult Intelligence Scale), and Letter Digit (Letter Digit Substitution Test, 9 Letters/Digits; test time 1 min). The tests, the method of application and key references have been described in detail elsewhere[20,21]. The listwise N was 373. The Pearson correlations (*r*s) among the 4 tests ranged from 0.26 to 0.57 (mean 0.38). Principal components analysis was applied to these 4 tests. The first unrotated principal component (FUPC) accounted for 53.9% of the total test variance. Loadings on the FUPC were as follows: SPT Sum = 0.71, Fluency A = 0.60, Block design = 0.79, Letter Digit = 0.82.

CHS

Scores on the following cognitive ability tests were used to create the fluid-type general cognitive function component: Modified Mini-Mental Status Score (3MSE; total score up to 100), Digit Symbol Substitution Test (DSST; symbols correctly coded in 90 seconds), Benton Visual Retention Test (BVRT; number of designs of 10 correctly drawn after 10 second exposure with stimulus covered and immediate reproduction from memory tested), Trail Making Test A (TMTA; number of seconds to complete test), and Trail Making Test B (TMTB; number of seconds to complete test). The tests, the method of application and key references have been described in detail elsewhere[22,23]. The listwise N was 1519. The Pearson correlations (*r*s) among the 5 tests ranged from 0.25 to 0.53 (mean 0.41). Principal components analysis was applied to these 5 tests. The first unrotated principal component (FUPC) accounted for 53.1% of the total test variance. Loadings on the FUPC were as follows: 3MSE = 0.69, DSST = 0.79, BVRT = 0.67, TMTA = 0.67, and TMTB = 0.81.

CROATIA-KORČULA, CROATIA-SPLIT

Scores on the following cognitive ability tests were used to create the fluid-type general cognitive function component: Digit Symbol Coding (DSC; sum of correct coding in 2 minutes (KORCULA) and 1 minute (SPLIT)), Standard Progressive Matrices (SPM; sum of total correct answers in 30 minutes), Verbal Fluency (FAS; sum of letters: F, A, S), Audio-Verbal Learning Test (AVLT_8; delayed recall)[9,10,24]. For the KORCULA sample the listwise N was 451. The Pearson correlations (*r*s) among the 4 tests ranged from 0.37 to 0.75 (mean 0.49). Principal components analysis was applied to these 4 tests. The first unrotated principal component (FUPC) accounted for 62.3% of the total test variance. Loadings on the FUPC were as follows: DSC = 0.87, SPM = 0.86, FAS = 0.72, AVLT_8 = 0.69. For the SPLIT sample, the listwise N was 518. The Pearson correlations (rs) among the 4 tests ranged from 0.32 to 0.67 (mean 0.44). Principal components analysis was applied to these 4 tests. The first unrotated principal component (FUPC) accounted for 58.5% of the total test variance. Loadings on the FUPC were as follows: DSC = 0.84, SPM = 0.85, FAS = 0.64, AVLT_8 = 0.72.

ERF

Scores on the following cognitive ability tests were used to create the fluid-type general cognitive function component: Stroop 3 color-word card (time needed to complete), Trail Making Test B (TMTB; time needed to complete), Phonemic Fluency (sum of letters D,A,T), 15-word Auditory Verbal Learning Test (AVLT; sum of immediate (5 iterations) and delayed recall (once)), Wechsler Adult Intelligence Scale (WAIS) block design test (total correct). The tests, the method of application and key references have been described in detail elsewhere[25]. The listwise N was 1572. The absolute Pearson correlations (*r*s) among the N tests ranged from 0.27 to 0.49 (mean 0.40 when sign ignored). Principal components analysis was applied to these 5 tests. The first unrotated principal component (FUPC) accounted for 51.9% of the total test variance. Loadings on the FUPC were as follows: Stroop 3 = -0.74, TMTB= -0.78, Phonemic fluency = 0.73, AVLT-sum = 0.70, WAIS block design = 0.64.

FHS

Scores on the following cognitive ability tests were used to create the fluid-type general cognitive function component: Similarities, Trail Making Test B (TMTB), Logical memory (sum of immediate and delayed recall scores), Visual reproduction memory (sum of immediate and delayed recall scores), Paired associate learning (sum of immediate and delayed recall scores), and Hooper visual organization test. The tests, the method of application and key references have been described in detail elsewhere[26]. The Pearson correlations (*r*s) among the 6 tests ranged from -0.56 to 0.58 (mean 0.11). The magnitude of *r*s (absolute value) ranged from 0.36 to 0.58 (mean 0.44). Principal components analysis was applied to these 6 tests. The first unrotated principal component (FUPC) accounted for 53.6% of the total test variance. Loadings on the FUPC were as follows: Similarities = -0.71, TMTB = 0.77, Logical memory = -0.66, Visual reproduction memory = -0.78, Paired associate learning= -0.68, Hooper visual organization= -0.77. The listwise N was 2426.

GENOA

Scores on the following five cognitive ability tests were used to create the fluid-type general cognitive function component: Rey Auditory Verbal Learning Test (RAVLT; delayed recall score); Wechsler Adult Intelligence Scale-Revised (WAIS-R) Digit Symbol Substitution Test (DSST; total correct); Controlled Oral Word Association Test (COWAT; Sum of letters F, A, and S); Stroop Color-Word Test (total correct); Trail Making Test A (TMTA; time to complete). The tests, the method of application and key references have been described in detail elsewhere[9,10,27-32]. The listwise N was 775. The absolute value of the Pearson correlations (*r*s) among the 5 tests ranged from 0.21 to 0.57 (mean 0.40). Principal components analysis was applied to these 5 tests. The first unrotated principal component (FUPC) accounted for 52.4% of the total test variance. Loadings on the FUPC were as follows: RAVLT = 0.66, DSST = 0.86, COWAT = 0.59, Stroop = 0.78, TMTA = -0.70.

GS

Scores on the following cognitive ability tests were used to create the fluid-type general cognitive function component: Wechsler Digit Symbol Substitution Task, Wechsler Logical Memory Test, and Verbal Fluency (sum of letters C, F, and L). The tests, the method of application and key references have been described in detail elsewhere[33]. The listwise N was 5487. The Pearson correlations (*r*s) among the 3 tests ranged from 0.18 to 0.33 (mean 0.26). Principal components analysis was applied to these 3 tests. The first unrotated principal component (FUPC) accounted for 51.0% of the total test variance. Loadings on the FUPC were as follows: Wechsler Digit Symbol Substitution Task = 0.78, Wechsler Logical Memory Test = 0.65, Verbal Fluency = 0.71.

HBCS

Scores on the following cognitive ability tests were used to create the fluid-type general cognitive function component: Simple reaction time (mean of two tests) derived from the computerized test battery Cogstate® version 3.0.5 and four subtests derived from the CERAD test battery namely; Verbal fluency

(total number of animal names within 60 seconds), List learning (sum of recalling 10 words across three trials), Figure copy (copying four figures), and Visual retention (free recall of copied four figures). The tests, the method of application and key references have been described in detail elsewhere[34-36]. The listwise N was 790. The absolute Pearson correlations (*r*s) among the five tests ranged from 0.06 to 0.33 (mean 0.16). Principal components analysis was applied to these five tests. The first unrotated principal component (FUPC) accounted for 33.7% of the total test variance. Loadings on the FUPC were as follows: Simple reaction time = -0.46, Verbal fluency = 0.72, List learning = 0.72, Figure copy = 0.57 and Visual retention = 0.34.

HCS

Scores on the following cognitive ability tests were used to create the fluid-type general cognitive function component: verbal episodic memory (Newcastle Auditory Verbal Learning Test (NAVLT); total score), Letter fluency (total score), visuospatial functioning (clock drawing task: scored according to the system of Manos and Wu), Language (item naming), attention/executive function (Hunter Attentional Task A). The tests, the method of application and key references have been described in detail elsewhere[37]. The listwise N was 816. The Pearson correlations (*r*s) among the 5 tests ranged from 0.12 to 0.44 (mean 0.26). Principal components analysis was applied to these 5 tests. The first unrotated principal component (FUPC) accounted for 41.4% of the total test variance. Loadings on the FUPC were as follows: NAVLT = 0.51, Fluency = 0.52, visuospacial functioning = 0.39, Language = 0.36, attention/executive function = 0.42.

HRS

Scores on the following five cognitive ability tests were used to create the fluid-type general cognitive function component: Animal Fluency; Number Series; Delayed Recall; Serial 7's test; Backwards counting starting from 86. The tests, the method of application and key references have been described in detail elsewhere[38-40]. The listwise N was 6123. Pearson correlations (*r*s) among the 5 tests ranged from

0.09 to 0.34 (mean 0.20). Principal components analysis was applied to these 5 tests. The first unrotated principal component (FUPC) accounted for 36.9% of the total test variance. Loadings on the FUPC were as follows: Animal Fluency = 0.64, Number Series = 0.73, Delayed Recall = 0.61, Serial 7's = 0.63, Backward counting = 0.36.

LBC1921

Scores on the following cognitive ability tests were used to create the fluid-type general cognitive function component: Moray House Test (total score), Verbal Fluency (sum of letters C, F and L), Raven's Standard Progressive Matrices (sum of total correct answers in 20 minutes), Logical Memory (total of immediate and delayed recall). The tests, the method of application and key references have been described in detail elsewhere[41]. The listwise N was 505. The Pearson correlations (*r*s) among the 4 tests ranged from 0.17 to 0.71 (mean 0.40). Principal components analysis was applied to these 4 tests. The first unrotated principal component (FUPC) accounted for 55.9% of the total test variance. Loadings on the FUPC were as follows: Moray House Test = 0.90, Verbal Fluency = 0.56, Raven's Standard Progressive Matrices = 0.84, Logical Memory = 0.65.

LBC1936

Scores on the following cognitive ability tests were used to create the fluid-type general cognitive function component: Moray House Test (total score), Logical Memory (total score of immediate and delayed recall), Spatial Span (total score), Four choice reaction time (mean), Verbal Fluency (sum of letters C, F and L). The tests, the method of application and key references have been described in detail elsewhere[42]. The listwise N was 983. The Pearson correlations (*r*s) among the 5 tests ranged from 0.16 to 0.49 (mean 0.31). Principal components analysis was applied to these 5 tests. The first unrotated principal component (FUPC) accounted for 45.4% of the total test variance. Loadings on the FUPC were as follows: Moray House Test = 0.83, Logical Memory = 0.65, Spatial Span = 0.63, Four choice reaction time = -0.66, Verbal Fluency = 0.57.

MAP

Scores on the following cognitive ability tests were used to create the fluid-type general cognitive function component: Logical Memory (total score), Word List Memory (total score and recall), total of Digit Span Forward and Backward, Symbol Digit, Number Comparison, Line Orientation, Progressive Matrices, and Stroop word colour naming (number of colours read correctly in 30 seconds). The tests, the method of application and key references have been described in detail elsewhere[43]. The listwise N was 595. The Pearson correlations among the 8 tests ranged from 0.12 to 0.60 (mean 0.31). Principal components analysis was applied to these 8 tests. The first unrotated principal component (FUPC) accounted for 40.2% of the total test variance. Loadings on the FUPC were as follows: Logical Memory 0.63; Word List Memory and recall 0.72; Digit Span Forward and Backward 0.57; Symbol Digit 0.79; Number Comparison 0.64; Line Orientation 0.48; Progressive Matrices 0.47; Stroop word colour naming 0.72.

NCNG

Scores on the following cognitive ability tests were used to create the fluid-type general cognitive function component: Wechsler Abbreviated Scale of Intelligence Matrix reasoning (WASI MR), Delis-Kaplan Executive Function System Color-Word Interference Test (D-KEFS CWIT; first unrotated principal component (FUPC) of all four subtests), California Verbal Learning Test-II (CVLT-II; FUPC of Learning1-5, immediate recall, delayed recall), Cued Discrimination Test (CDT; Overall median reaction time from, CDT, a Posner-type cued spatial attention task). The tests, the method of application and key references have been described in detail elsewhere[44]. The listwise N was 393. The Pearson correlations (*r*s) among the 4 tests ranged from 0.16 to 0.42 (mean 0.26). Principal components analysis was applied to these 4 tests. The FUPC accounted for 44.3% of the total test variance. Loadings on the FUPC were as follows: WASI MR = 0.63, D-KEFS CWIT = 0.75, CVLT-II = 0.60, CDT = 0.67.

OATS

Scores on the following cognitive ability tests were used to create the fluid-type general cognitive function component: Digit Symbol Coding (total correct score in 120 seconds), Semantic Fluency (number of animals named in 1 minute), Controlled Oral Word Association Test (sum of the 3 letters, F, A, S), Logical Memory Delayed Recall (Wechsler Memory Scale-III, Story A, story elements recalled after 25-35 min delay), Benton Visual Retention Test Recognition (BVRT; 15 items, total recognition score), Block Design (Wechsler Adult Intelligence Scale-Revised, total score), Trail Making Test B (TMTB; time to completion), Rey Auditory Verbal Learning Test (RAVLT; Total words recalled over trials 1-5 plus words recalled after 30 min). The tests, the method of application and key references have been described in detail elsewhere[45,46]. The listwise N was 442. The Pearson correlations (*r*s) among the 8 tests ranged from 0.06 and 0.53 (mean 0.29). Principal components analysis was applied to these 8 tests. The first unrotated principal component (FUPC) accounted for 38.2% of the total test variance. Loadings on the FUPC were as follows: Digit Symbol = 0.70, Semantic Fluency (animals) = 0.63, Controlled Oral Word Association Test= 0.53; Logical Memory Delayed = 0.52, BVRT= 0.47; Block Design = 0.64; TMTB = 0.73, RAVLT = 0.66.

ORCADES

Scores on the following cognitive ability tests were used to create the fluid-type general cognitive function component: Digit Symbol Coding (sum of correct coding in 2 minutes); Verbal Fluency (sum of letters C, F and L); Logical Memory from Wechsler Memory Scale-III (paragraph immediate and delayed recall summed). The tests, the method of application and key references have been described in detail elsewhere [2,47,48,]. The listwise N was 1635. The Pearson correlations (*r*s) among the 3 tests ranged from 0.30 to 0.47 (mean 0.40). Principal components analysis was applied to these 3 tests. The first unrotated principal component (FUPC) accounted for 60.1% of the total test variance. Loadings on the FUPC were as follows: Digit Symbol Coding = 0.83, Verbal Fluency = 0.73, Logical Memory = 0.76.

PROSPER - Ireland

Scores on the following cognitive ability tests were used to create the fluid-type general cognitive function component: The STROOP colour coding test (third test), The Letter-Digit-Coding test, Picture Learning Test Immediate recall. The tests, the method of application and key references have been described in detail elsewhere[49]. The listwise N was 1538. The Pearson correlations (*r*s) among the three tests ranged from 0.31 to 0.47 (mean 0.37). Principal components analysis was applied to these three tests. The first unrotated principal component (FUPC) accounted for 57.9% of the total test variance. Loadings on the FUPC were as follows: Stroop Colour-coding test = 0.80, Letter-Digit-Coding test = 0.80, Picture Learning Test Immediate 3 = 0.68.

PROSPER – the Netherlands

Scores on the following cognitive ability tests were used to create the fluid-type general cognitive function component: The STROOP colour coding test (third test), The Letter-Digit-Coding test, Picture Learning Test Immediate recall. The tests, the method of application and key references have been described in detail elsewhere[49]. The listwise N was 739. The Pearson correlations (*r*s) among the three tests ranged from 0.31 to 0.51 (mean 0.39). Principal components analysis was applied to these three tests. The first unrotated principal component (FUPC) accounted for 59.3% of the total test variance. Loadings on the FUPC were as follows: Stroop Colour-coding test = 0.80, Letter-Digit-Coding test = 0.82, Picture Learning Test Immediate = 0.68.

PROSPER - Scotland

Scores on the following cognitive ability tests were used to create the fluid-type general cognitive function component: The STROOP colour coding test (third test), The Letter-Digit-Coding test, Picture Learning Test Immediate recall. The tests, the method of application and key references have been described in detail elsewhere[49]. The listwise N was 1803. The Pearson correlations (*r*s) among the three tests ranged from 0.27 to 0.52 (mean 0.37). Principal components analysis was applied to these three

tests. The first unrotated principal component (FUPC) accounted for 58.5% of the total test variance. Loadings on the FUPC were as follows: Stroop Colour-coding test = 0.80, Letter-Digit-Coding test = 0.83, Picture Learning Test Immediate = 0.65.

ROS

Scores on the following cognitive ability tests were used to create the fluid-type general cognitive function component: Logical Memory total score (sum of Logical memory Ia immediate recall and Logical memory IIa delayed recall), total score of Word List Memory and recall (sum of word list memory recall, trials 1 – 3, immediate and delay after 5 minutes), total of Digit Span Forward and Backward (sum of digit span forward and digit span backward), Symbol Digit Modalities test (total number of correct matches (90 seconds)), Number Comparison (sum of number of pairs correctly classified minus number incorrectly classified (90 seconds)), Judgment of Line Orientation (total number of correct pairs (out of 15)) and Standard Progressive Matrices (total number of correctly identified missing elements (out of 17)). The tests, the method of application and key references have been described in detail elsewhere[50]. The listwise N was 682. The Pearson correlations (*r*s) among the 7 tests ranged from 0.13 to 0.67 (mean 0.33). Principal components analysis was applied to these 7 tests. The first unrotated principal component (FUPC) accounted for 43.3% of the total test variance. Loadings on the FUPC were: Logical Memory = 0.62; Word List memory and recall = 0.67; Digit Span Forward and Backward 0.56; Symbol Digit Modalities test 0.81; Number Comparison 0.72; Judgment of Line Orientation 0.44; Standard Progressive Matrices 0.73.

RSI

Scores on the following cognitive ability tests were used to create the fluid-type general cognitive function component: 15-word learning test (sum of immediate (3 iterations) and delayed (once) recall), Stroop card 3 (time needed to complete the card), Verbal Fluency (number of animals named within one minute), Letter-digit Substitution task (LDST; number correctly coded). The tests, the method of

application and key references have been described in detail elsewhere[51]. The listwise N was 1923. The absolute Pearson correlations among the 4 tests ranged from 0.14 to 0.44 (mean 0.37). Principal components analysis was applied to these 4 tests. The first unrotated principal component (FUPC) accounted for 52.7% of the total test variance. Loadings on the FUPC were as follows: Stroop 3 = -0.71, 15-word learning = 0.69, Verbal Fluency = 0.71, LDST score = 0.79.

RSII

Scores on the following cognitive ability tests were used to create the fluid-type general cognitive function component: 15-word learning test (sum of immediate (3 iterations) and delayed (once) recall), Stroop card 3 (time needed to complete the card), Verbal Fluency (number of animals named within one minute), Letter-digit Substitution task (LDST; number correctly coded). The tests, the method of application and key references have been described in detail elsewhere[51]. The listwise N was 1639. The absolute Pearson correlations among the 4 tests ranged from 0.30 to 0.50 (mean 0.38). Principal components analysis was applied to these 4 tests. The first unrotated principal component (FUPC) accounted for 53.4% of the total test variance. Loadings on the FUPC were as follows: Stroop 3 = -0.76, 15-word learning = 0.68, Verbal Fluency = 0.68, LDST score = 0.80.

RSIII

Scores on the following cognitive ability tests were used to create the fluid-type general cognitive function component: 15-word learning test (sum of immediate (3 iterations) and delayed (once) recall), Stroop card 3 (time needed to complete the card), Verbal Fluency (number of animals named within one minute), Letter-digit Substitution task (LDST; number correctly coded). The tests, the method of application and key references have been described in detail elsewhere[51]. The listwise N was 3172. The absolute Pearson correlations among the 4 tests ranged from 0.31 to 0.52 (mean 0.39). Principal components analysis was applied to these 4 tests. The first unrotated principal component (FUPC)

accounted for 54.4% of the total test variance. Loadings on the FUPC were as follows: Stroop 3 = -0.78, 15-word learning = 0.67, Verbal Fluency = 0.70, LDST score = 0.80.

Sydney MAS

Individuals from non-English speaking backgrounds were excluded. Scores on the following cognitive function tests were used to create the fluid-type general cognitive ability component: Digit Symbol Coding (total correct score in 120 seconds), Semantic Fluency (number of animals named in 1 minute), Controlled Oral Word Association Test (sum of the 3 letters F, A, S), Logical Memory Delayed Recall (Wechsler Memory Scale-III, Story A, story elements recalled after 25-35 mins delay), Benton Visual Retention Test Recognition (BVRT; 15 items, total recognition score), Block Design (Wechsler Adult Intelligence Scale-Revised, total score), Trail Making Test Part B (TMTB; time to completion), Rey Auditory Verbal Learning Test (RAVLT; Total words recalled over trials 1-5 plus words recalled after 30 mins). The tests, the method of application and key references have been described in detail elsewhere[52]. The listwise N was 727. The Pearson correlations (*r*s) among the 8 tests ranged from 0.15 to 0.55 (mean 0.30). Principal components analysis was applied to these 8 tests. The first unrotated principal component (FUPC) accounted for 39.4% of the total test variance. Loadings on the FUPC were as follows: Digit Symbol = 0.75, Semantic Fluency = 0.65, Controlled Oral Word Association Test = 0.58, Logical Memory delayed = 0.51, BVRT = 0.57, Block Design = 0.63, TMTB = 0.72, RAVLT = 0.57.

TASCOG

Scores on the following cognitive ability tests were used to create the fluid-type general cognitive function component: Hopkins verbal memory test (immediate recall score + delayed recall score), Rey Complex Figure Copy, Digit Symbol (Wechsler Adult Intelligence Scale-III), Digit Span (Wechsler Adult Intelligence Scale-III), Victoria Stroop test (colour-word time), Verbal Fluency with Controlled Oral Word Association Test (COWAT). The tests, the method of application and key references have been described in detail elsewhere[53]. The listwise N was 311. The Pearson correlations (*r*s) among the N tests

ranged from 0.21 to 0.52 (mean 0.37). Principal components analysis was applied to these 6 tests. The first unrotated principal component (FUPC) accounted for 48.0 % of the total test variance. Loadings on the FUPC were as follows: Hopkins = 0.65; Rey Copy = 0.60; Digit Symbol = 0.82; Digit Span = 0.63; COWAT = 0.69; Stroop = -0.74.

References

1. The 3C Study Group. Vascular factors and risk of dementia. Design of the Three-City Study and baseline characteristics of the study population**.** *Neuroepidemiology* 2003; **22**: 316-325.
2. Wechsler D. *WAIS-IIIUK administration and scoring manual*. Psychological Corporation: London, 1998.
3. Delis DC, Kramer JH, Kaplan E, Ober BA. *California Verbal Learning Test: Adult Version Manual.* The Psychological Corporation: San Antonio, TX, 1987.
4. Salthouse T, Babcock R. Decomposing adult age differences in executive function. *Dev Psychol.* 1991; **27**: 763–776.
5. Spreen O, Strauss E. *A compendium of neuropsychological tests.* Oxford University Press: New York, 1991.
6. Sweeney JA, Kmiec JA, Kupfer DJ. Neuropsychologic impairments in bipolar and unipolar mood disorders on the CANTAB neurocognitive battery. *Biol Psychiatry* 2000; **48**(7): 674-684.
7. Saczynski JS, Jónsdóttir MK, Garcia ME, Jonsson PV, Peila R, Eiriksdottir G *et al*. Cognitive impairment: an increasingly important complication of type 2 diabetes: the age, gene/environment susceptibility--Reykjavik study. *Am J Epidemiol* 2008; **168**(10): 1132-1139.
8. Knopman DS, Ryberg S. A verbal memory test with high predictive accuracy for dementia of the Alzheimer type. *Arch Neurol 1989;* **46**: 141-145.
9. Wechsler D. *The Wechsler Adult Intelligence Scale – Revised*. Psychological Corp: New York, 1981.
10. Lezak M. *Neuropsychological Assessment 3rd Ed*. Oxford University Press: Oxford, 1995.
11. *Wiener Reaktionsgerät*. Grundprogramm. Mödling, Austria: Dr. Schuhfried Ges.m.b.H, 1991.
12. Tewes U. *Hamburg-wechsler intelligenztest für erwachsene*. Verlag Hans Huber: Bern, Switzerland, 1991.
13. Gatterer G. *Alters-konzentrations-test (akt)*. Dr CJ Hogrefe Verlag Psychologie: Göttingen, Germany, 1990.

14. Bäumler G. *Lern und gedächtnistest (lgt 3)*. Dr CJ Hogrefe Verlag Psychologie: Göttingen, Germany, 1974.

15. Tiffin J, Asher E. The purdue pegboard: Norms and studies of reliability and validity. *J Appl Psychol* 1948; **32**: 234-247.

16. *Army individual test battery. Manual of directions and scoring.* Adjutant General's office: Washington DC, United States War Department, 1944.

17. Nagel IE, Chicherio C, Li S, von Oertzen T, Sander T, Villringer A *et al*. Human aging magnifies genetic effects on executive functioning and working memory. *Front Hum Neurosci* 2008; **2**: 1. doi: 10.3389/neuro.09.001.2008.

18. Lindenberger U, Mayr U, Kliegl R. Speed and intelligence in old age. *Psychol Aging* 1993; **8**(2): 207-220.

19. Vandenberg SG, Kuse AR. Mental rotations, a group test of three-dimensional spatial visualization. *Percept Motor Skill* 1978; **47**: 599-604.

20. Nilsson L-G, Backman L, Erngrund K, Nyberg L, Adolfsson R, Bucht G *et al*. The Betula prospective cohort study: memory, health and aging. *Aging Neuropsych Cogn* 1997; **4**: 1-32.

21. Larsson M, Nilsson L-G, Olofsson JK, Nordin S. Demographic and Cognitive Predictors of Cued Odor Identification: Evidence from a Population-based Study. *Chemical Senses* 2004; **29**(6): 547-554.

22. Lopez OL, Becker JT, Jagust WJ, Fitzpatrick A, Carlson MC, DeKosky ST *et al*. Neuropsychological characteristics of mild cognitive impairment subgroups. *J Neurol Neurosurg Psychiatry* 2006; **77**: 159-165.

23. Fitzpatrick AL, Kuller LH, Ives D, Lopez OL, Jagust W, Breitner J *et al*. Incidence and Prevalence of Dementia in the Cardiovascular Health Study. *J Am Geriatr Soc* 2004; **52**:195-204.

24. Raven JC, Court JH, Raven J. Manual for Raven’s Progressive Matrices and Vocabulary Scales. H. K. Lewis: London, UK, 1977.

25. Liu F, Pardo LM, Schuur M, Sanchez-Juan P, Isaacs A, Sleegers K *et al*. The apolipoprotein E gene and its age specific effects on cognitive function. *Neurobiol Aging* 2010; **10**: 1831-1833.

26. Au R, Seshadri S, Wolf PA, Elias M, Sullivan L, Beiser A *et al*. New norms for a new generation: cognitive performance in the framingham offspring cohort. *Exp Aging Res* 2004; **30**: 333-358.

27. Rey A. *L'Examen Clinique en Psychologie.* Presses Universitaires de France, 1964.

28. Spreen O, Strauss E. *Memory. A Compendium of Neuropsychological Tests: Administration, Norms, and Commentary.* Oxford University Press: New York, 1998.

29. Wechsler D. *WAIS-R Manual.* Psychological Corporation: San Antonio, TX, 1981.

30. Benton AL. Neuropsychological Assessment. *Annu Rev Psychol* 1994; **45:** 1-23.

31. Stroop J. Studies of Inference in Serial Verbal Reactions. *J Exp Psycho* 1935; **18**: 643-662.

32. Golden CJ. *Stroop Color and Word Test: A Manual for Clinical and Experimental Uses.* Shoelting Company: Wood Dale, IL, 1978.

33. Smith BH, Campbell A, Linksted P, Fitzpatrick B, Jackson C, Kerr SM, et al. Cohort profile: Generation Scotland: Scottish Family Health Study (GS:SFHS). The study, its participants and their potential for genetic research on health and illness. *Int J Epidemiol* 2012; **42**(3): 689-700.

34. Collie A, Maruff P, Makdissi M, McCrory P, McStephen M, Darby D. CogSport: reliability and correlation with conventional cognitive tests used in postconcussion medical evaluations. *Clin. J. Sport Med* 2003; **13**: 28–32.

35. Paile-Hyvärinen M, Räikkönen K, Kajantie E, Darby D, Ylihärsilä H, Salonen, MK *et al*. Impact of glucose metabolism and birth size on cognitive performance in elderly subjects. *Diabetes Research and Clinical Practice* 2009; **83**: 379-386.

36. Heyman A, Fillenbaum G, Nash F. Consortium to Establish a Registry for Alzheimer's disease: The CERAD experience. *Neurology* 1997; **49**: Suppl 3.

37. Schofield PW, Lee SJ, Lewin TJ, Lyall G, Moyle J, Attia J *et al*. The Audio Recorded Cognitive Screen (ARCS): a flexible hybrid cognitive test instrument. *J Neurol Neurosur Ps* 2010; **81**(6): 602–607.

38. Crimmins EM, Kim JK, Langa KM, Weir DR. Assessment of cognition using surveys and neuropsychological assessment: the health and retirement study and the aging, demographics, and memory study. *J Gerontol B Psychol Sci Soc Sci* 2011; 66B(S1): i162–i171.
39. Ofstedal MB, Fisher GG, Herzog AR. *Documentation of Cognitive Functioning Measures in the Health and Retirement Study*. HRS Documentation Report DR-006. http://hrsonline.isr.umich.edu/sitedocs/userg/dr-006.pdf. Survey Research Center, Institute for Social Research, University of Michigan, Ann Arbor, MI. 2005.
40. Fisher GG, McArdle JJ, McCammon RJ, Weir DR. *New measures of cognitive functioning in the HRS: 2010 and beyond*. HRS Documentation Report DR-027. http://hrsonline.isr.umich.edu/sitedocs/userg/dr-027.pdf. Survey Research Center, Institute for Social Research, University of Michigan, Ann Arbor, MI. 2013.
41. Deary IJ, Whiteman MC, Starr JM, Whalley LJ, Fox HC. The impact of childhood intelligence on later life: following up the Scottish Mental Surveys of 1932 and 1947. *J. Pers. Soc. Psychol* 2004; **86**: 130-147.
42. Deary IJ, Gow AJ, Taylor MD, Corley J, Brett C, Wilson V *et al*. The Lothian Birth Cohort 1936: a study to examine influences on cognitive ageing from age 11 to age 70 and beyond. *BMC Geriatrics* 2007; **7**: 28.
43. Bennett DA, Schneider JA, Buchman AS, Mendes de Leon C, Bienias JL, Wilson RS. The Rush Memory and Aging Project: study design and baseline characteristics of the study cohort. *Neuroepidemiology* 2005; **25**: 163-175.
44. Espeseth T, Christoforou A, Lundervold AJ, Steen VM, Le Hellard S, Reinvang I. Imaging and cognitive genetics: The Norwegian Cognitive NeuroGenetics sample. *Twin Res Hum Genet* 2012; **15**: 442-452.
45. Sachdev PS, Lammel A, Trollor JN, Lee T, Wright MJ, Ames D *et al*. A comprehensive neuropsychiatric study of elderly twins: the Older Australian Twins Study. *Twin Res Hum Genet* 2009; **12**(6): 573-582.

46. Sachdev PS, Lee T, Lammel A, Crawford J, Trollor JN, Wright MJ *et al*. Cognitive functioning in older twins: the Older Australian Twins Study. *Australas J Ageing* 2011; **30** Suppl 2: 17-23.

47. Wechsler D. *WMS-IIIUK administration and scoring manual*. London: Psychological Corporation, 1998.

48. Lezak M. *Neuropsychological Assessment 4th Ed*. Oxford University Press: Oxford, 2004.

49. Houx PJ, Shepherd J, Blauw GJ, Murphy MB, Ford I, Bollen EL *et al*. Testing cognitive function in elderly populations: the PROSPER study. PROspective Study of Pravastatin in the Elderly at Risk. *J Neurol Neurosurg Psychiatry* 2002; **73**: 385-389.

50. Wilson RS, Beckett LA, Barnes LL, Schneider JA, Bach J, Evans DA *et al*. Individual differences in rates of change in cognitive abilities of older persons. *Psychology and Aging* 2002; **17**:179-193.

51. Prins ND, van Dijk EJ, den Heijer T, Vermeer SE, Jolles J, Koudstaal PJ *et al*. Cerebral small-vessel disease and decline in information processing speed, executive function and memory. *Brain* 2005; **128**: 2034-2041.

52. Sachdev PS, Brodaty H, Reppermund S, Kochan NA, Trollor JN, Draper B *et al*. The Sydney Memory and Ageing Study (MAS): methodology and baseline medical and neuropsychiatric characteristics of an elderly epidemiological non-demented cohort of Australians aged 70-90 years. *Int Psychogeriatr* 2010; **22**(8): 1248-1264.

53. Martin K, Thomson R, Blizzard L, Wood A, Garry M, Srikanth V. Visuospatial ability and memory are associated with falls risk in older people: a population-based study. *Dement Geriatr Cogn Disord* 2009; **27**: 451-457.

Section 3: Acknowledgements

3C

This work was made possible by the generous participation of the participants and their families. This work was supported by the National Foundation for Alzheimer's disease and related disorders, the Institut Pasteur de Lille, the Centre National de Génotypage, Inserm, FRC (fondation pour la recherche sur le cerveau) and Rotary. This work has been developed and supported by the LABEX (laboratory of excellence program investment for the future) DISTALZ grant (Development of Innovative Strategies for a Transdisciplinary approachto ALZheimer's disease). The Three-City Study was performed as part of a collaboration between the Institut National de la Santé et de la Recherche Médicale (Inserm), the Victor Segalen Bordeaux II University and Sanofi-Synthélabo. The Fondation pour la Recherche Médicale funded the preparation and initiation of the study. The 3C Study was also funded by the Caisse Nationale Maladie des Travailleurs Salariés, Direction Générale de la Santé, MGEN, Institut de la Longévité, Agence Française de Sécurité Sanitaire des Produits de Santé, the Aquitaine and Bourgogne Regional Councils, Fondation de France and the joint French Ministry of Research/INSERM "Cohortes et collections de données biologiques" programme. Lille Génopôle received an unconditional grant from Eisai.

AGES

The Age, Gene/Environment Susceptibility (AGES Reykjavik) Study was initiated to examine genetic susceptibility and gene/environment interaction as these contribute to phenotypes common in old age, and represents a continuation of the Reykjavik Study cohort begun in 1967. The Age, Gene/Environment Susceptibility Reykjavik Study has been funded by NIH contract N01-AG-12100, the NIA Intramural Research Program, Hjartavernd (the Icelandic Heart Association), and the Althingi (the Icelandic Parliament). The study is approved by the Icelandic National Bioethics Committee, (VSN: 00-063) and

the Data Protection Authority. The researchers are indebted to the participants for their willingness to participate in the study.

ARIC

The Atherosclerosis Risk in Communities Study is carried out as a collaborative study supported by National Heart, Lung, and Blood Institute contracts (HHSN268201100005C, HHSN268201100006C, HHSN268201100007C, HHSN268201100008C, HHSN268201100009C, HHSN268201100010C, HHSN268201100011C, and HHSN268201100012C), R01HL087641, R01HL59367, and R01HL086694; National Human Genome Research Institute contract U01HG004402; and National Institutes of Health contract HHSN268200625226C. The authors thank the staff and participants of the ARIC study for their important contributions. Infrastructure was partly supported by Grant Number UL1RR025005, a component of the National Institutes of Health and NIH Roadmap for Medical Research.

ASPS

The authors thank the staff and the participants for their valuable contributions. We thank Birgit Reinhart for her long-term administrative commitment, Elfi Hofer for the technical assistance at creating the DNA bank, Ing. Johann Semmler and Anita Harb for DNA sequencing and DNA analyses by TaqMan assays and Irmgard Poelzl for supervising the quality management processes after ISO9001 at the biobanking and DNA analyses. The research reported in this article was funded by the Austrian Science Fund (FWF) grant number P20545-P05 and P13180. The Medical University of Graz supports the databank of the ASPS.

BASE-II

BASE-II has been financed by the Max Planck Society and the Federal Ministry of Education and Research. For a summary of the design of the study, see Bertram, L., Böckenhoff, A., Demuth, I., Düzel, S., Eckardt, R., Li, S.-C., Lindenberger, U., Pawelec, G., Siedler, T., Wagner, G. G., & Steinhagen-

Thiessen, E. (2013). Cohort profile: The Berlin Aging Study II (BASE-II). Advance online publication. International Journal of Epidemiology.

BETULA

The Betula Study was supported by the Swedish Research Council to Lars-Göran Nilsson and Lars Nyberg (2001-6654, 2002-3794 and 2003-3883) and by a Wallenberg Scholar grant from the Knut and Alice Wallenberg Foundation to Lars Nyberg. Sudheer Giddaluru was supported by a grant from Helse Vest RHF to Stephanie Le Hellard (Grant 911554). We also thank the Centre for Advanced Study (CAS) at the Norwegian Academy of Science and Letters in Oslo for hosting collaborative projects and workshops between Norway, Sweden and Scotland in 2011-2012.

CHS

Cardiovascular Health Study: This CHS research was supported by NHLBI contracts HHSN268201200036C, HHSN268200800007C, N01HC55222, N01HC85079, N01HC85080, N01HC85081, N01HC85082, N01HC85083, N01HC85086; and NHLBI grants HL080295, HL087652, HL105756 with additional contribution from the National Institute of Neurological Disorders and Stroke (NINDS). Additional support was provided through AG023629 and R01AG15928 from the National Institute on Aging (NIA). A full list of principal CHS investigators and institutions can be found at CHS-NHLBI.org. The provision of genotyping data was supported in part by the National Center for Advancing Translational Sciences, CTSI grant UL1TR000124, and the National Institute of Diabetes and Digestive and Kidney Disease Diabetes Research Center (DRC) grant DK063491 to the Southern California Diabetes Endocrinology Research Center. The content is solely the responsibility of the authors and does not necessarily represent the official views of the National Institutes of Health.

CROATIA Korcula and Split

We would like to acknowledge the staff of several institutions in Croatia that supported the field work, including but not limited to The University of Split and Zagreb Medical Schools and the Croatian

Institute for Public Health. We would also like to acknowledge the invaluable contributions of the recruitment teams in Korcula and Split, the administrative teams in Croatia and Edinburgh and the people of Korcula and Split.

ERF

The ERF study as a part of EUROSPAN (European Special Populations Research Network) was supported by European Commission FP6 STRP grant number 018947 (LSHG-CT-2006-01947) and also received funding from the European Community's Seventh Framework Programme (FP7/2007-2013)/grant agreement HEALTH-F4-2007-201413 by the European Commission under the programme "Quality of Life and Management of the Living Resources" of 5th Framework Programme (no. QLG2-CT-2002-01254). This study was financially supported by the Netherlands Organization for Scientific Research (NWO), the Internationale Stichting Alzheimer Onderzoek (ISAO), the Hersenstichting Nederland (HSN) and the Centre for Medical Systems Biology (CMSB) in the framework of the Netherlands Genomics Initiative (NGI) and by the Russian Foundation for Basic Research (RFBR). We thank the participants from the Genetic Research in Isolated Populations, Erasmus Rucphen Family, who made this work possible. Also, we thank Petra Veraart for collecting all genealogical data.

FHS

We acknowledge the National Heart, Lung, and Blood Institute, which has funded the SHARe (SNP Health Association Resource) project. A portion of FHS computations were using the Linux Cluster for Genetic Analysis (LinGA-II) funded by the Robert Dawson Evans Endowment of the Department of Medicine at Boston University School of Medicine and Boston Medical Center. We also acknowledge additional support from the NHLBI (Contracts No. N01-HC-25195; No. N02-HL-6-4278, R01HL93029, U01HL 096917), the National Institute of Aging (AG08122, AG16495; AG033193) and the NINDS (NS17950). The content is solely the responsibility of the authors and does not necessarily represent the official views of the National Institute on Aging, the National Institute of Neurological Disorders and Stroke, the National Heart Lung and Blood Institute or the National Institutes of Health.

GENOA

Support for the Genetic Epidemiology Network of Arteriopathy (GENOA) was provided by the National Heart, Lung and Blood Institute (HL054464, HL054457, HL054481, HL071917, and HL87660) and the National Institute of Neurological Disorders and Stroke (NS041558) of the National Institutes of Health. Genotyping was performed at the Mayo Clinic (S.T.T., Mariza de Andrade, Julie Cunningham) and was made possible by the University of Texas Health Sciences Center (Eric Boerwinkle, Megan L. Grove-Gaona). We would also like to thank the families that participated in the GENOA study.

GS

Generation Scotland has received core funding from the Chief Scientist Office of the Scottish Government Health Directorates CZD/16/6 and the Scottish Funding Council HR03006. We are grateful to all the families who took part, the general practitioners and the Scottish School of Primary Care for their help in recruiting them, and the whole Generation Scotland team, which includes interviewers, computer and laboratory technicians, clerical workers, research scientists, volunteers, managers, receptionists, healthcare assistants and nurses. Genotyping of the GS:SFHS samples was carried out by the Genetics Core Laboratory at the Wellcome Trust Clinical Research Facility, Edinburgh, Scotland and was funded by the UK's Medical Research Council. REM and DJP undertook the work within the University of Edinburgh Centre for Cognitive Ageing and Cognitive Epidemiology, part of the cross council Lifelong Health and Wellbeing Initiative (MR/K026992/1). Funding from the BBSRC and Medical Research Council (MSRC) is gratefully acknowledged.

HBCS

We thank all study participants as well as everybody involved in the Helsinki Birth Cohort Study. Helsinki Birth Cohort Study has been supported by grants from the Academy of Finland, the Finnish Diabetes Research Society, Folkhälsan Research Foundation, Novo Nordisk Foundation, Finska

Läkaresällskapet, Signe and Ane Gyllenberg Foundation, University of Helsinki, Ministry of Education, Ahokas Foundation, Emil Aaltonen Foundation.

HCS

The authors would like to thank the men and women participating in the HCS as well as The University of Newcastle, Vincent Fairfax Family Foundation and The Hunter Medical Research Institute.

HRS

HRS is supported by the National Institute on Aging (NIA U01AG009740). The genotyping was funded separately by the National Institute on Aging (RC2 AG036495, RC4 AG039029). Our genotyping was conducted by the NIH Center for Inherited Disease Research (CIDR) at Johns Hopkins University. Genotyping quality control and final preparation of the data were performed by the Genetics Coordinating Center at the University of Washington.

LBC1921 and LBC1936

We thank the cohort participants and team members who contributed to these studies. Phenotype collection in the Lothian Birth Cohort 1921 was supported by the UK Biotechnology and Biological Sciences Research Council (BBSRC), The Royal Society and The Chief Scientist Office of the Scottish Government. Phenotype collection in the Lothian Birth Cohort 1936 was supported by Research Into Ageing (continues as part of Age UK The Disconnected Mind project). Genotyping of the cohorts was funded by the BBSRC. The work was undertaken by The University of Edinburgh Centre for Cognitive Ageing and Cognitive Epidemiology, part of the cross council Lifelong Health and Wellbeing Initiative (MR/K026992/1). Funding from the BBSRC and Medical Research Council (MRC) is gratefully acknowledged.

MAP and ROS

The MAP and ROS data in the analysis is supported by National Institute on Aging grants P30AG10161, R01AG17917, R01AG15819, R01AG30146, the Illinois Department of Public Health, and the Translational Genomics Research Institute.

NCNG

The NCNG study has been funded through the Research Council of Norway (including the FUGE program), the National Institutes of Health, the University of Oslo, the University of Bergen, the Bergen Research Foundation (BFS), Helse Vest, and the Western Norway Regional Health Authority, the KG Jebsen Centre for Psychosis Research, and Dr. Einar Martens Fund. We also thank the Centre for Advanced Study (CAS) at the Norwegian Academy of Science and Letters in Oslo for hosting collaborative projects and workshops between Norway, Sweden and Scotland in 2011-2012.

OATS

We thank the OATS participants and gratefully acknowledge the support and assistance of the OATS Research Team. This work was facilitated by access to the Australian Twin Registry, a national research resource supported by the NHMRC Enabling Grant 310667 and administered by the University of Melbourne. DNA was extracted by Genetic Repositories Australia, an Enabling Facility, supported by the NHMRC Grant 401184. OATS genotyping was partly funded by a CSIRO Flagship Collaboration Fund Grant. Genome-wide genotyping was performed by the Diamantina Institute, University of Queensland. OATS is supported by the National Health and Medical Research Council of Australia (NHMRC) Project Grant 1045325 and the NHMRC/ARC Strategic Award 401162. KAM is supported by the NHMRC Capacity Building Grant 568940 and an Alzheimer's Australia Dementia Research Foundation Postdoctoral Fellowship.

ORCADES

ORCADES was supported by the Chief Scientist Office of the Scottish Government, the Royal Society, the MRC Human Genetics Unit, Arthritis Research UK and the European Union framework program 6 EUROSPAN project (contract no. LSHG-CT-2006-018947). DNA extractions were performed at the Wellcome Trust Clinical Research Facility in Edinburgh. We would like to acknowledge the invaluable contributions of Lorraine Anderson and the research nurses in Orkney, the administrative team in Edinburgh and the people of Orkney.

PROSPER

The PROSPER study was supported by an investigator initiated grant obtained from Bristol-Myers Squibb. Prof. Dr. J. W. Jukema is an Established Clinical Investigator of the Netherlands Heart Foundation (grant 2001 D 032). Support for genotyping was provided by the seventh framework program of the European commission (grant 223004) and by the Netherlands Genomics Initiative (Netherlands Consortium for Healthy Aging grant 050-060-810).

RSI, RSII and RSIII

The generation and management of GWAS genotype data for the Rotterdam Study is supported by the Netherlands Organisation of Scientific Research NWO Investments (nr. 175.010.2005.011, 911-03-012). This study is funded by the Research Institute for Diseases in the Elderly (014-93-015; RIDE2), the Netherlands Genomics Initiative (NGI)/Netherlands Organisation for Scientific Research (NWO) project nr. 050-060-810. We thank Pascal Arp, Mila Jhamai, Marijn Verkerk, Lizbeth Herrera and Marjolein Peters for their help in creating the GWAS database, and Karol Estrada and Maksim V. Struchalin for their support in creation and analysis of imputed data. The Rotterdam Study is funded by Erasmus Medical Center and Erasmus University, Rotterdam, Netherlands Organization for the Health Research and Development (ZonMw), the Research Institute for Diseases in the Elderly (RIDE), the Ministry of Education, Culture and Science, the Ministry for Health, Welfare and Sports, the European Commission

(DG XII), and the Municipality of Rotterdam. The authors are grateful to the study participants, the staff from the Rotterdam Study and the participating general practitioners and pharmacists.

Sydney MAS

We thank the Sydney MAS participants and the Sydney MAS Research Team for their support and assistance. DNA was extracted by Genetic Repositories Australia, an Enabling Facility, supported by the NHMRC Grant 401184. Genome-wide genotyping was performed by the Ramaciotti Centre, University of New South Wales. Sydney MAS is supported by the National Health and Medical Research Council of Australia (NHMRC) Program Grants 350833 and 568969. KAM, SR, NAK are supported by the NHMRC Capacity Building Grant 568940. NAK is also supported by a NHMRC Early Career Fellowship and KAM by an Alzheimer's Australia Dementia Research Foundation Postdoctoral Fellowship.

TASCOG

National Health and Medical Research Council of Australia (NHMRC) Project Grants 403000, 491109; Heart Foundation/NHMRC Career Development Fellowship 606544 (VS); NHMRC Career development Fellowship (RS).

GTEx Project

The Genotype-Tissue Expression (GTEx) Project was supported by the Common Fund of the Office of the Director of the National Institutes of Health (commonfund.nih.gov/GTEx). Additional funds were provided by the NCI, NHGRI, NHLBI, NIDA, NIMH, and NINDS. Donors were enrolled at Biospecimen Source Sites funded by NCI\SAIC-Frederick, Inc. (SAIC-F) subcontracts to the National Disease Research Interchange (10XS170), Roswell Park Cancer Institute (10XS171), and Science Care, Inc. (X10S172). The Laboratory, Data Analysis, and Coordinating Center (LDACC) was funded through a contract (HHSN268201000029C) to the The Broad Institute, Inc. Biorepository operations were funded

through an SAIC-F subcontract to Van Andel Institute (10ST1035). Additional data repository and project management were provided by SAIC-F (HHSN261200800001E). The Brain Bank was supported by a supplement to University of Miami grant DA006227. Statistical Methods development grants were made to the University of Geneva (MH090941), the University of Chicago (MH090951 & MH090937), the University of North Carolina - Chapel Hill (MH090936) and to Harvard University (MH090948). The datasets used for the analyses described in this manuscript were obtained from the GTEx Portal on 24 September 2014.

International Genomics of Alzheimer's Project (IGAP)

We thank the IGAP for providing summary results data for these analyses. The investigators within IGAP contributed to the design and implementation of IGAP and/or provided data but did not participate in analysis or writing of this report. IGAP was made possible by the generous participation of the control subjects, the patients, and their families. The i–Select chips was funded by the French National Foundation on Alzheimer's disease and related disorders. EADI was supported by the LABEX (laboratory of excellence program investment for the future) DISTALZ grant, Inserm, Institut Pasteur de Lille, Université de Lille 2 and the Lille University Hospital. GERAD was supported by the Medical Research Council (Grant n° 503480), Alzheimer's Research UK (Grant n° 503176), the Wellcome Trust (Grant n° 082604/2/07/Z) and German Federal Ministry of Education and Research (BMBF): Competence Network Dementia (CND) grant n° 01GI0102, 01GI0711, 01GI0420. CHARGE was partly supported by the NIH/NIA grant R01 AG033193 and the NIA AG081220 and AGES contract N01–AG–12100, the NHLBI grant R01 HL105756, the Icelandic Heart Association, and the Erasmus Medical Center and Erasmus University. ADGC was supported by the NIH/NIA grants: U01 AG032984, U24 AG021886, U01 AG016976, and the Alzheimer's Association grant ADGC–10–196728.
