# Supplementary material for: Genetic contributions to variation in general cognitive function: a meta-analysis of genome-wide association studies in the CHARGE consortium (N=53 949)
Source: Mol Psychiatry. 2015 Feb 3;20(2):183–92. doi: 10.1038/mp.2014.188 (PMC4356746; doi:10.1038/mp.2014.188)

Supplementary Information 2 – Methods, Tables and Figures
This document contains supplementary material for: Davies et al. Genetic contributions to variation in general cognitive function: a meta-analysis of genome-wide association studies in the CHARGE Consortium (N = 53 949)

1. *Cohort Abbreviations* 3
2. *Supplementary Methods* 4
3. *References* *7*
4. *Supplementary Tables*

Supplementary Table S1 8

Supplementary Table S2 9

Supplementary Table S3 13

Supplementary Table S4 13

Supplementary Table S5 14

Supplementary Table S6A-B 15

Supplementary Table S7A-D 15

Supplementary Table S8 17

Supplementary Table S9 18

Supplementary Table S10 19

Supplementary Table S11 21

5. *Supplementary Figures*

Supplementary Figure S1 22

Supplementary Figure S2 28

Supplementary Figure S3 34

Supplementary Figure S4 37

Supplementary Figure S5 39

Supplementary Figure S6	41
Supplementary Figure S7	43
Supplementary Figure S8	44
Supplementary Figure S9	45

Cohort Abbreviations

AGES: Aging Gene-Environment Susceptibility - Reykjavik Study

ARIC: The Atherosclerosis Risk in Communities Study

ASPS: The Austrian Stroke Prevention Study

BASEII: Berlin Aging Study II

BETULA: The Betula Study

CHS: The Cardiovascular Health Study

ERF: Erasmus Rucphen Study

FHS: Framingham Heart Study

GENOA: Genetic Epidemiology Network of Arteriopathy

GS: Generation Scotland

HBCS: Helsinki Birth Cohort Study

HCS: Hunter Community Study

HRS: Health and Retirement Study

KORCULA: CROATIA-KORCULA

LBC1921: Lothian Birth Cohort 1921

LBC1936: Lothian Birth Cohort 1936

MAP: The Rush Memory and Aging Project

NCNG: Norwegian Cognitive NeuroGenetics Cohort

OATS: The Older Australian Twins Study

ORCADES: Orkney Complex Disease Study

PROSPER-Ireland: PROspective Study of Pravastatin in the Elderly at Risk- Ireland

PROSPER-Netherlands: PROspective Study of Pravastatin in the Elderly at Risk-Netherlands

PROSPER-Scotland: PROspective Study of Pravastatin in the Elderly at Risk-Scotland

ROS: The Religious Orders Study

RSI: The Rotterdam Study-I

RSII: The Rotterdam Study-II

RSIII: The Rotterdam Study-III

Sydney MAS: The Sydney Memory and Aging Study

SPLIT: CROATIA-SPLIT

TASCOG: Tasmanian Study of Cognition and Gait

3C: Three City Study

Supplementary Methods

Creation of polygenic profile score for Alzheimer's disease

International Genomics of Alzheimer's Project (IGAP) is a large two-stage study based upon genome-wide association studies (GWAS) on individuals of European ancestry. In stage 1, IGAP used genotyped and imputed data on 7,055,881 single nucleotide polymorphisms (SNPs) to meta-analyse four previously-published GWAS datasets consisting of 17,008 Alzheimer's disease cases and 37,154 controls (The European Alzheimer's disease Initiative – EADI the Alzheimer Disease Genetics Consortium – ADGC The Cohorts for Heart and Aging Research in Genomic Epidemiology consortium – CHARGE The Genetic and Environmental Risk in AD consortium – GERAD). In stage 2, 11,632 SNPs were genotyped and tested for association in an independent set of 8,572 Alzheimer's disease cases and 11,312 controls. Finally, a meta-analysis was performed combining results from stages 1 & 2. The results from the stage 1 meta-analysis were used to create a polygenic predictor which was used to predict general cognitive function and general fluid cognitive function phenotypes in the GS cohort.

Pathway and network analyses

INRICH

INRICH[1] was used to quantify the degree to which the most significant genomic regions identified in our GWAS overlapped with known biological pathways. Significant genomic intervals were identified using the PLINK [2] clumping procedure. The intervals were formed by selecting all SNPs with a *P*-value of less than 0.0005 as index SNPs. The region around each index SNP was then extended across a 250kb range and clumps were formed by including other SNPs if they were both nominally associated ($P < 0.05$) with general cognitive function and in moderate LD ($r^2 > 0.5$) with the index SNP according to the HapMap II CEU reference panel. Genomic intervals were included from subsequent analysis if they were within 20kb (5' or 3') of any known gene found in the UCSC human genome browser hg18 assembly. A total of 722 genomic intervals were found of which 434 were located within 20kb of a

known gene. Intervals which overlapped with each other were then merged, leaving 284 LD independent intervals to be analysed for enrichment.

Enrichment testing was carried out using the pathways found in Gene Ontology[3]. After filtering gene-sets by size, 1284 gene-sets of between 5 and 200 genes were included. The number of intervals that overlapped with genes found in each of the gene-sets of Gene Ontology was then counted. The significance of the overlap between the gene-sets and the intervals was assessed using 10 000 randomly assigned intervals, matched for gene density, SNP number and similar SNP density. Finally, a bootstrapping-based re-sampling method using 5000 permutations was used to correct the enrichment *P*-values of each gene-set for the number of sets tested.

Ingenuity Pathway analysis (IPA)

Ingenuity Pathway Analysis (IPA; Ingenuity Systems, www.ingenuity.com) was used to identify functions, pathways, and networks associated with general cognitive function. Gene symbols and *P*-values from the gene-based association analysis were uploaded to IPA and 17 013/17 715 were successfully mapped to corresponding objects in the Ingenuity Knowledge Base (IKB; September 2014). A filter criterion of *P*-value ≤ 0.01 was used to identify 581 molecules of interest (focus molecules) and the full list was used as a reference set for the IPA analysis. An IPA core analysis was performed on the dataset. Networks were constructed using the 581 focus molecules and associated bio-functions and canonical pathways were identified. Networks were given a score which is the –log(*P*-value) of Fisher's Exact Test, giving an indication of the fit of the network to the focus molecule set. IPA builds networks to a user specified target size; networks of 70 and 140 nodes were generated using direct interactions only. All other settings were left as default.

Functional annotation and gene expression

For three genomic regions, located on chromosomes 6, 14 and 19, functional annotation, gene expression and evidence of expression quantitative trait loci (eQTL) were explored using publicly available online resources. These three regions were identified from the genome-wide significant findings in the SNP-based meta-analysis ($P < 5 \times 10^{-8}$). The Genotype-Tissue Expression Portal (GTEx)

(http://www.gtexportal.org) was used to identify eQTLs associated with any SNP that had a *P*-value < 5 x 10^{-8} in the meta-analysis (13 SNPs). Functional annotation was investigated for the same 13 SNPs (*P* < 5 x 10^{-8}) described above using the Regulome DB database[4]. Regulome DB was used to identify regulatory DNA elements in non-coding and intergenic regions of the genome. Data describing differential expression of the top two genes from the VEGAS analyses, in six brain regions across the life course, were extracted from the Human Brain Transcriptome Project (hbatlas.org)[5].

References

1. Lee PH, O'Dushlaine C, Thomas B, Purcell SM. INRICH: interval-based enrichment analysis for genome-wide association studies. *Bioinformatics* 2012; **28**(13): 2.
2. Purcell S, Neale B, Todd-Brown K, Thomas L, Ferreira MA, Bender D *et al*. PLINK: a tool set for whole-genome association and population-based linkage analyses. *Am J Hum Genet* 2007; **81**(3): 559-575.
3. Ashburner M, Ball CA, Blake JA, Botstein D, Butler H, Cherry JM. *et al*. Gene Ontology: tool for the unification of biology. *Nat Genet* 2000; **25**(1): 4.
4. Boyle AP, Hong EL, Hariharan M, Cheng Y, Schaub MA, Kasowski M, *et al*. Annotation of functional variation in personal genomes using RegulomeDB. *Genome Res* 2012; **22**(9):1790-1797.
5. Kang HJ, Kawasawa YI, Cheng F, Zhu Y, Xu X, Li M, *et al*. Spatio-temporal transcriptome of the human brain. *Nature* 2011; **478**: 483–489.

Supplementary Table S1: Cohort descriptive statistics: The total number of participants (% female) and mean, minimum and maximum age per cohort are indicated.

Cohort	N (% female)	Mean age (sd)	Min age	Max age
AGES	2862 (58.0)	76.2 (5.3)	66	93
ARIC	9173 (53.2)	57.2 (5.7)	47	69
ASPS	765 (57.1)	65.2 (7.8)	47	83
BASEII	1383 (51.1)	65.3 (2.9)	59	71
BETULA	324 (68.2)	65.7 (9.0)	45	95
CHS	1517 (62.4)	80.4 (3.8)	74	97
ERF	1473 (54.3)	58.1 (8.4)	45	87
FHS	2426 (54.7)	64.0 (10.8)	45	96
GENOA	775 (59.7)	61.2 (8.8)	45	84
HBCS	790 (59.9)	68.1 (2.9)	61	77
HCS	816 (48.7)	65.9 (7.3)	55	86
HRS	6123 (59.4)	70.0 (9.6)	45	98
KORCULA	327 (65.1)	60.0 (8.6)	45	88
LBC1921	459 (59.9)	79.0 (0.6)	78	81
LBC1936	934 (49.7)	69.5 (0.8)	68	71
MAP	595 (73.4)	80.5 (6.8)	55	97
NCNG	393 (67.4)	61.0 (8.4)	45	78
OATS	442 (62.9)	70.5 (5.2)	65	89
ORCADES	430 (57.0)	62.7 (9.5)	45	85
PROSPER – Ireland	1538 (57.0)	75.3 (3.3)	69	83
PROSPER – Netherlands	739 (47.9)	74.9 (3.2)	70	83
PROSPER – Scotland	1803 (51.4)	75.2 (3.4)	70	83
ROS	682 (65.0)	75.1 (7.1)	61	102
RSI	1923 (57.5)	63.8 (5.8)	55	86
RSII	1318 (55.6)	67.4 (6.8)	58	98
RSIII	1850 (57.1)	55.9 (5.6)	46	89
SPLIT	304 (67.4)	58.2 (8.1)	46	85
Sydney MAS	727 (57.1)	78.4 (4.7)	70	91
TASCOG	290 (43.1)	71.5 (6.8)	61	86
3C	5281 (60.6)	73.9 (5.3)	65	95
GS	5487 (57.9)	58.7 (8)	45	92
**Total**	**53949 (57.3)**			

Supplementary Table S2: Cohort specific details of genotyping platforms, quality control and imputation algorithms. Abbreviations: PCA, Principal Component Analysis; MDS, Multidimensional Scaling; MAF, Minor Allele Frequency; SNP, Single Nucleotide Polymorphism; HWE, Hardy-Weinberg Equilibrium.

**Study**	**Genotyping Platform**	**Genotyping Centre**	**Calling Method**	**Sample call rate**	**SNP call rate**	**MAF**	**HWE *P*-value**	**Population stratification**	**Imputation software**	**Reference Panel**
**AGES**	Illumina Human CNV370 Duo BeadChip	NIA, NIH, USA	Illumina Bead Studio	< 97%	< 98%	< 0.01	$< 10^{-6}$	EIGENSTRAT	MACH	HapMap II CEU build 36 release 22
**ARIC**	Affymetrix GeneChip SNP Array 6.0	Broad Institute, USA	Birdseed	< 95%	<95%	< 0.01	$< 10^{-5}$	EIGENSTRAT	MACH (v1.0.16)	HapMap II CEU build 36
**ASPS**	Illumina Human610-Quad BeadChip	Erasmus MC, Rotterdam, NL	Illumina	< 98%	< 98%	< 0.01	$< 10^{-6}$	IBD matrix	MACH (v1.0.15)	HapMap II CEU build 36 release 22
**BASEII**	Affymetrix 6.0	ATLAS Biolabs, Inc.	Birdseed (v2)	< 95%	< 98%	< 0.01	$< 10^{-6}$	EIGENSOFT	IMPUTE2	1000 Genomes ALL phase 1 June 2011
**BETULA**	Illumina Omni Express and Omni 1S	Department of Genomics, Life & Brain Center, University of Bonn, Germany	Illumina GenomeStudio	< 97%	< 95%	< 0.01	$< 10^{-3}$	MDS	MACH and minimac	1000 genomes project – European populations data freeze 2011
**CHS**	Illumina Human CNV370 Duo BeadChip	Genotyping Laboratory at Cedars-Sinai, USA	Illumina Bead Studio	$\leq$ 95%	< 97%	< 0.01	$< 10^{-5}$	PCA	BIM-BAM	HapMap II CEU build 36
**ERF**	Illumina HumanHap 300K array, Illumina HumanHap 6k Beadchip, Illumina	Leiden University Medical Center, Leiden; Erasmus MC, Rotterdam, NL	Illumina BeadStudio; Affymetrix BRLMM	< 95%	< 98%	< 0.005	$< 10^{-6}$	NA	MACH	HapMap II CEU build 36

	Human 370K-Duo SNP array									
**FHS**	Affymetrix GeneChip Human Mapping 500K Array +50K Human Gene Focused Panel	Affymetrix (Santa Clara), USA	Affymetrix BRLMM	< 97%	< 97%	< 0.01	$< 10^{-6}$	EIGENSTRAT	MACH	HapMap II CEU build 36
**GENOA**	Affymetrix GeneChip SNP Array 6.0, Illumina Human 1M-Duo Beadchip	Mayo Clinic, Rochester (MN), USA	Birdseed, Illumina GenomeStudio	< 95%	< 95%	< 0.01	NA	PCA	MACH	HapMap II CEU build 36 release 22
**GS**	Illumina HumanOmniExpressExome-8 v1.0 DNA Analysis BeadChip	Wellcome Trust Clinical Research Facility (WTCRF) Edinburgh	GenomeStudio Analysis v2011.1	< 95%	< 98%	< 0.01	$< 10^{-3}$	MDS	MACH	HapMap II CEU build 36
**HBCS**	modified Illumina Infinium 610K Quad chip	Wellcome Trust Sanger Institute, Cambridge, UK	Illumina Genome Studio	< 95%	< 95%	< 0.01	$< 10^{-5}$	MDS	MACH	HapMap II CEU
**HRS**	Illumina Omni 2.5 Beadchip	Center for Inherited Disease Research, Johns Hopkins University, Baltimore, MD, USA	Illumina GenomeStudio GenTrain	< 98%	< 98%	< 0.01	$< 10^{-4}$	PCA	MACH (version 1.0.16)	HapMap II CEU build 36
**HCS**	Illumina Human610-Quad BeadChip	Hunter Medical Research Institute,	Genomestudio	< 95%	< 95%	< 0.01	$< 10^{-6}$	EIGENSTRAT	MACH (version 1.0.16)	HapMap II CEU build 36.1 release 24

		Newcastle Australia								
**Korcula**	Illumina HumanHap 370-Duo and HumanHap 370-Quad BeadChip	Helmholtz Centre, Munich	Illumina Bead Studio	< 97%	< 98%	< 0.01	< 10^{-6}	PCA	MACH	HapMap II CEU build 36
**LBC1921**	Ilumina 610-Quadv1	Wellcome Trust Clinical Research Facility (WTCRF) Edinburgh	Illumina GenomeStudio	< 95%	< 98%	< 0.01	< 10^{-3}	MDS	MACH	HapMap II CEU build 36 release 22
**LBC1936**	Ilumina 610-Quadv1	Wellcome Trust Clinical Research Facility (WTCRF) Edinburgh	Illumina GenomeStudio	< 95%	< 98%	< 0.01	< 10^{-3}	MDS	MACH	HapMap II CEU build 36 release 22
**MAP**	Affymetrix Genechip 6.0	Broad Institute, USA	Birdsuite, Broad Institute	< 95%	< 95%	< 0.01	< 10^{-6}	EIGENSTRAT	MACH (version 1.0.16a)	HapMap II CEU build 36 release 22
**NCNG**	Illumina 610-Quad	Department of Genomics, Life & Brain Center, University of Bonn, Germany	Illumina GenomeStudio	< 97%	< 95%	< 0.01	< 10^{-3}	MDS	MACH	HapMap II CEU build 36 release 22
**OATS**	Illumina OmniExpress	Diamantina Institute, University of Queensland	Illumina Genomestudio	<95%	$\leq$ 95%	<0.01	< 10^{-6}	EIGENSTRAT	MACH	HapMap II CEU build 36 release 22
**ORCADES**	Illumina HumanCNV 370 -Duo and HumanHap 300K	Helmholtz Centre, Munich, D and Integragen, Paris, F	Illumina GenCall	< 97%	< 98%	< 0.01	< 10^{-6}	MDS	MACH	HapMap II CEU build 36 release 22

**PROSPER**	Illumina Human 660-Quadv1	Erasmus MC, Rotterdam, NL	Illumina Bead Studio	< 97.5%	< 98%	< 0.01	< 10^{-6}	IBD matrix	MACH v1.0.16	HapMap II CEU build 36 release 22
**ROS**	Affymetrix Genechip 6.0	Broad Institute, USA	Birdsuite, Broad Institute	< 95%	< 95%	< 0.01	< 10^{-6}	EIGENSTRAT	MACH (version 1.0.16a)	HapMap II CEU build 36 release 22
**RSI**	Illumina HumanHap 550-Duo BeadChip	Erasmus MC, Rotterdam, NL	Birdsuite, Broad Institute	< 97.5%	< 98%	< 0.01	< 10^{-6}	IBD matrix	MACH (v1.0.15)	HapMap II CEU build 36 release 22
**RSII**	Illumina HumanHap 550-Duo BeadChip and Illumina Human 610 Quad BeadChip	Erasmus MC, Rotterdam, NL	Illumina Bead Studio	< 97.5%	< 98%	< 0.01	< 10^{-6}	IBD matrix	MACH (v1.0.16)	HapMap II CEU build 36 release 22
**RSIII**	Illumina Human 610 Quad BeadChip	Erasmus MC, Rotterdam, NL	Illumina Genome Studio	< 97.5%	< 98%	< 0.01	< 10^{-6}	IBD matrix	MACH (v1.0.16)	HapMap II CEU build 36 release 22
**Sydney MAS**	Affymetrix SNP 6.0	Ramaciotti Centre, UNSW	CRLMM (v1.10.0) in R	< 95%	< 95%	< 0.01	< 10^{-6}	EIGENSTRAT	MACH / minimac	HapMap II CEU build 36 release 22
**Split**	Illumina HumanHap 370-Quad BeadChip	AROS Applied Biotechnology Aarhus, DK	Illumina Bead Studio	< 97%	< 98%	< 0.01	< 10^{-6}	PCA	MACH	HapMap II CEU build 36
**TASCOG**	Illumina HumanCNV 370-Duo BeadChip	Diamantina Institute and Institute of Molecular Biosciences	Illumina GenCall	< 97%	< 97%	< 0.005	< 10^{-7}	EIGENSTRAT	MACH (v1.0.16)	HapMap II CEU build 36
**3C**	Illumina Human 610-Quad BeadChip	Centre National de Génotypage	Illumina BeadStudio	< 95%	< 98%	< 0.01	< 10^{-6}	EIGENSTRAT	IMPUTE (v2.2)	HapMap II CEU build 36.3 release 22

Supplementary Table S3: (to be included in SI as excel file). The estimated effect (beta), standard error (SE) and *P*-values are shown for SNPs which achieved a significance of $P < 1 \times 10^{-5}$ in the meta-analysis; bold type indicates genome-wide significance ($P < 5 \times 10^{-8}$). The results are ordered by significance of the association. Gene annotation from UCSC hg18. The direction was ordered as LBC1936, ARIC, 3C, AGES, ASPS, BASEII, BETULA, CHS, ERF, MAP, ROS, TASCOG, FHS, GENOA, HBCS, HCS, HRS, KORCULA, LBC1921, Sydney MAS, NCNG, OATS, ORCADES, PROSPER-Ireland, PROSPER-Netherlands, PROSPER-Scotland, RSI, RSII, RSIII, SPLIT, GS. 0 indicates that the effect size is zero; ? indicates that the SNP did not pass QC in that cohort.

Supplementary Table S4: (to be included in SI as excel file). Genes showing association with general cognitive function ($P < 1 \times 10^{-3}$) in the VEGAS gene-based analysis; bold type indicates genome-wide significance ($P < 2.8 \times 10^{-6}$). Abbreviations: SNP, single-nucleotide polymorphism; N SNPs, the number of SNPs in the gene (±50kb); Best-SNP, the most significant SNP within the gene; SNP-*P*value, the original association *P*-value for the most significant SNP within the gene.

Supplementary Table S5 The association of candidate genes, previously identified in the literature as associated with Alzheimer's disease (AD) or neuropathological features of AD and related dementias, using a gene-based test. Bold type indicates $P < 0.01$. Note that, because of linkage disequilibrium (LD), the *P*-values for *APOE* and *TOMM40* are not independent.

Chr	Gene	N SNPs	*P*-value
**19**	***TOMM40***	**61**	**2.6×10^{-4}**
**21**	***ABCG1***	**216**	**5.5×10^{-4}**
**19**	***APOE***	**64**	**1×10^{-3}**
**5**	***MEF2C***	**156**	**1.9×10^{-3}**
11	*PICALM*	188	0.04
14	*SLC24A4*	293	0.05
7	*EPHA1*	60	0.07
19	*ABCA7*	85	0.08
7	*ZCWPW1*	39	0.08
4	*GALNT7*	166	0.11
6	*HLA-DRB5*	41	0.11
22	*PHF21B*	308	0.12
6	*HLA-DRB1*	51	0.14
10	*FRMD4A*	1218	0.17
14	*FERMT2*	157	0.22
2	*BIN1*	207	0.24
19	*BLOC1S3*	47	0.39
19	*EXOC3L2*	50	0.41
19	*CD33*	134	0.43
18	*DSG2*	177	0.51
14	*RIN3*	287	0.62
8	*CLU*	142	0.69
11	*MS4A6A*	87	0.7
1	*CR1*	171	0.76
2	*INPP5D*	202	0.8
6	*CD2AP*	194	0.81
8	*PTK2B*	279	0.88
11	*SORL1*	190	0.96
20	*CASS4*	150	0.99

Supplementary Table S6A (to be included in SI as excel file) Top 100 SNP-based findings from the CHIC consortium GWAS of general cognitive function in childhood. Corresponding Beta, SE and P-values are shown from our CHARGE meta-analysis results. Bold type denotes those findings which are nominally significant ($P < 0.05$) in our CHARGE meta-analysis.

Supplementary Table S6B (to be included in SI as excel file) Top 20 gene-based findings from the CHIC consortium GWAS of general cognitive function in childhood. *P*-values are shown from the CHIC and our CHARGE gene-based analyses. Bold type denotes those findings which are nominally significant in our CHARGE gene-based analysis.

Supplementary Table S7A (to be included in SI as excel file) SNPs which reached either $P < 1 \times 10^{-6}$ in the discovery stage meta-analysis or genome-wide significance ($P < 5 \times 10^{-8}$) in the combined discovery+replication meta-analysis of years of education and college completion in the educational attainment GWAS (Rietveld et al. 2013). Corresponding effect sizes and *P*-values from our CHARGE meta-analysis are shown. Abbreviations: College, College completion; Edu years, years of educational attainment.

Supplementary Table S7B (to be included in SI as excel file) List of SNPs which are of suggestive significance ($P < 1 \times 10^{-5}$) in our general cognitive function meta-analysis and which are also nominally significant ($P < 0.05$) in the Rietveld et al GWAS of educational attainment. Abbreviations: College, College completion; Edu years, years of educational attainment. NA indicates that the SNP did not reach nominal significance.

Supplementary Table S7C (to be included in SI as excel file) Top 25 gene-based findings for years of educational attainment (Rietveld et al. 2013). *P*-values are shown from the educational attainment (years of education and college completion) and our CHARGE gene-based analyses. Bold type denotes those findings which are nominally significant ($P < 0.05$) in our CHARGE gene-based analysis. Abbreviations: College, College completion; Edu years, years of educational attainment.

Supplementary Table S7D (to be included in SI as excel file) Top 25 gene-based findings for college completion (Rietveld et al. 2013). *P*-values are shown from the educational attainment (years of education and college completion) and our CHARGE gene-based analyses. Bold type denotes those findings which are nominally significant ($P < 0.05$) in our CHARGE gene-based analysis. Abbreviations: College, College completion; Edu years, years of educational attainment.

Supplementary Table S8 Polygenic prediction results. The results from the meta-analysis (excluding Generation Scotland (GS)) were used to create a polygenic predictor which was used to predict cognitive phenotypes and health outcomes in the GS cohort. R^2 was calculated by taking the difference in R^2 between a null model that adjusted for age, sex, and 4 PCs, and the model that also included the polygenic prediction score. p-value corresponds to the prediction score term in the model. R^2 is presented as a percentage. Abbreviations gf, general fluid cognitive function; g, general cognitive function; MHVS, Mill Hill Vocabulary Scale; LM, Wechsler Logical Memory Test; VFT, Verbal Fluency Test; DST, Wechsler Digit Symbol Substitution Task; CVD, cardiovascular disease; HT, Hypertension; T2D, Type 2 diabetes.

	**P<0.01**		**P<0.05**		**P<0.10**		**P<0.50**		**P<=1**	
N SNPs	1149		5289		10176		47322		93002	
	**R^2**	**P**	**R^2**	**p**	**R^2**	**P**	**R^2**	**p**	**R^2**	**p**
Cognitive traits										
gf	0.038	0.126	0.248	9.38×10^{-5}	0.461	9.87×10^{-8}	0.962	1.23×10^{-14}	0.942	2.32×10^{-14}
g	0.099	0.018	0.435	6.38×10^{-7}	0.775	2.86×10^{-11}	1.270	1.5×10^{-17}	1.241	3.53×10^{-17}
MHVS	0.184	0.001	0.495	1.3×10^{-7}	0.831	7.44×10^{-12}	0.935	3.77×10^{-13}	0.908	8.02×10^{-13}
LM	0.011	0.418	0.098	1.76×10^{-2}	0.326	1.5×10^{-5}	0.380	2.98×10^{-6}	0.352	7×10^{-6}
VFT	0.002	0.722	0.078	3.65×10^{-2}	0.104	1.6×10^{-2}	0.322	2.23×10^{-5}	0.314	2.8×10^{-5}
DST	0.070	0.028	0.209	1.41×10^{-4}	0.334	1.5×10^{-6}	0.805	7.32×10^{-14}	0.811	6×10^{-14}
Medical conditions										
CVD	0.014	0.587	0.207	0.038	0.108	0.135	0.001	0.917	0.006	0.727
HT	0.017	0.422	0.002	0.811	0.035	0.253	0.003	0.758	0.002	0.770
T2D	0.016	0.605	0.042	0.399	0.147	0.115	0.212	0.058	0.201	0.066

Supplementary Table S9 Polygenic prediction results for educational attainment and Alzheimer's disease. The results from published meta-analyses of educational attainment and Alzheimer's disease were used to create a polygenic predictor which was used to predict cognitive phenotypes in the GS cohort. R^2 was calculated by taking the difference in R^2 between a null model that adjusted for age, sex, and 4 PCs, and the model that also included the polygenic prediction score. *P*-value corresponds to the prediction score term in the model. R^2 is presented as a percentage. Abbreviations: gf, general fluid cognitive function; g, general cognitive function; Edu college, college completion; Edu years, years of educational attainment; AD Alzheimer's disease.

	**Threshold**	**0.01**			**0.05**			**0.1**			**0.5**			**1**		
		**R^2**	**P**	**N**	**R^2**	**P**	**N**	**R^2**	**P**	**N**	**R^2**	**P**	**N**	**R^2**	**P**	**N**
Edu college	gf	0.11	0.008	1126	0.21	3.28×10^{-4}	4843	0.29	2.74×10^{-5}	9167	0.37	2.08×10^{-6}	40239	0.34	4.13×10^{-6}	76943
	g	0.15	0.004		0.35	7.47×10^{-6}		0.39	2.4×10^{-6}		0.54	2.78×10^{-8}		0.52	5.19×10^{-8}	
Edu years	gf	0.05	0.080	1164	0.09	0.020	4859	0.11	0.008	9114	0.17	1.41×10^{-3}	39708	0.19	5.98×10^{-4}	75767
	g	0.08	0.028		0.16	0.002		0.17	0.002		0.32	2.24×10^{-5}		0.37	4.97×10^{-6}	
AD	gf	0.00	0.854	1088	0.01	0.412	4984	0.04	0.113	9547	0.11	0.01	44149	0.11	0.008	85724
	g	0.00	0.718		0.01	0.568		0.06	0.075		0.17	0.002		0.19	0.001	

Supplementary Table S10 Gene-sets reaching nominal significance (p<0.05) before correction for multiple comparisons are shown below. Number of genes total relates to the total number of genes in each gene-set according to Gene Ontology (GO), whereas number of genes significant details the number of genes from each gene-set that overlapped with the genomic intervals tested here. The enrichment P-value describes the probability of each gene-set overlapping with the tested genomic intervals. Corrected P-values are the enrichment P-values corrected for the total number (1,284) of gene-sets examined.

Gene-set		Number of genes		P-value	
GO term	Name	Total	Significant	Enrichment	Corrected
GO:0005001	transmembrane receptor protein tyrosine phosphatase activity	18	8	0.0002	0.3347
GO:0006470	protein amino acid dephosphorylation	117	14	0.0004	0.4515
GO:0004725	protein tyrosine phosphatase activity	80	10	0.0008	0.6224
GO:0007169	transmembrane receptor protein tyrosine kinase signaling pathway	69	12	0.0008	0.6224
GO:0008159	positive transcription elongation factor activity	6	3	0.0018	0.8422
GO:0004714	transmembrane receptor protein tyrosine kinase activity	34	8	0.0020	0.8631
GO:0007185	transmembrane receptor protein tyrosine phosphatase signaling pathway	7	4	0.0030	0.9321
GO:0045078	positive regulation of interferon-gamma biosynthetic process	11	3	0.0034	0.9540
GO:0055037	recycling endosome	19	4	0.0034	0.9540
GO:0030426	growth cone	56	7	0.0048	0.9860
GO:0048786	presynaptic active zone	6	3	0.0074	0.9990
GO:0003730	mRNA 3'-UTR binding	17	3	0.0098	0.9990
GO:0034375	high-density lipoprotein particle remodeling	13	3	0.0104	0.9990
GO:0007156	homophilic cell adhesion	97	11	0.0106	0.9990
GO:0007605	sensory perception of sound	87	10	0.0116	0.9990
GO:0016529	sarcoplasmic reticulum	23	5	0.0124	0.9990
GO:0007040	lysosome organization	20	3	0.0130	0.9990
GO:0034704	calcium channel complex	8	3	0.0146	0.9990
GO:0042462	eye photoreceptor cell development	12	3	0.0146	0.9990
GO:0030336	negative regulation of cell migration	36	5	0.0156	1.0000
GO:0016455	RNA polymerase II transcription mediator activity	28	4	0.0158	1.0000
GO:0048168	regulation of neuronal synaptic plasticity	13	3	0.0168	1.0000
GO:0016525	negative regulation of angiogenesis	29	4	0.0174	1.0000
GO:0043234	protein complex	151	12	0.0190	1.0000

GO:0005769	early endosome	87	7	0.0214	1.0000
GO:0043691	reverse cholesterol transport	16	3	0.0214	1.0000
GO:0016592	mediator complex	31	4	0.0230	1.0000
GO:0042311	vasodilation	11	3	0.0246	1.0000
GO:0006898	receptor-mediated endocytosis	39	5	0.0282	1.0000
GO:0030425	dendrite	122	11	0.0284	1.0000
GO:0032313	regulation of Rab GTPase activity	48	5	0.0284	1.0000
GO:0042472	inner ear morphogenesis	43	4	0.0306	1.0000
GO:0016485	protein processing	19	3	0.0310	1.0000
GO:0008013	beta-catenin binding	40	5	0.0316	1.0000
GO:0006641	triglyceride metabolic process	26	3	0.0340	1.0000
GO:0004888	transmembrane receptor activity	114	8	0.0374	1.0000
GO:0005694	chromosome	141	7	0.0404	1.0000
GO:0005882	intermediate filament	85	4	0.0410	1.0000
GO:0060070	Wnt receptor signaling pathway through beta-catenin	23	3	0.0432	1.0000
GO:0071339	MLL1 complex	26	3	0.0432	1.0000
GO:0017046	peptide hormone binding	21	3	0.0470	1.0000
GO:0014069	postsynaptic density	77	9	0.0492	1.0000

Supplementary Table S11 Functional annotation of the top SNPs from the meta-analysis. All information contained in this table was extracted from the Regulome DB database (http://regulome.stanford.edu/index). All cis-eQTL information presented here was based on analysis of lymphoblastoid cell lines. All data shown regarding regulatory features was restricted to CNS relevant normal tissues and cell lines.

	**rs10119**	**rs10457441**	**rs1872841**	**rs9375195**	**rs12202969**	**rs9401634**	**rs9375225**
**cis-eQTL**	n	n	n	n	n	n	n
**Position weight matrix**	y	n	y	y	y	y	y
**Transcription factor binding site**	n	n	n	n	n	n	n
**Histone modifications**	y	y	y	y	y	y	y
**DNase hypersensitive sites**	y	y	n	n	n	n	n
**FAIRE sites**	n	n	n	n	n	n	n

Supplementary Figure S1: Manhattan plots of the cohorts.

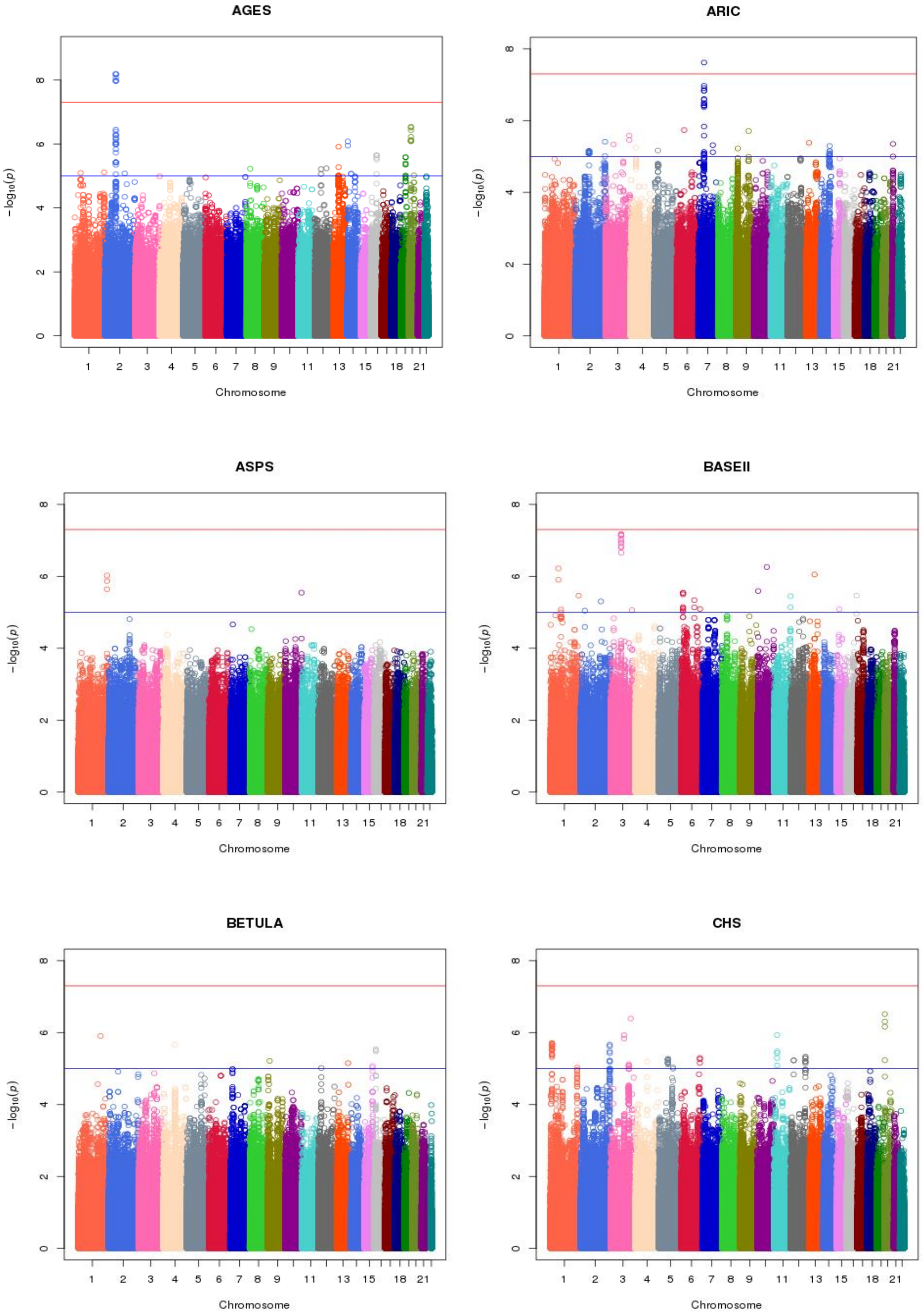

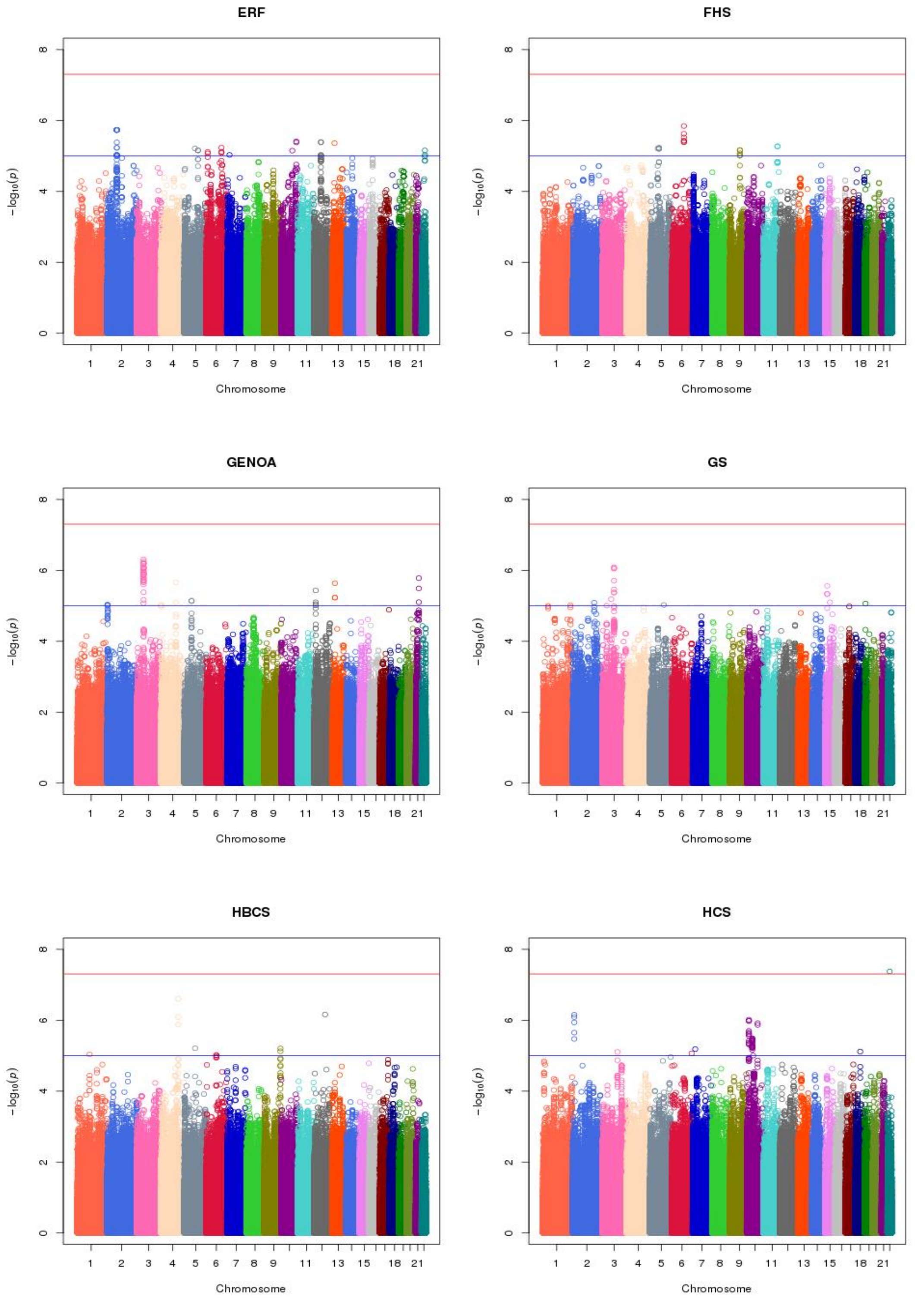
ERF
FHS
GENOA
GS
HBCS
HCS
$-\log_{10}(p)$
Chromosome
0
2
4
6
8
1
2
3
4
5
6
7
8
9
11
13
15
18
21

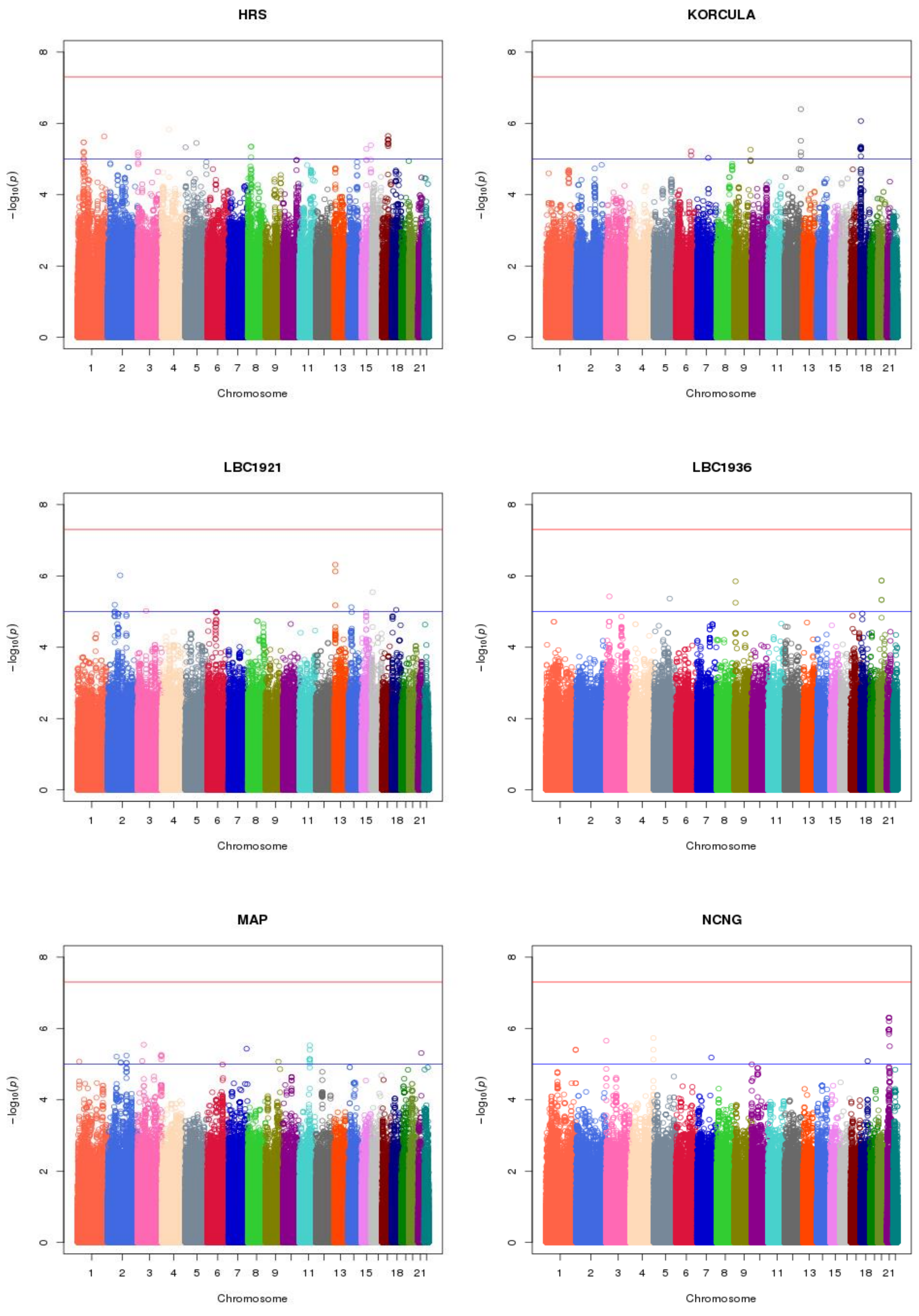
HRS
KORCULA
LBC1921
LBC1936
MAP
NCNG
$-\log_{10}(p)$
0
2
4
6
8
1 2 3 4 5 6 7 8 9 11 13 15 18 21
Chromosome

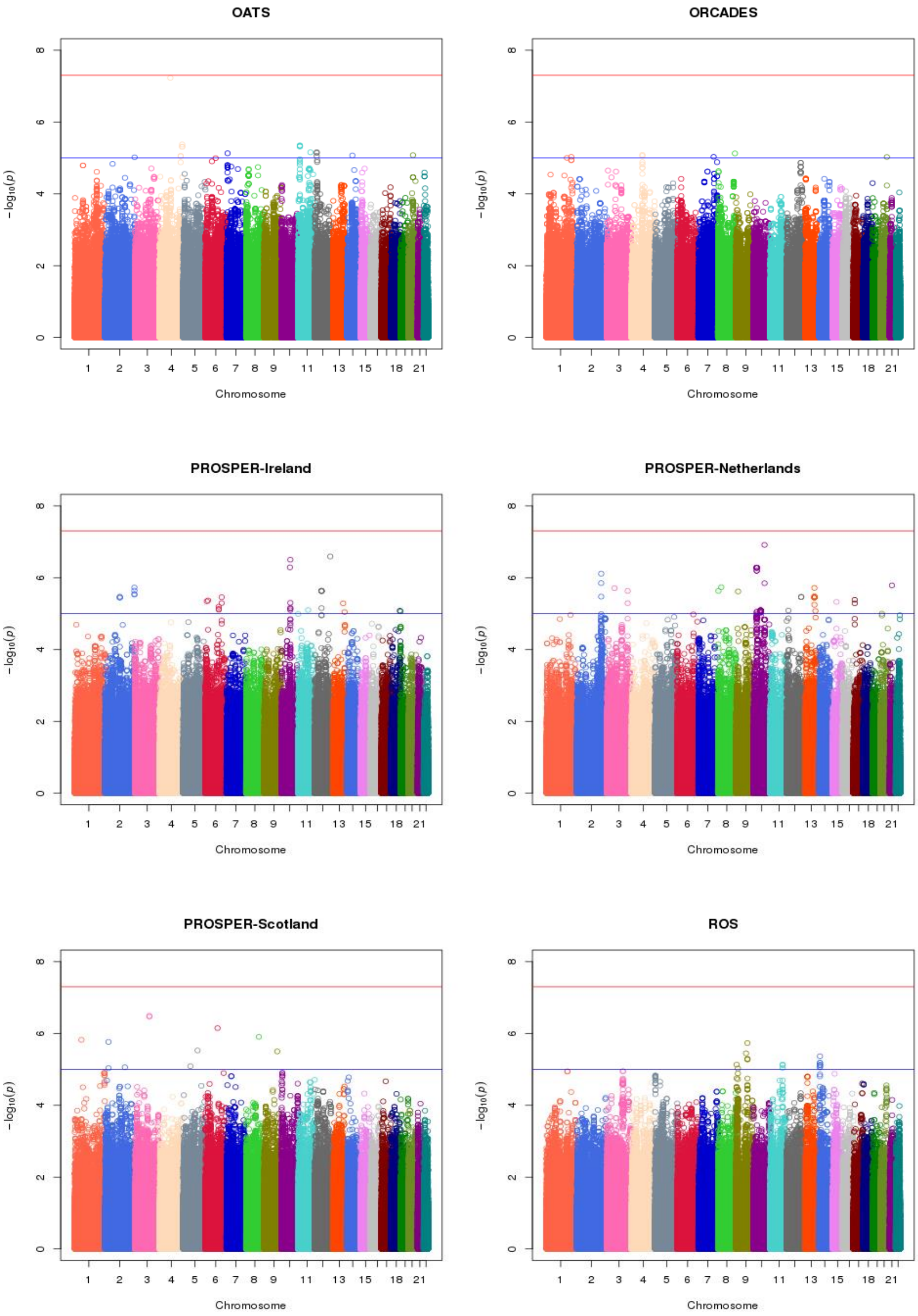
OATS
ORCADES
PROSPER-Ireland
PROSPER-Netherlands
PROSPER-Scotland
ROS
$-\log_{10}(p)$
Chromosome
0
2
4
6
8
1
2
3
4
5
6
7
8
9
11
13
15
18
21

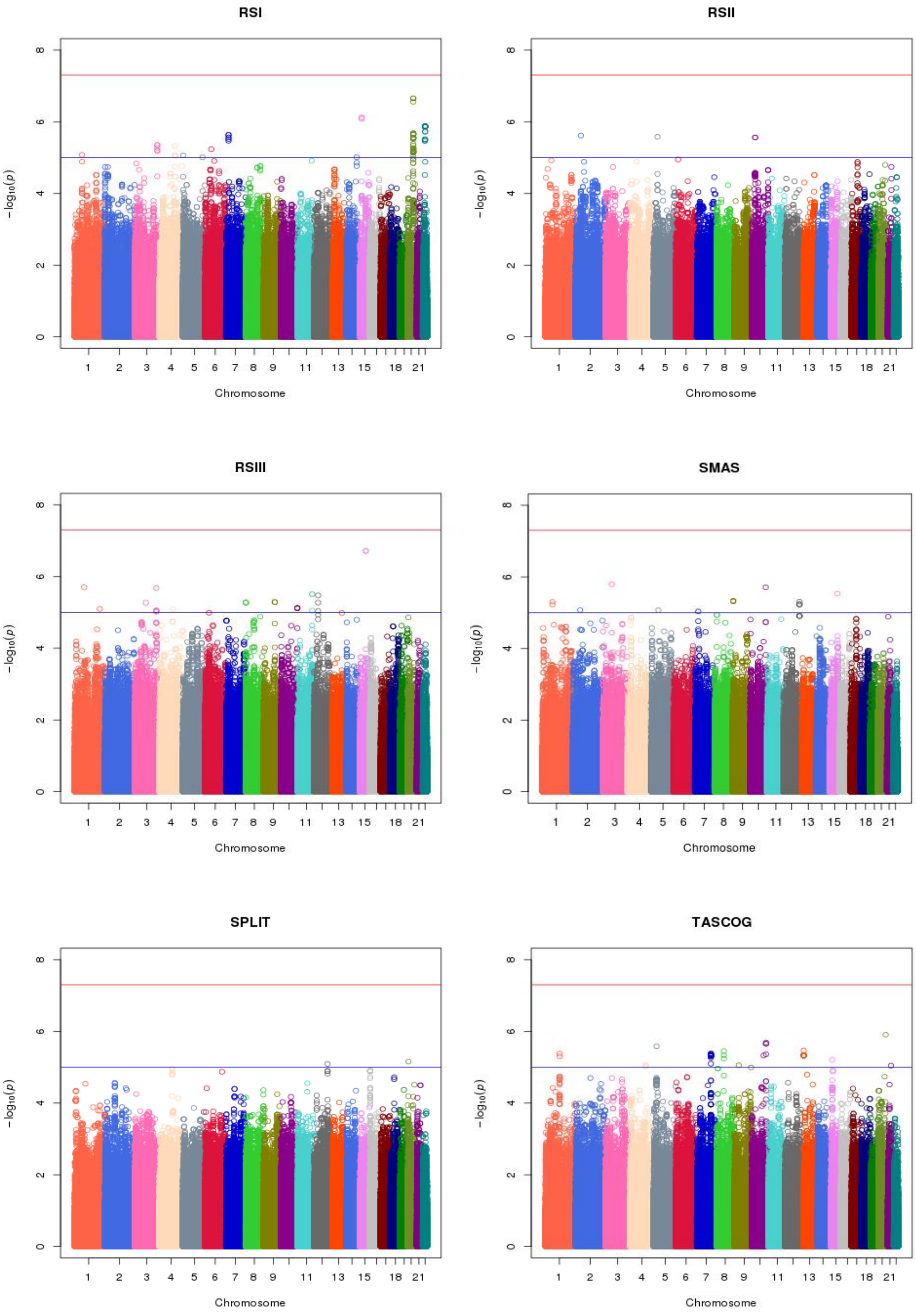
RSI
RSII
RSIII
SMAS
SPLIT
TASCOG
$-\log_{10}(p)$
Chromosome
1 2 3 4 5 6 7 8 9 11 13 15 18 21
0 2 4 6 8

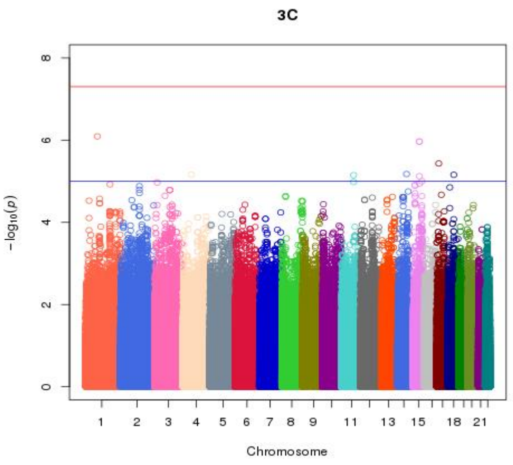
3C
8
6
4
2
0
−log10(p)
1
2
3
4
5
6
7
8
9
11
13
15
18
21
Chromosome

Supplementary Figure S2: QQ plots of the cohorts

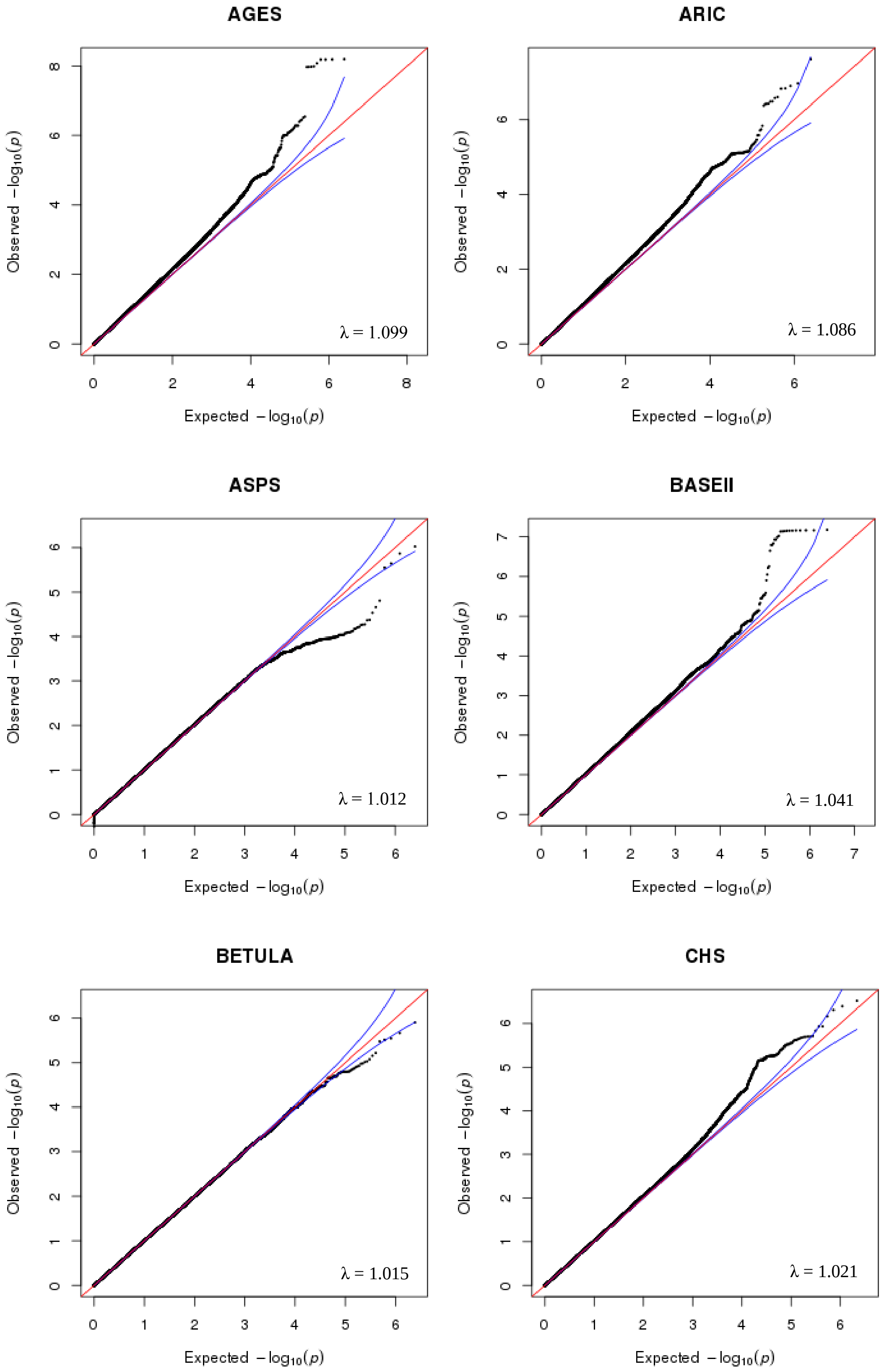

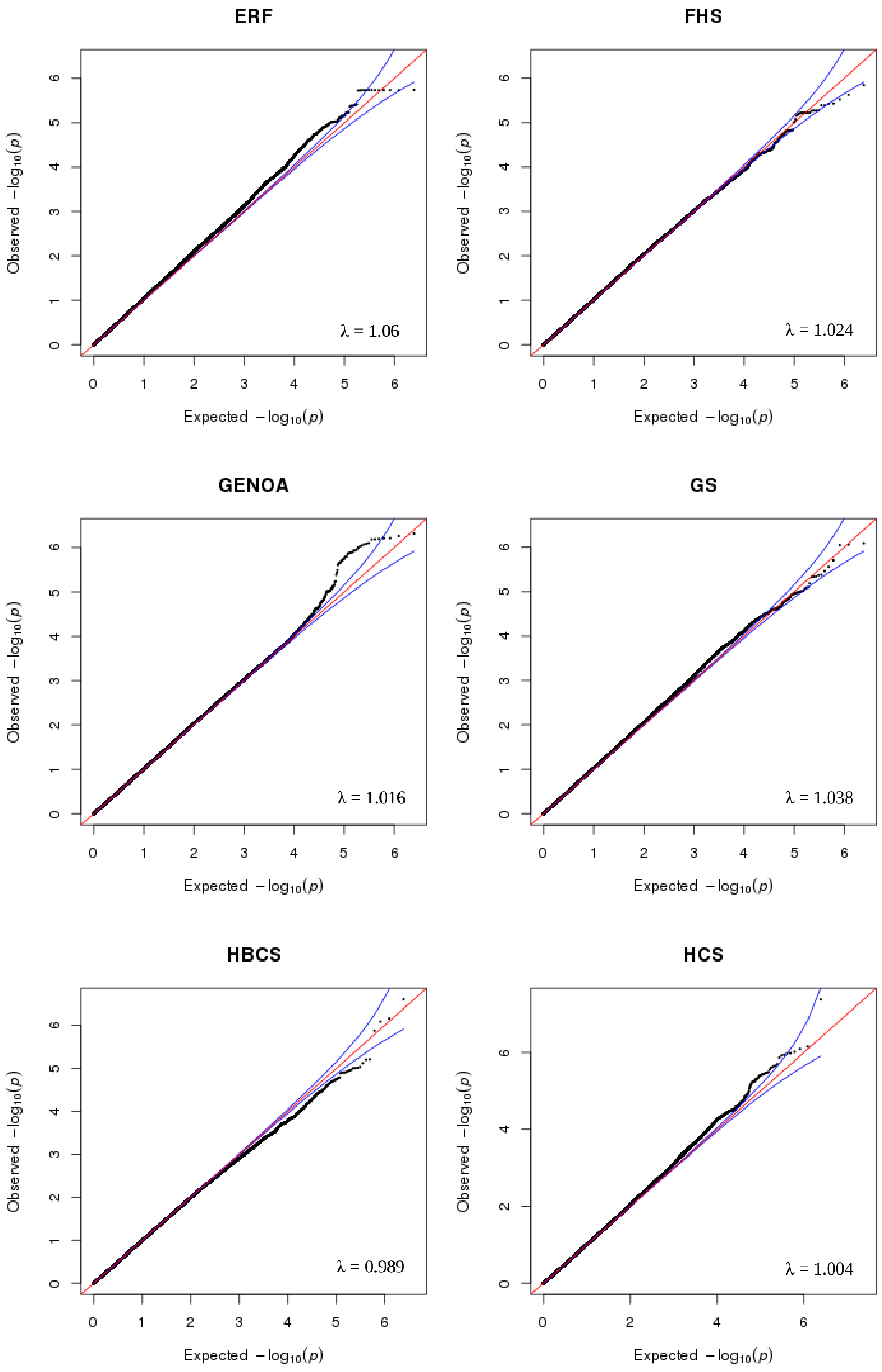
ERF
λ = 1.06
FHS
λ = 1.024
GENOA
λ = 1.016
GS
λ = 1.038
HBCS
λ = 0.989
HCS
λ = 1.004
Observed −log10(p)
Expected −log10(p)

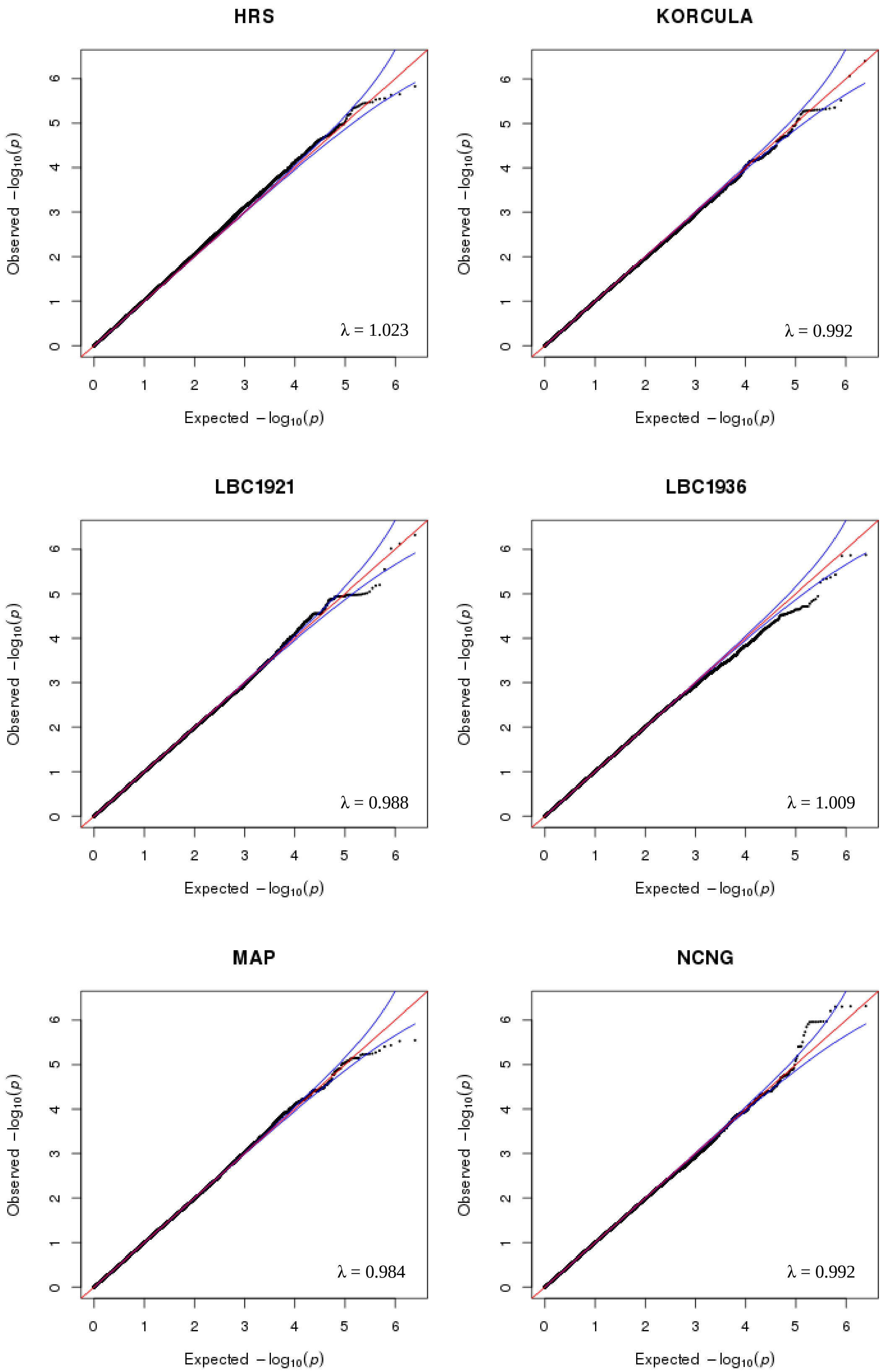
HRS
λ = 1.023
KORCULA
λ = 0.992
LBC1921
λ = 0.988
LBC1936
λ = 1.009
MAP
λ = 0.984
NCNG
λ = 0.992
Observed $-\log_{10}(p)$
Expected $-\log_{10}(p)$

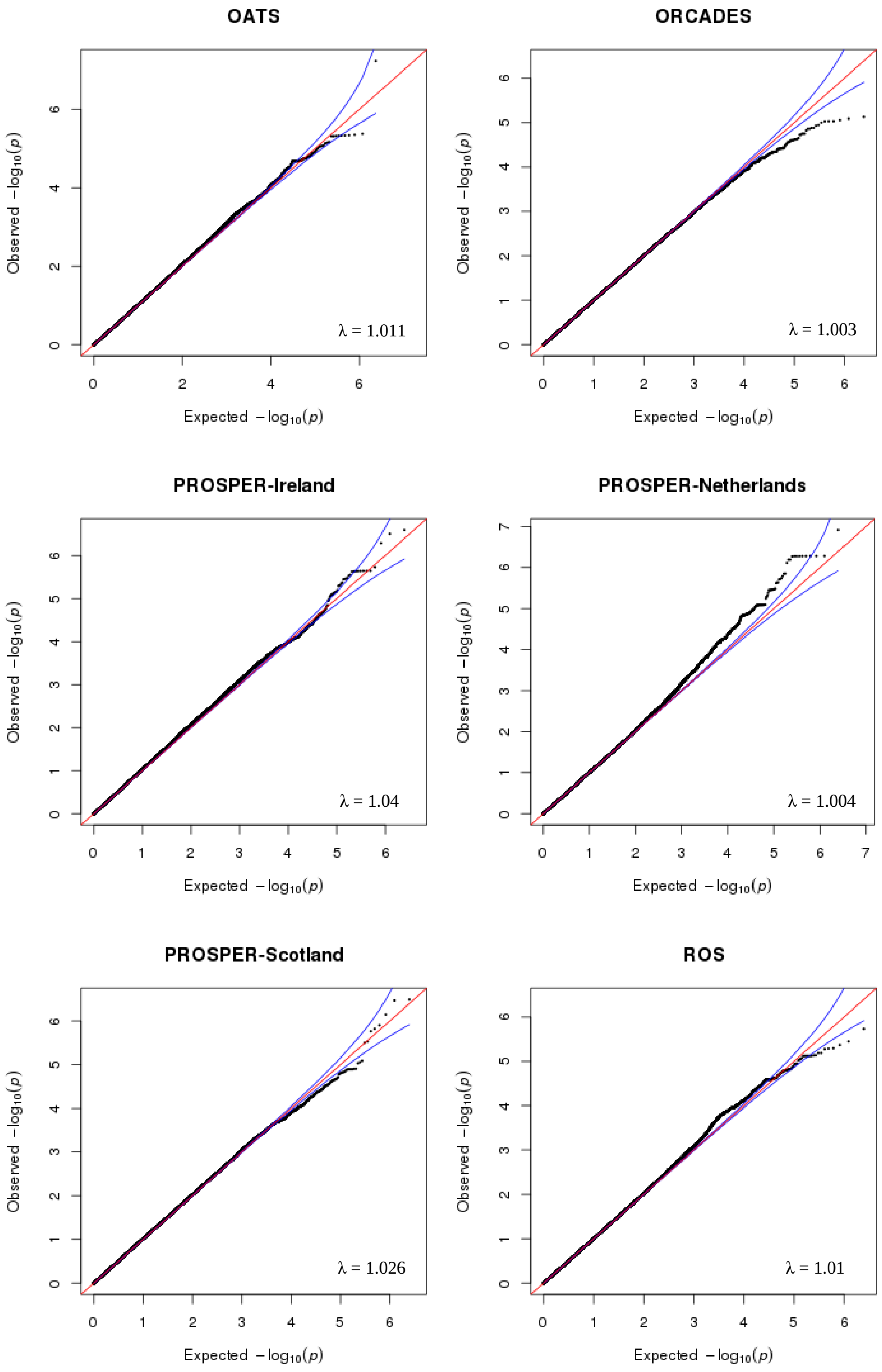
OATS
λ = 1.011
ORCADES
λ = 1.003
PROSPER-Ireland
λ = 1.04
PROSPER-Netherlands
λ = 1.004
PROSPER-Scotland
λ = 1.026
ROS
λ = 1.01
Observed −log10(p)
Expected −log10(p)

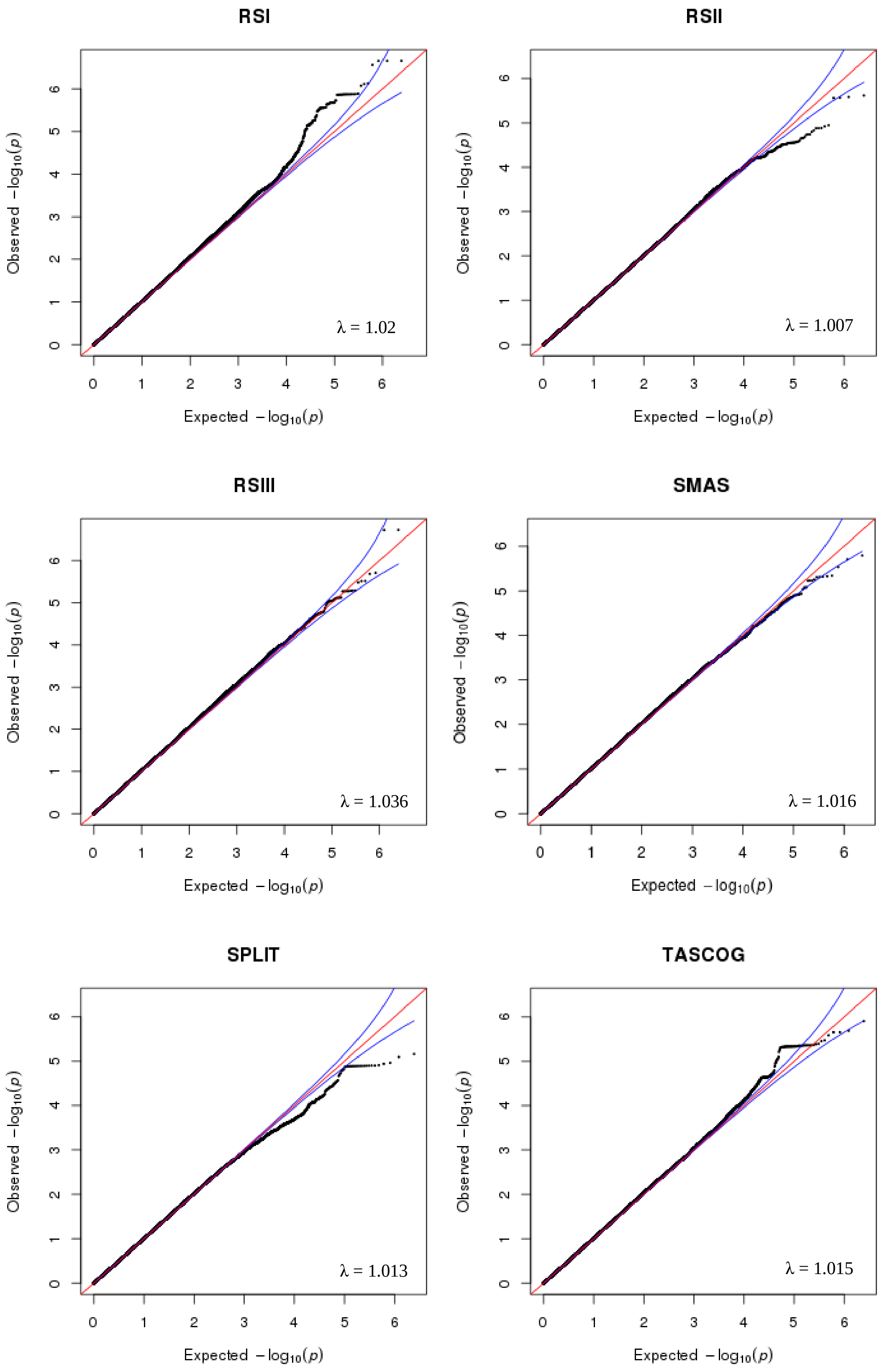
RSI
λ = 1.02
RSII
λ = 1.007
RSIII
λ = 1.036
SMAS
λ = 1.016
SPLIT
λ = 1.013
TASCOG
λ = 1.015
Observed $-\log_{10}(p)$
Expected $-\log_{10}(p)$

3C

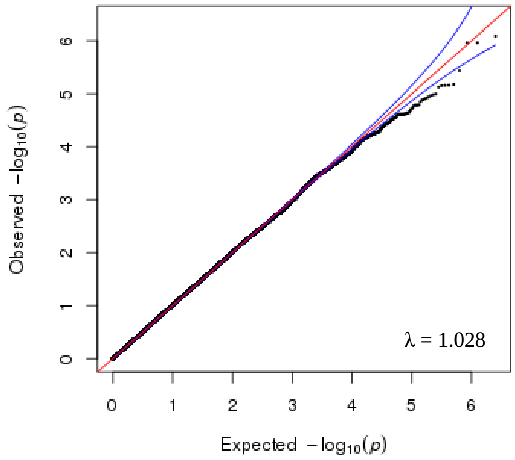

Supplementary Figure S3 Forest plots of rs10457441 (a), rs17522122 (b) and rs10119 (c).

a

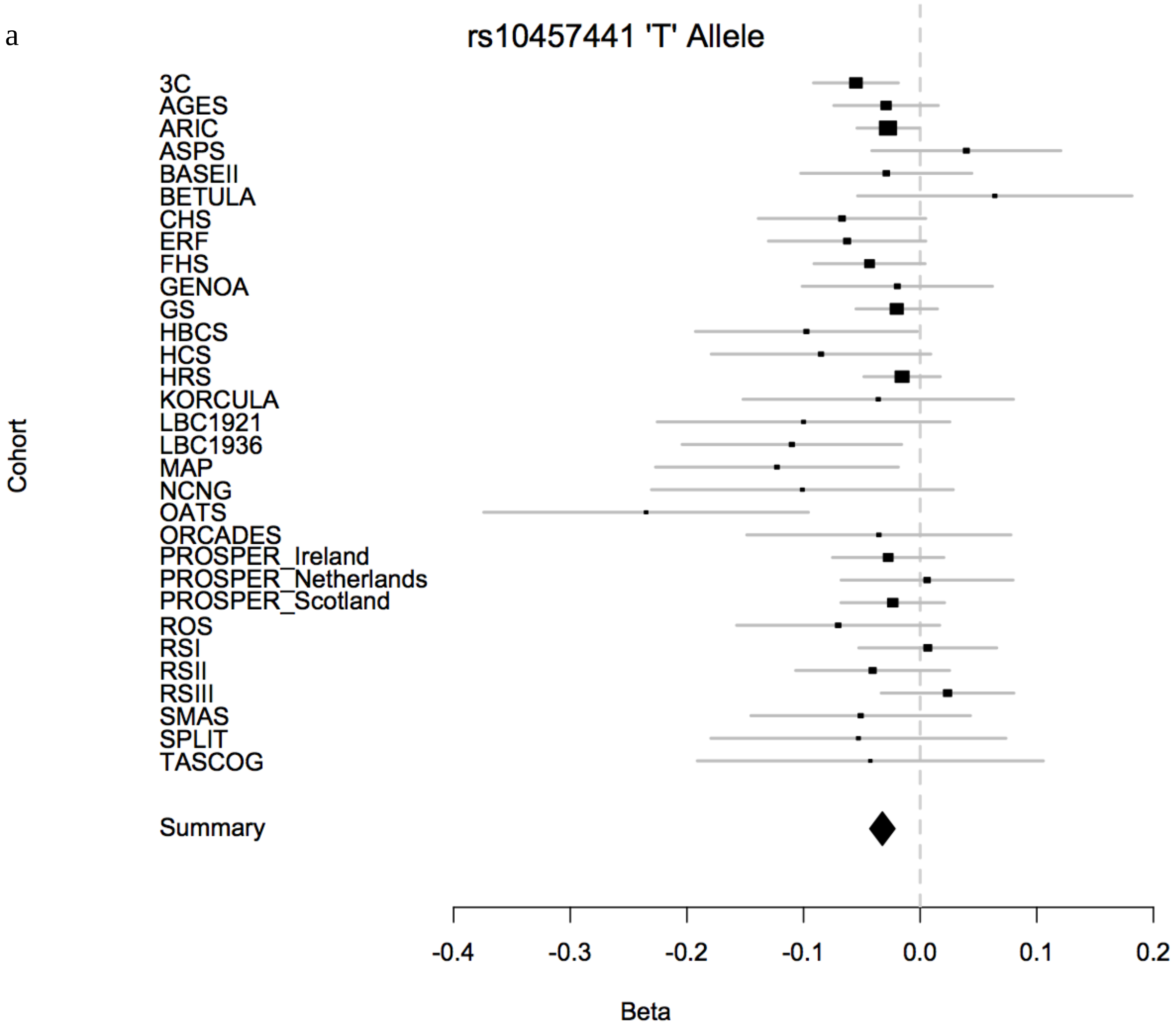

b

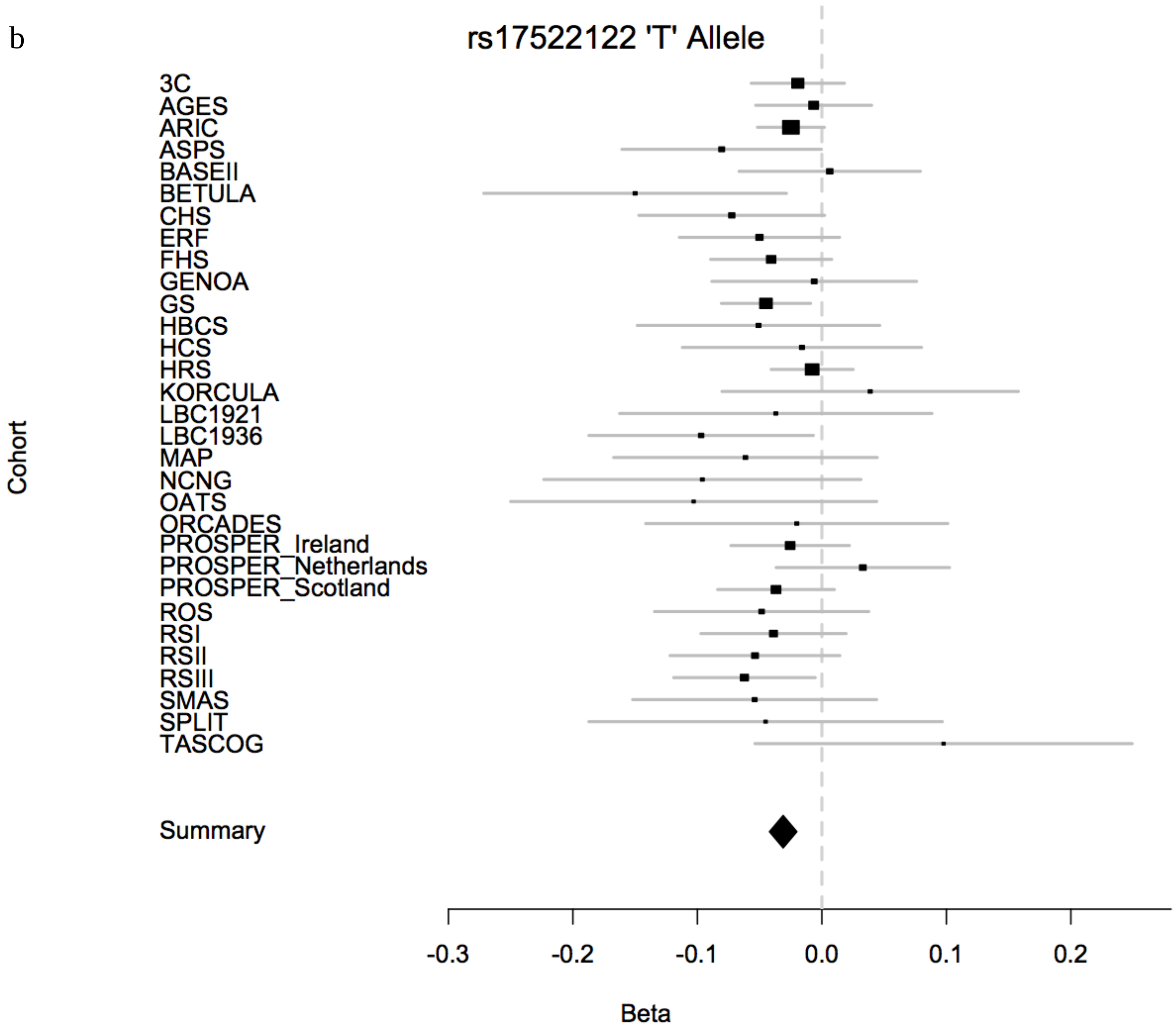
rs17522122 'T' Allele
Cohort
3C
AGES
ARIC
ASPS
BASEII
BETULA
CHS
ERF
FHS
GENOA
GS
HBCS
HCS
HRS
KORCULA
LBC1921
LBC1936
MAP
NCNG
OATS
ORCADES
PROSPER_Ireland
PROSPER_Netherlands
PROSPER_Scotland
ROS
RSI
RSII
RSIII
SMAS
SPLIT
TASCOG
Summary
-0.3
-0.2
-0.1
0.0
0.1
0.2
Beta

c

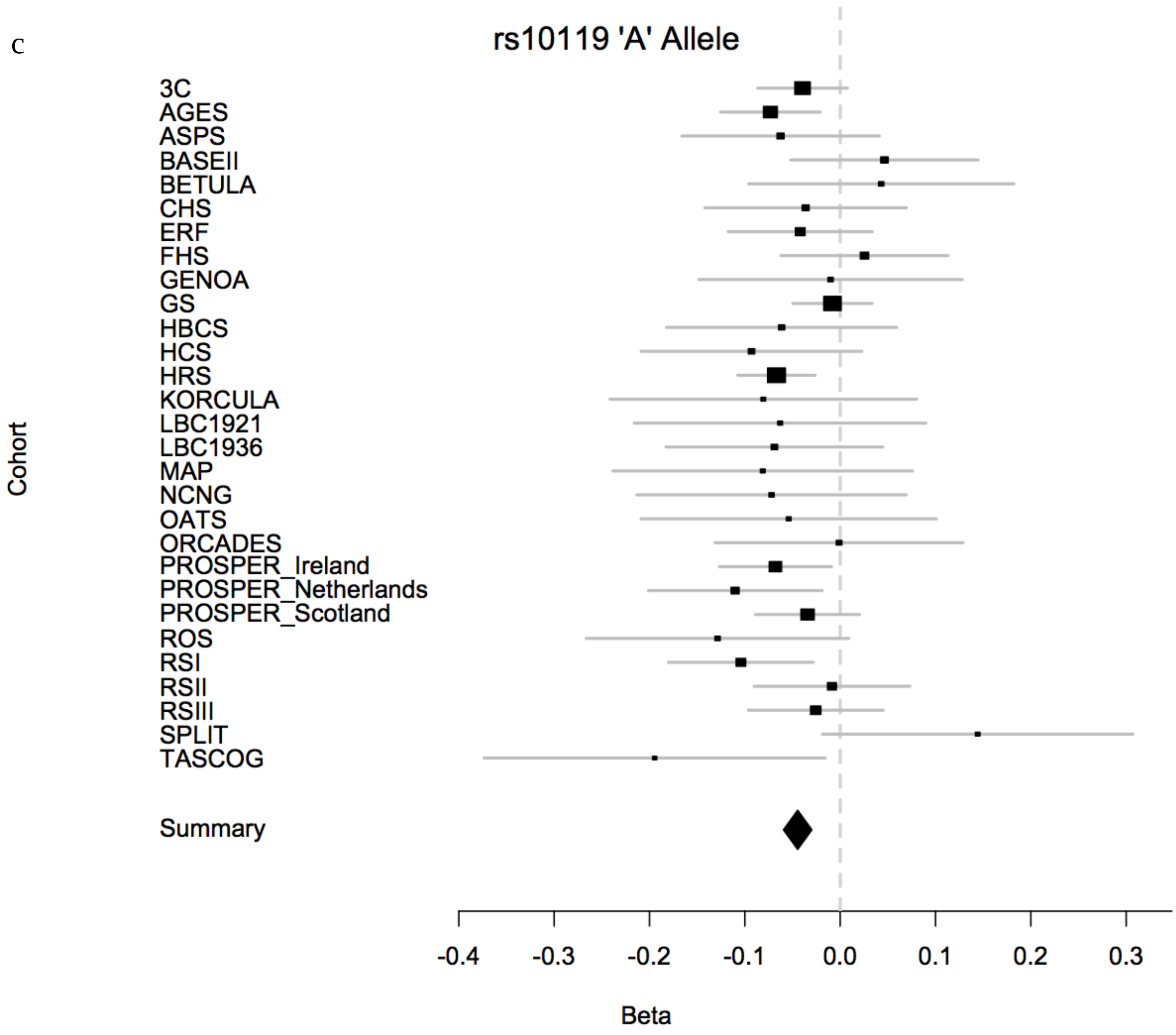
rs10119 'A' Allele
Cohort
3C
AGES
ASPS
BASEII
BETULA
CHS
ERF
FHS
GENOA
GS
HBCS
HCS
HRS
KORCULA
LBC1921
LBC1936
MAP
NCNG
OATS
ORCADES
PROSPER_Ireland
PROSPER_Netherlands
PROSPER_Scotland
ROS
RSI
RSII
RSIII
SPLIT
TASCOG
Summary
-0.4
-0.3
-0.2
-0.1
0.0
0.1
0.2
0.3
Beta

Supplementary Figure S4 Plots of effect size against mean age of cohort for rs10119. Each numbered point represents a cohort (1, RSIII; 2, ERF; 3, SPLIT; 4, GS; 5, KORCULA; 6, NCNG; 7, GENOA; 8, ORCADES; 9, RSI; 10, FHS; 11, ASPS; 12, BASEII; 13, BETULA; 14, HCS; 15, RSII; 16, HBCS; 17, LBC1936; 18, HRS; 19, OATS; 20, TASCOG; 21, 3C; 22, PROSPER-Netherlands; 23, ROS; 24, PROSPER-Scotland; 25, PROSPER-Ireland; 26, AGES; 27, LBC1921; 28, CHS; 29, MAP). Two cohorts (ARIC and Sydney MAS) did not have data available for rs10119. Plot b includes the SD of age for each cohort. Dashed regression line and shaded 95% C.I. are shown.

a

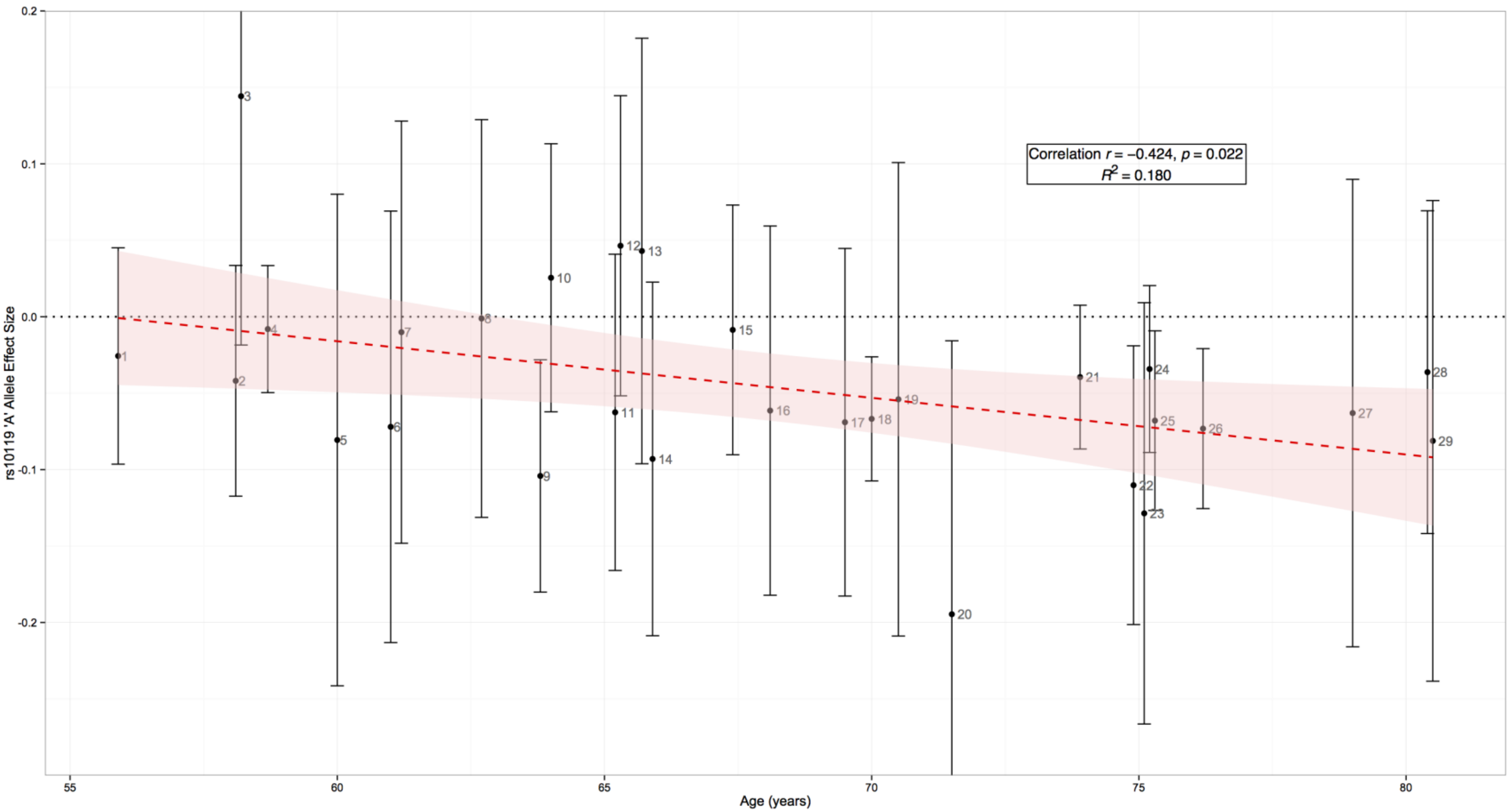

b

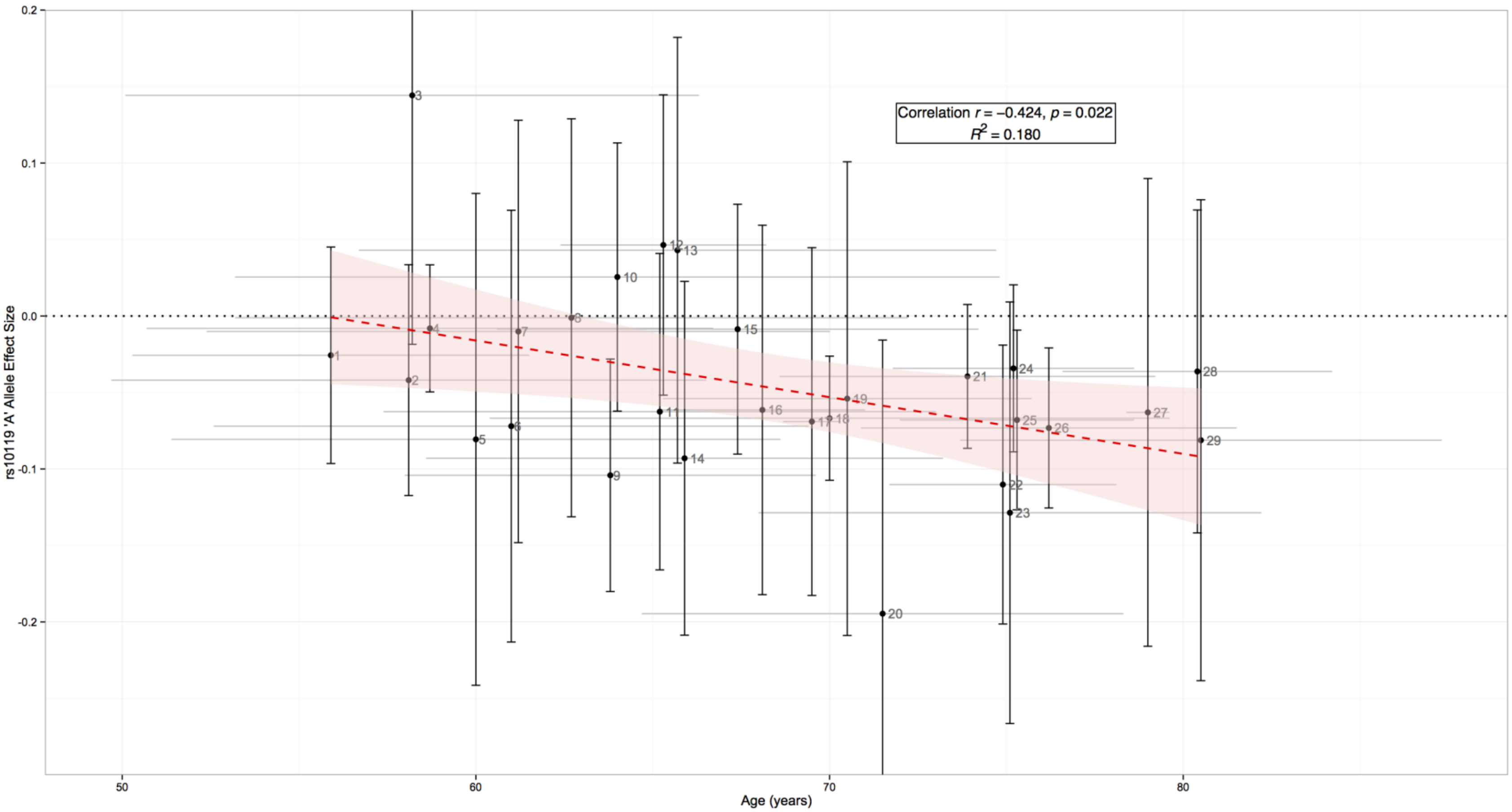

Correlation $r = -0.424$, $p = 0.022$
$R^2 = 0.180$
rs10119 'A' Allele Effect Size
Age (years)
0.2
0.1
0.0
-0.1
-0.2
50
60
70
80

Supplementary Figure S5 Plots of effect size against mean age of cohort for rs10457441. Each numbered point represents a cohort (1, RSIII; 2, ARIC; 3, ERF; 4, SPLIT; 5, GS; 6, KORCULA; 7, NCNG; 8, GENOA; 9, ORCADES; 10, RSI; 11, FHS; 12, ASPS; 13, BASEII; 14, BETULA; 15, HCS; 16, RSII; 17, HBCS; 18, LBC1936; 19, HRS; 20, OATS; 21, TASCOG; 22, 3C; 23, PROSPER-Netherlands; 24, ROS; 25, PROSPER-Scotland; 26, PROSPER-Ireland; 27, AGES; 28, Sydney MAS; 29, LBC1921; 30, CHS; 31, MAP). Plot b includes the SD of age range for each cohort. Dashed regression line and shaded 95% C.I. are shown.

a

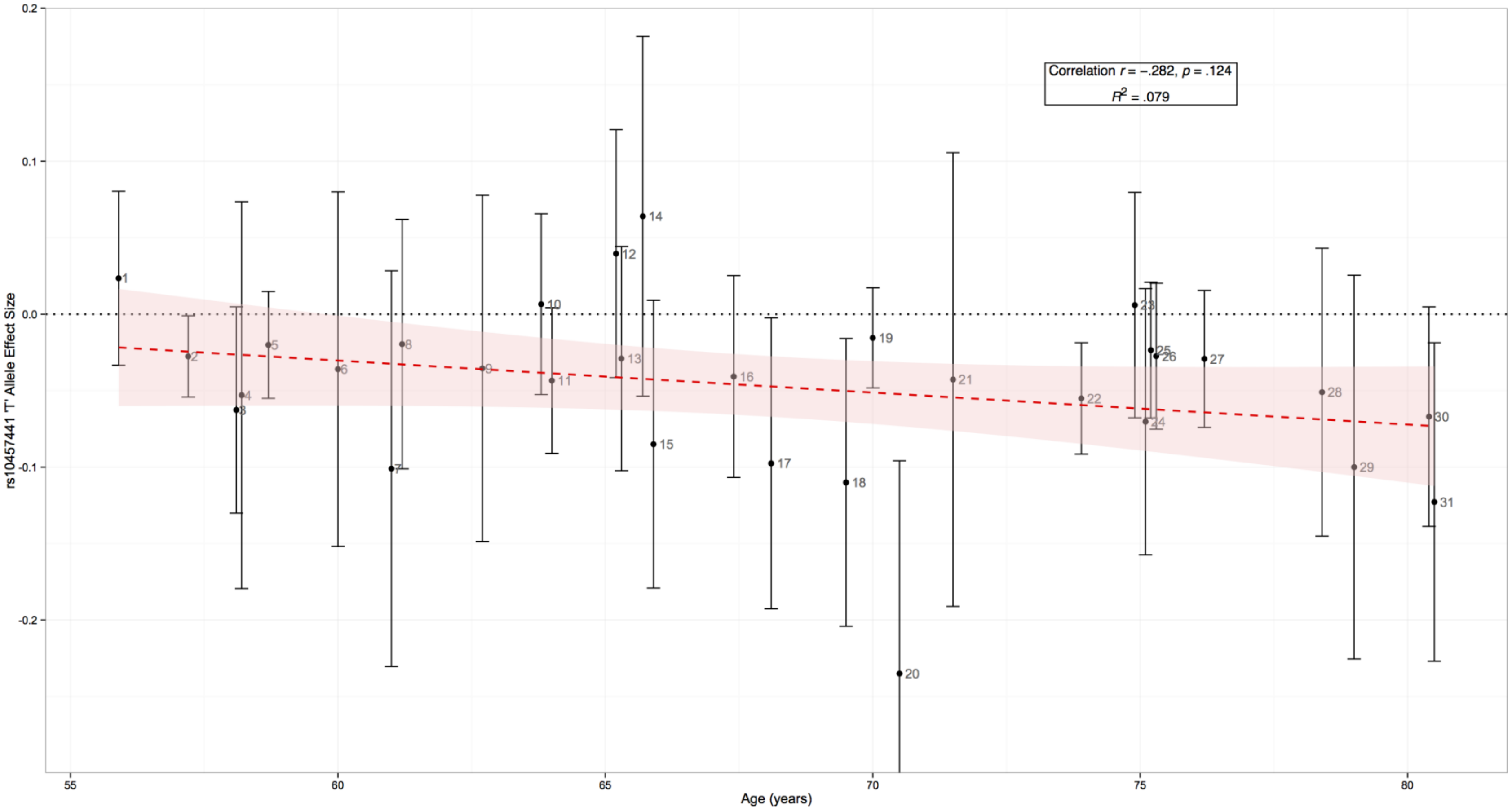

b

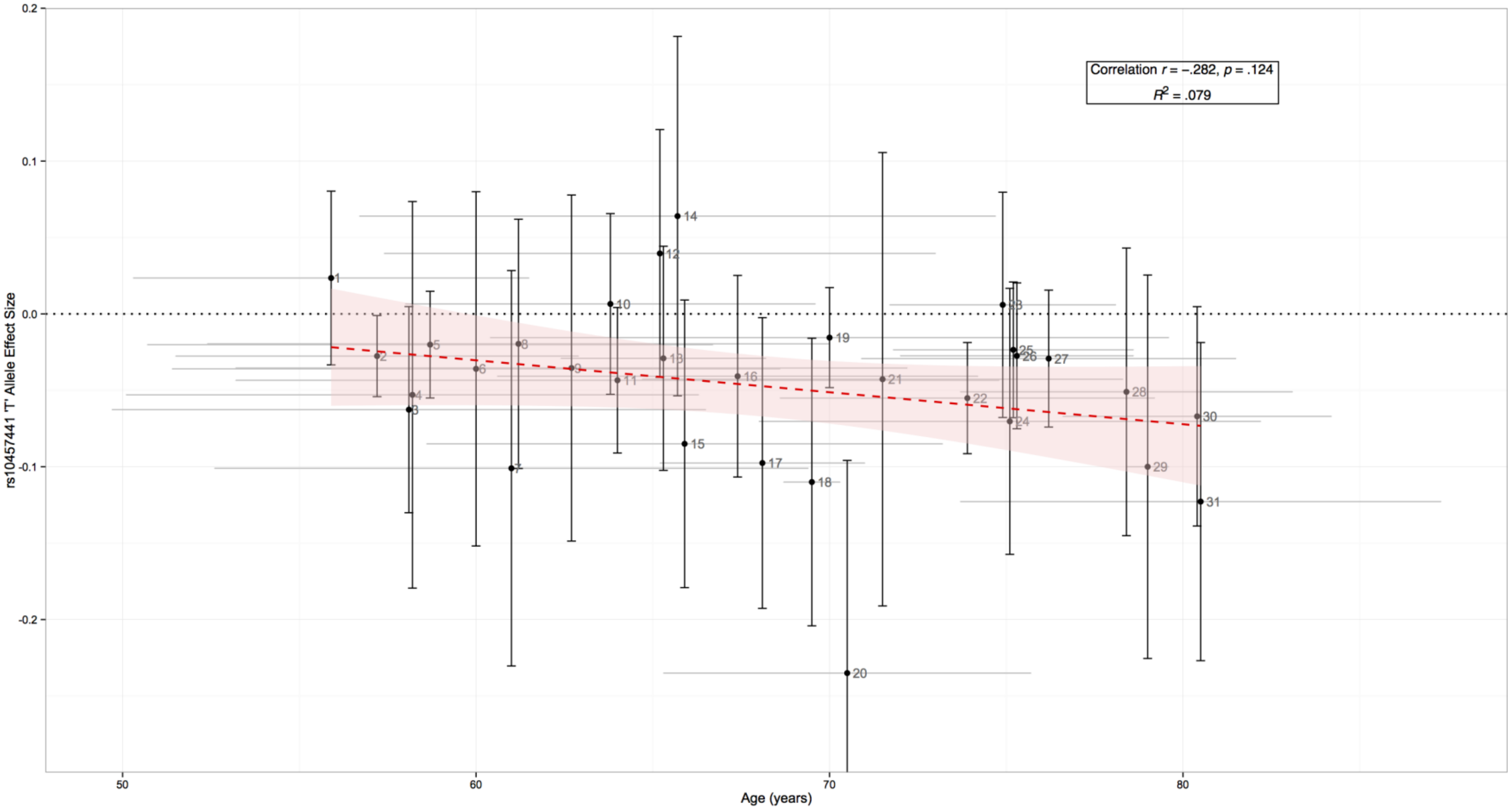
Correlation r = −.282, p = .124
R2 = .079
rs10457441 'T' Allele Effect Size
Age (years)

Supplementary Figure S6 Plots of effect size against mean age of cohort for rs17522122. Each numbered point represents a cohort (1, RSIII; 2, ARIC; 3, ERF; 4, SPLIT; 5, GS; 6, KORCULA; 7, NCNG; 8, GENOA; 9, ORCADES; 10, RSI; 11, FHS; 12, ASPS; 13, BASEII; 14, BETULA; 15, HCS; 16, RSII; 17, HBCS; 18, LBC1936; 19, HRS; 20, OATS; 21, TASCOG; 22, 3C; 23, PROSPER-Netherlands; 24, ROS; 25, PROSPER-Scotland; 26, PROSPER-Ireland; 27, AGES; 28, Sydney MAS; 29, LBC1921; 30, CHS; 31, MAP). Plot b includes the SD of age for each cohort. Dashed regression line and shaded 95% C.I. are shown.

a

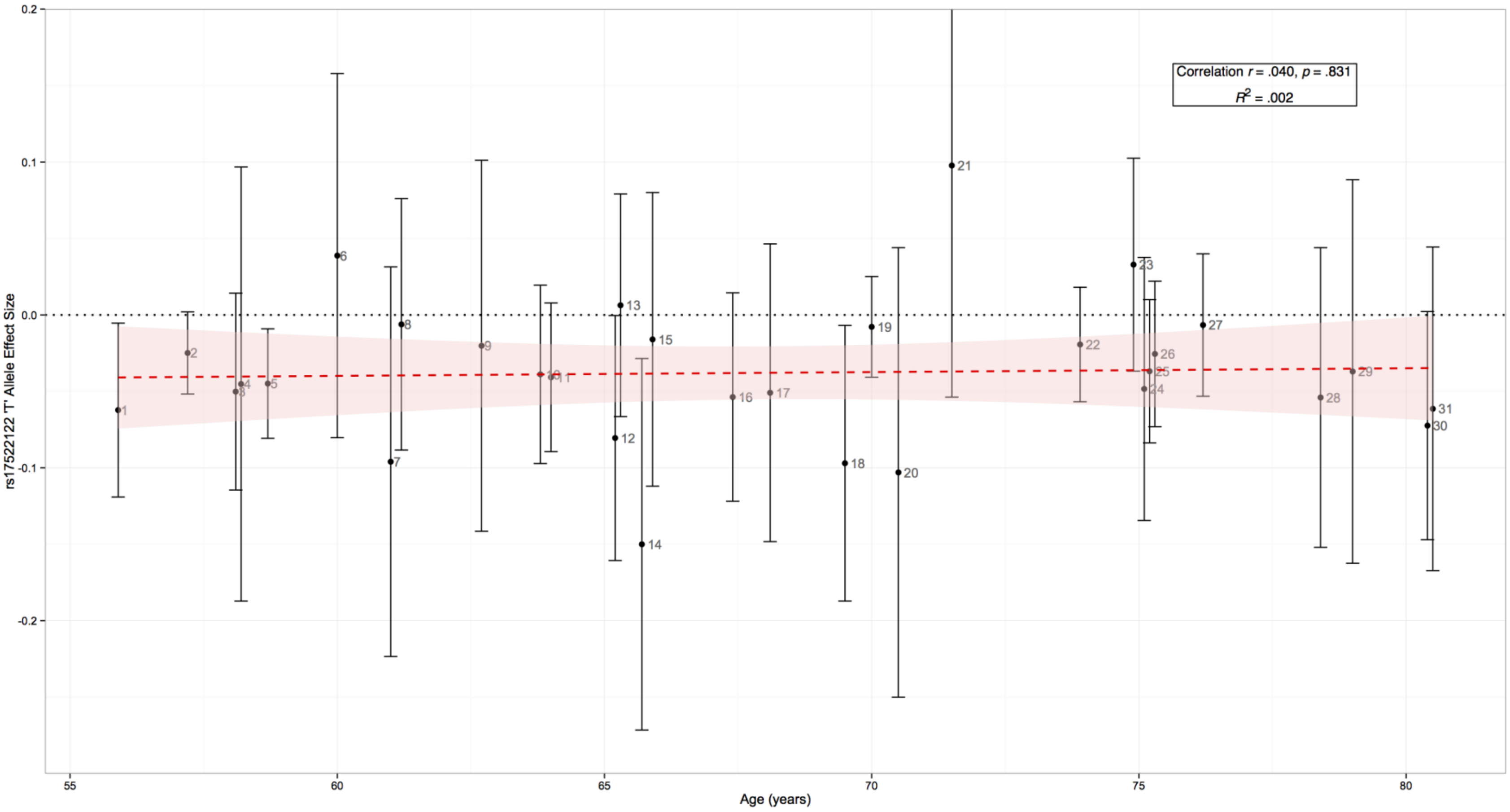

b

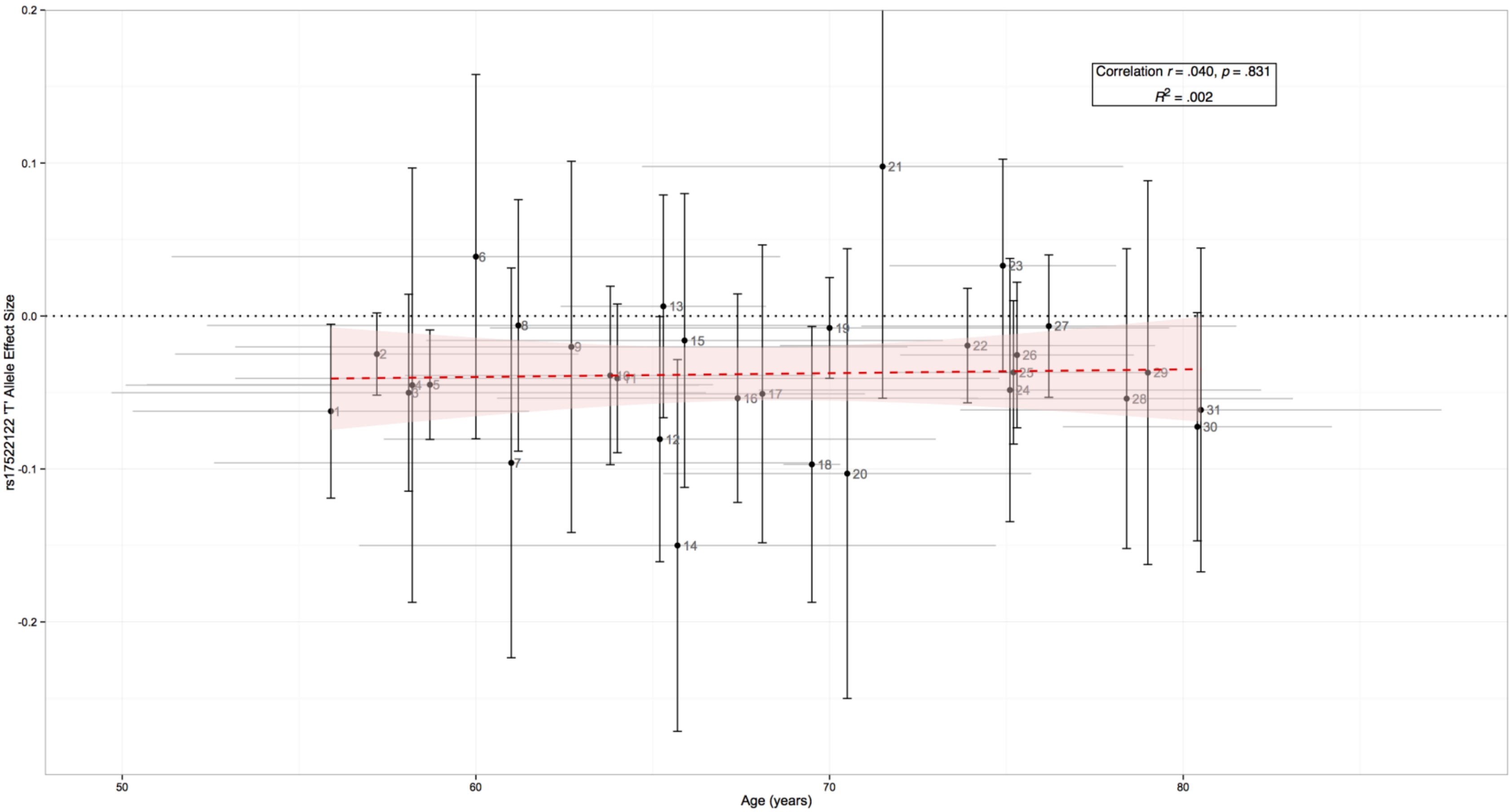
Correlation r = .040, p = .831
R^2 = .002
rs17522122 'T' Allele Effect Size
Age (years)
0.2
0.1
0.0
-0.1
-0.2
50
60
70
80

Supplementary Figure S7: IPA top 70 node network. This network is a graphical representation of the molecular relationships between the top gene-based results. The network shown had the highest IPA score (77) and included 58 focus molecules. This network is most strongly associated with the broad biofunction categories Cell Cycle, Cell Death and Survival and Cell Signalling. Genes are represented as nodes. A direct biological relationship between two nodes is represented as an edge. All edges are supported by at least 1 reference from the literature, from a textbook, or from canonical information stored in the Ingenuity Pathways Knowledge Base. Human, mouse, and rat orthologs of a gene are stored as separate objects in the Ingenuity Pathways Knowledge Base, but are represented as a single node in the network. Higher intensity node colour indicates greater significance of the gene according to the VEGAS output. Nodes are displayed using various shapes that represent the functional class of the gene product.

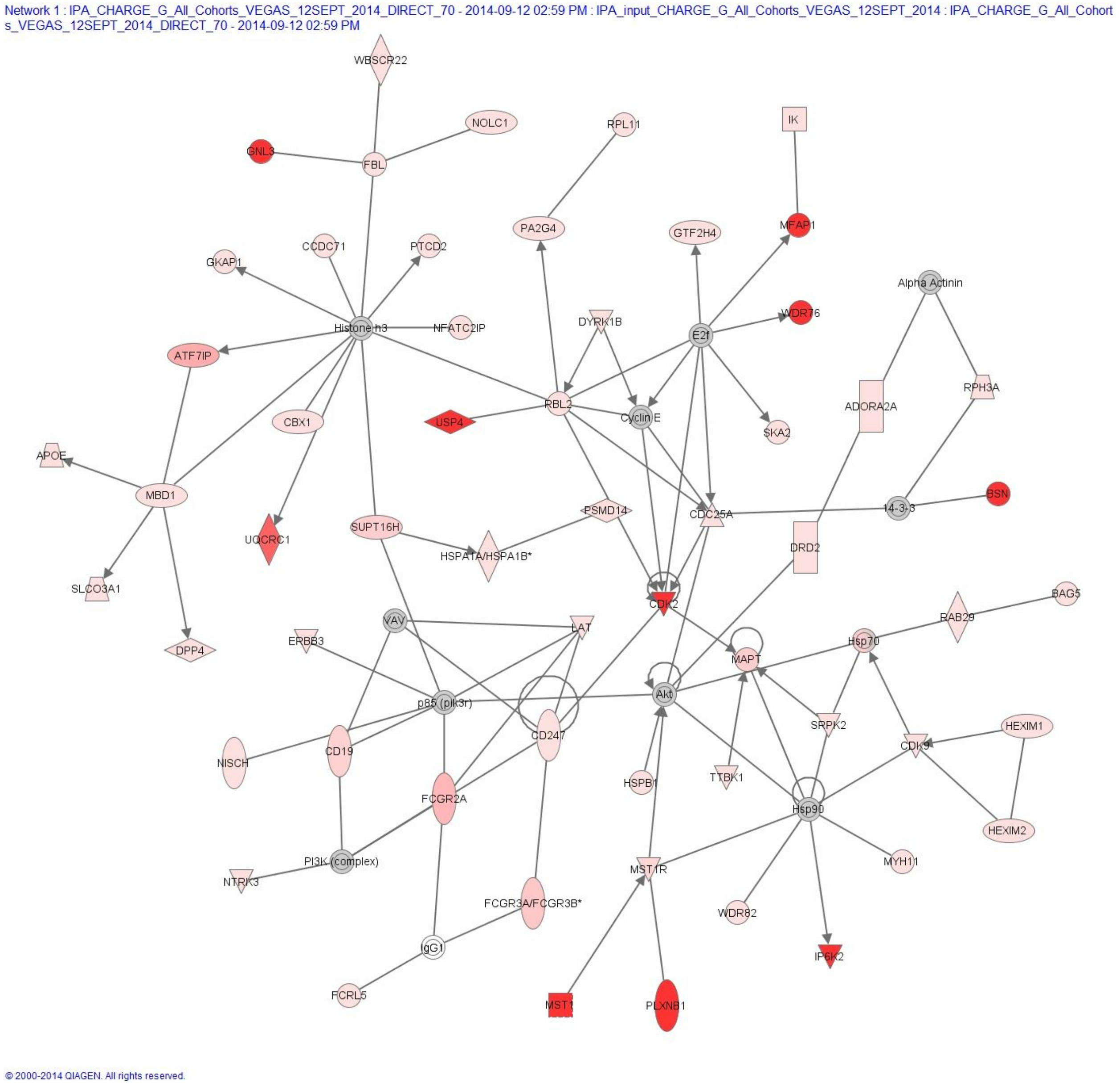

Supplementary Figure S8: IPA top 140 node network. This network is a graphical representation of the molecular relationships between the top gene-based results. The network shown had the highest IPA score (130) and included 103 focus molecules. This network is most strongly associated with the broad biofunction categories “Cell Cycle”, “Cell Death and Survival” and “Gene Expression”. Genes are represented as nodes. A direct biological relationship between two nodes is represented as an edge. All edges are supported by at least 1 reference from the literature, from a textbook, or from canonical information stored in the Ingenuity Pathways Knowledge Base. Human, mouse, and rat orthologs of a gene are stored as separate objects in the Ingenuity Pathways Knowledge Base, but are represented as a single node in the network. Higher intensity node colour indicates greater significance of the gene according to the VEGAS output. Nodes are displayed using various shapes that represent the functional class of the gene product.

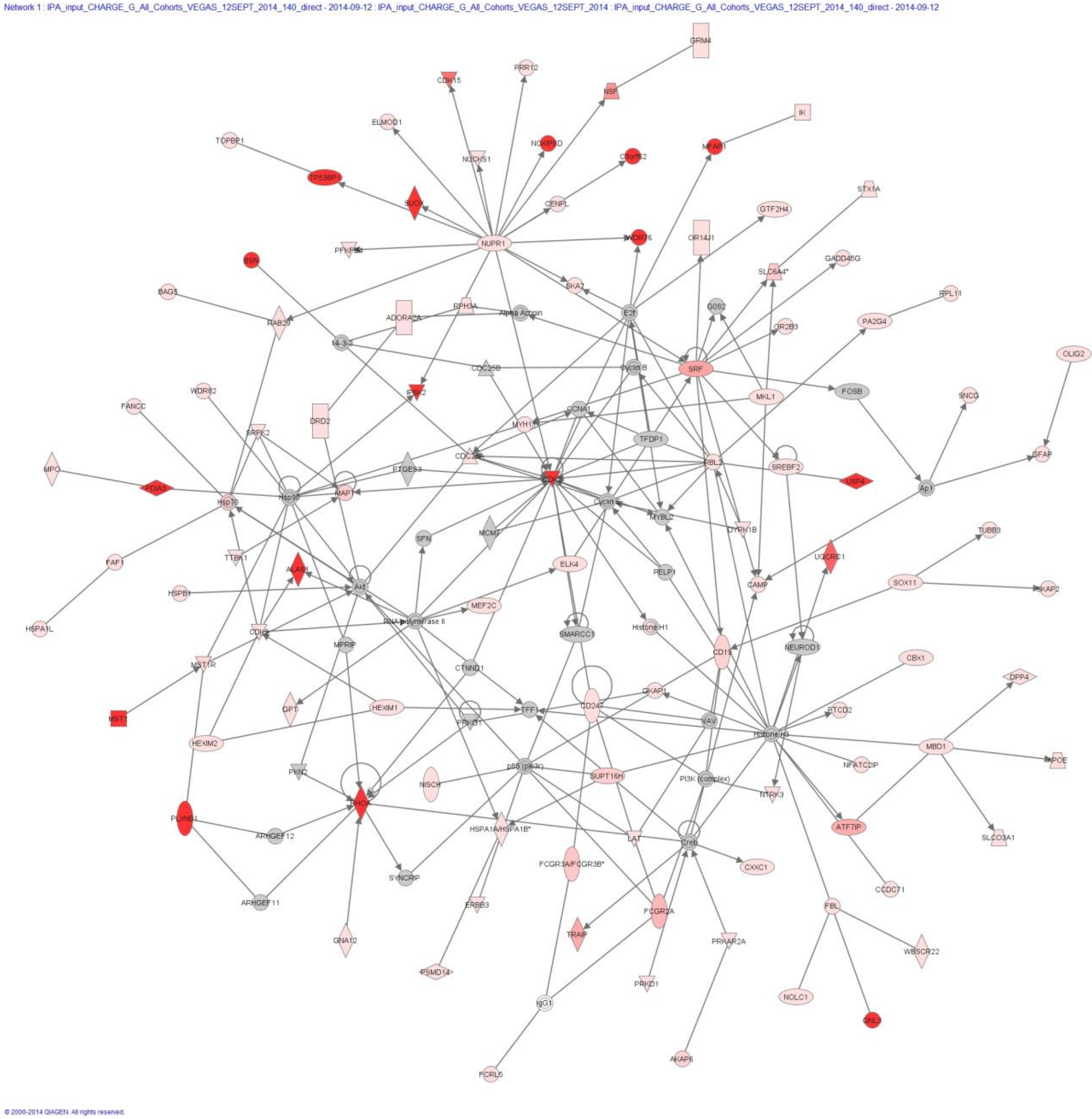

Supplementary Figure S9 Differential expression of the top genes from the VEGAS analyses in the human brain across the lifetime. Developmental age in days shown on the x-axis; mRNA expression signal intensity (log2) shown on the y-axis. Abbreviations for brain regions: NCX, neocortex; STR, striatum; HIP, hippocampus; MD, mediodorsal nucleus of the thalamus; AMY, amygdala; CBC, cerebellar cortex. Data and figure accessed from the Human Brain Transcriptome project (http://hbatlas.org/pages/hbtd).

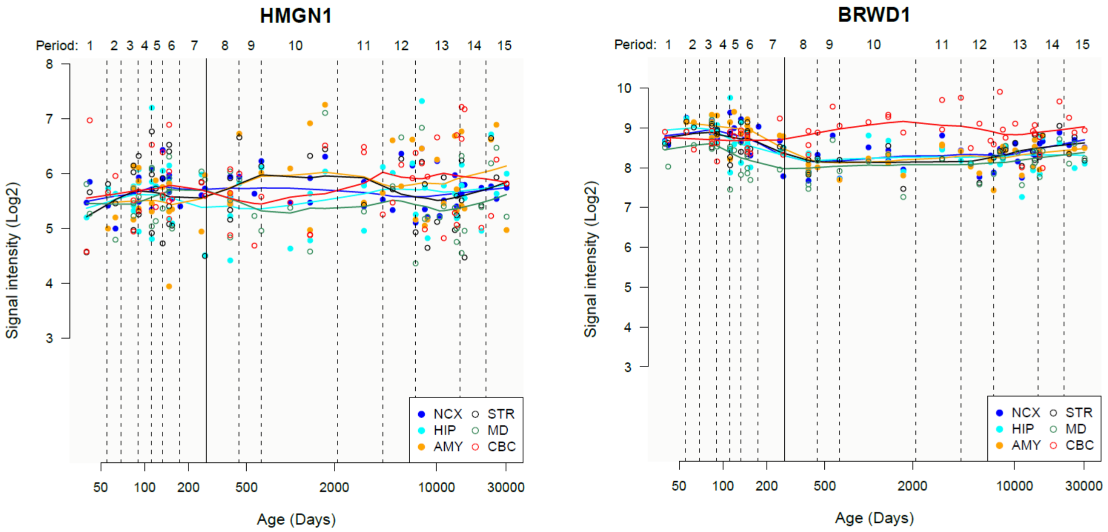

Supplement: Supplementary Information 2 [file mp2014188x2.pdf]
